# Biochemical characterization of the PHARC-associated serine hydrolase ABHD12 reveals its preference for very-long-chain lipids

**DOI:** 10.1074/jbc.RA118.005640

**Published:** 2018-09-20

**Authors:** Alaumy Joshi, Minhaj Shaikh, Shubham Singh, Abinaya Rajendran, Amol Mhetre, Siddhesh S. Kamat

**Affiliations:** From the Departments of ‡ Biology and; § Chemistry, Indian Institute of Science Education and Research Pune, Pune 411008, India

**Keywords:** neurodegenerative disease, enzyme kinetics, Michaelis–Menten, lipase, subcellular fractionation, ABHD12, hydrolase, PHARC, very-long-chain lipids

## Abstract

Polyneuropathy, hearing loss, ataxia, retinitis pigmentosa, and cataract (PHARC) is a rare genetic human neurological disorder caused by null mutations to the *Abhd12* gene, which encodes the integral membrane serine hydrolase enzyme ABHD12. Although the role that ABHD12 plays in PHARC is understood, the thorough biochemical characterization of ABHD12 is lacking. Here, we report the facile synthesis of mono-1-(fatty)acyl-glycerol lipids of varying chain lengths and unsaturation and use this lipid substrate library to biochemically characterize recombinant mammalian ABHD12. The substrate profiling study for ABHD12 suggested that this enzyme requires glycosylation for optimal activity and that it has a strong preference for very-long-chain lipid substrates. We further validated this substrate profile against brain membrane lysates generated from WT and ABHD12 knockout mice. Finally, using cellular organelle fractionation and immunofluorescence assays, we show that mammalian ABHD12 is enriched on the endoplasmic reticulum membrane, where most of the very-long-chain fatty acids are biosynthesized in cells. Taken together, our findings provide a biochemical explanation for why very-long-chain lipids (such as lysophosphatidylserine lipids) accumulate in the brains of ABHD12 knockout mice, which is a murine model of PHARC.

## Introduction

Polyneuropathy, hearing loss, ataxia, retinitis pigmentosa, and cataract (PHARC)^3^ (OMIM 612674) is a rare autosomally recessive neurological disorder caused by homozygous or compound heterozygous mutations in the *Abhd12* gene on chromosome 20p11 in humans (1–4). This gene encodes the integral membrane enzyme ABHD12 (α/β hydrolase domain–containing protein 12), which belongs to the metabolic serine hydrolase family of enzymes (5, 6). The major symptoms of PHARC include polymodal sensory and motor defects linked to peripheral neuropathy, hearing loss, and early onset of cataract and eventual blindness (1, 2, 4). Other clinical symptoms include massive cerebellar atrophy and demyelination of sensorimotor neurons (1, 2). The symptoms of PHARC appear in late-childhood or early-teenage years, worsen progressively with age, and have no bias to gender or race (1, 2). To date, several mutations have been mapped in human PHARC subjects, all predicted to cause loss of ABHD12 activity and hence its biological function (1–4, 7–9). Recently, the murine model of PHARC, *i.e.* the ABHD12 knockout mouse line, was generated and extensively characterized (5). These mice exhibit age-dependent PHARC-like phenotypes, which included auditory and motor deficits (5). These mice displayed heightened neuroinflammation and cerebellar microgliosis at time points preceding the sensorimotor defects (5). Untargeted lipidomics on the brains of these mice showed elevated levels of lysophosphatidylserine (lyso-PS) class of lipids, suggesting deregulated lyso-PS signaling as a likely causative factor for the PHARC-like symptoms displayed by these mice. Of note, very-long-chain (VLC, ≥C22) lyso-PS lipids were massively accumulated in the brains of these mice (5). These experiments thus suggest that lyso-PS lipids are possibly the *in vivo* substrates of ABHD12.

**Figure 1.**
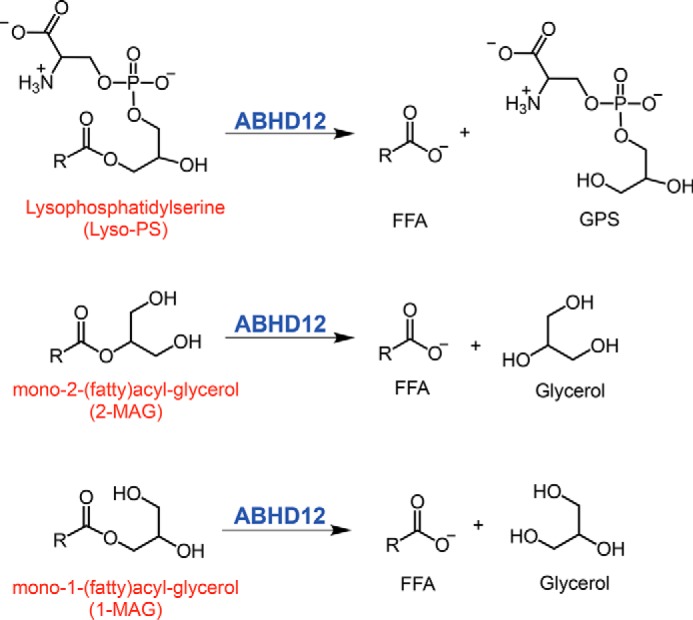
**The *in vitro* substrates and the lipase reactions catalyzed by mammalian ABHD12.** In each case, ABHD12 releases a free fatty acid (*FFA*) product from the lipid substrate it hydrolyzes. For lyso-PS lipids, the other product is glycerophosphoserine (*GPS*), whereas for 1-MAG and 2-MAG, the other product is glycerol.

Although ABHD12 prefers lyso-PS lipids as substrates *in vivo*, virtually no studies are available that describe the thorough biochemical characterization of ABHD12 using lyso-PS lipids as substrates. This is mostly because only a handful of lyso-PS lipids are available commercially, and even these are very expensive to perform such studies. Additionally, there are currently no facile synthetic routes to generate lyso-PS lipids, thereby limiting their use as substrates for biochemical characterization of this enzyme. However, ABHD12 hydrolyzes other lipids *in vitro*, making them suitable surrogate substrates for such biochemical substrate profiling studies. Previous studies have shown that ABHD12 can robustly hydrolyze mono-(fatty)acyl-glycerol (MAG) substrates *in vitro* (10–12). In fact, ABHD12 was first annotated as a MAG lipase, given its ability to robustly hydrolyze the endocannabinoid 2-arachidonoyl-glycerol (2-AG) (10). Since then, another study shows that mammalian ABHD12 can use both 2-AG and 1-arachidonoyl-glycerol at comparable rates, with a slight preference for 1-arachidonoyl-glycerol as an *in vitro* substrate (12). The same study also describes that mammalian ABHD12 can use other long lipid chain containing mono-1-(fatty)acyl-glycerol (1-MAG) and mono-2-(fatty)acyl-glycerol (2-MAG) lipid substrates at comparable enzymatic rates (12). All *in vitro* substrate profiling studies taken together, show that mammalian ABHD12 does not use phospholipids, diacylglycerols, or triacylglycerols lipids as substrates, thus limiting the substrate scope of this enzyme to only lyso-PS, 2-MAG, and 1-MAG lipids (Fig. 1) (11, 12).

Although these pioneering biochemical studies describe the substrate scope of mammalian ABHD12, it is important to note that all these substrate-profiling studies were done at a single substrate concentration (25 or 100 μm), and rigorous Michaelis–Menten type enzyme kinetic studies on mammalian ABHD12 are lacking for any substrate except 2-AG to the best of our knowledge (12). Also, these single concentration substrate-profiling studies were done for only for medium (C8–C12) and long chain (C14–C20) fatty acid MAG substrates, and VLC (≥C22) fatty acid containing MAG substrates have not been tested against any mammalian ABHD12 to the best of our knowledge. This again, is because VLC-containing MAG lipids are not easily available from commercial sources and/or are very expensive for such studies.

Given the lack of a thorough biochemical characterization, we decided to perform a rigorous substrate profiling study of mammalian ABHD12. Toward this, here, we describe a facile synthetic route to generate 1-MAG lipids substrates of varying chain lengths and unsaturation. We assay these 1-MAG lipid substrates against recombinant (human) and endogenous (mouse brain) mammalian ABHD12 and generate a broad substrate profile for mammalian ABHD12. Lastly, we are the first to report the cellular localization of ABHD12 using biochemical cellular organelle fraction and immunofluorescence studies, and taken together our studies posit a biochemical explanation for the VLC lipid (lyso-PS) accumulation seen in the brains of ABHD12 knockout mice, the murine model of PHARC (5).

## Results

### Synthesis of 1-MAG lipid library

**Figure 2.**
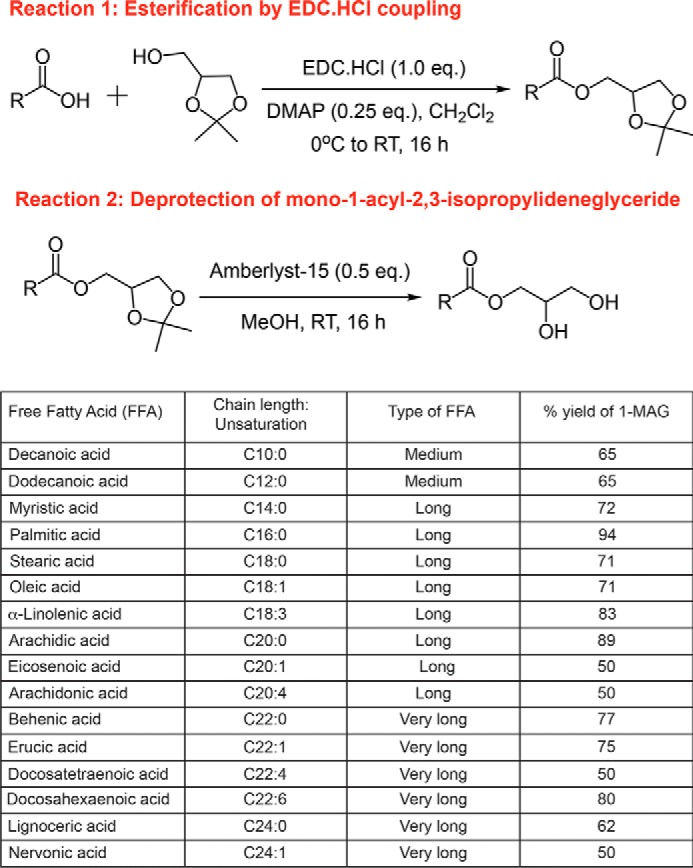
**Facile synthetic route for generating 1-MAG lipids.** 1-MAG lipids were generated using a two-step synthesis. The first step was an EDC coupling reaction to esterify the fatty acid of interest to a protected glycerol moiety, and the second step was a deprotection reaction using Amberlyst catalyst to yield 1-MAG lipids. The corresponding yields of each reaction are tabulated in the figure.

To assess the 1-MAG substrate preference of mammalian ABHD12, we decided to synthesize a library of 1-MAG lipids of varying fatty acid chain lengths and differing extents of unsaturation. We wanted the synthesis route to be relatively easy, possible at a small scale (low milligram) using commercially available free fatty acids, and able to generate good reaction yields. Toward this, we adapted a two-step synthetic scheme from known and well-reported synthetic reactions (Fig. 2). In the first step, using 1-ethyl-3-(3-dimethylaminopropyl)carbodiimide (EDC) as a coupling reagent, esterification of a free fatty acid (from C10 to C24) with 1,2-isopropylideneglycerol was performed (13). The second step involved the deprotection of the isopropylidene group using the Amberlyst-15 catalyst to yield the corresponding 1-MAG lipid of interest (14). All reactions were performed on a 10- or 20-mg scale depending on the cost and availability of the starting free fatty acid, and these reactions afforded yields from 50 to 94% (Fig. 2). The complete synthesis description and detailed compound characterization details are available in the supporting information.

### Effect of glycosylation on the activity of human ABHD12 (hABHD12)

**Figure 3.**
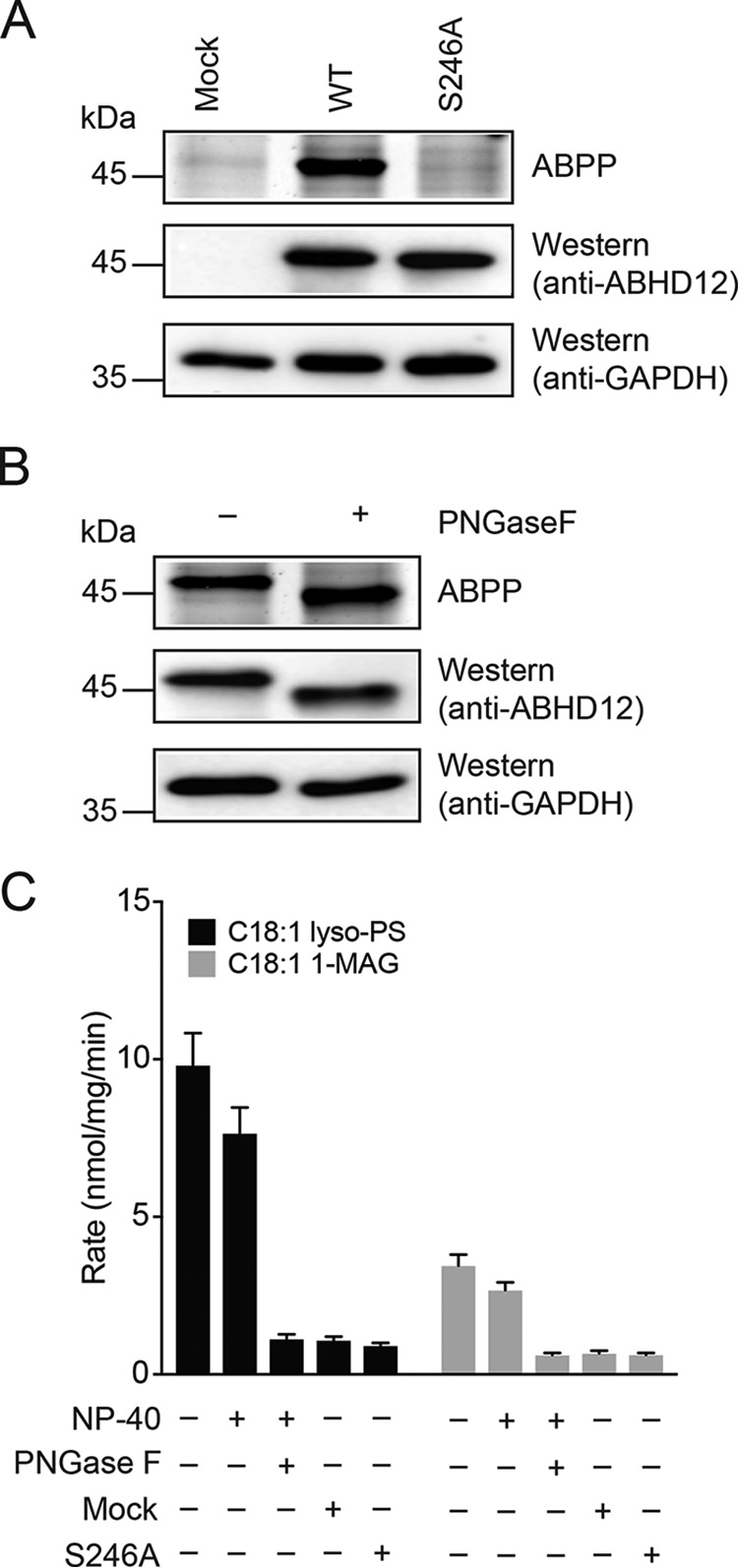
**Glycosylation is needed for optimal hABHD12 activity.** *A*, the activity of WT hABHD12, but not S246A hABHD12, was confirmed by gel-based ABPP assays on membrane lysates of HEK293T transfected with WT or S246A hABHD12. The expression over mock of both WT and S246A hABHD12 was confirmed by Western blotting analysis. GAPDH was used as a loading control in these experiments. This experiment was done in biological triplicate with reproducible results. *B*, PNGase F treatment confirms the glycosylation (migration to a lower molecular weight band) of overexpressed WT hABHD12 by gel-based ABPP and Western blotting analysis. For the gel-based ABPP assays, the membrane lysates from HEK293T cells transfected with WT hABHD12 were labeled with FP-rhodamine (2 μm, 1 h, 37 °C), denatured using detergents and heating, and subsequently treated with PNGase F (0.01 unit/μg proteome) (or DPBS), and in-gel fluorescence was used as a readout for WT hABHD12. This experiment was done in biological triplicate with reproducible results. *C*, deglycosylation by PNGase F results in loss of lipase activity of hABHD12. Recombinant WT hABHD12 was treated with detergent (0.1% (w/v) Nonidet P-40) or detergent + PNGase F (0.1% (w/v) Nonidet P-40 + 0.1 unit/μg proteome) and assayed against C18:1 lyso-PS and C18:1 1-MAG substrates (100 μm each substrate). PNGase F-treated WT hABHD12 has almost no lipase activity against both substrates, comparable with mock and S246A hABHD12 membrane lysate controls. The data represent means ± S.D. for three biological replicates per group.

Because ABHD12 is an integral membrane enzyme and no purification method is described to date for hABHD12, we decided to perform all assays against membrane lysates derived from mock- or hABHD12-transfected HEK293T cells using a previously described transfection protocol (15). We also generated the “catalytically dead” active-site mutant (S246A) for hABHD12, to use as an additional control to mock-transfected lysates for these substrate assays (5). We confirmed hABHD12 activity in the membrane lysates obtained from WT hABHD12 transfected HEK293T cells and the lack thereof in the membrane lysates obtained from mock or the S246A hABHD12 transfected cells by gel-based activity–based protein profiling (ABPP) (16, 17) using the fluorophosphonate (FP)–rhodamine probe (6, 16) (Fig. 3*A*). We also confirmed by Western blotting analysis the near equal expression of WT and S246A hABHD12 in their respective membrane lysates and the lack of hABHD12 expression in mock membrane lysates (Fig. 3*A*). Previous studies have shown that mammalian ABHD12 is a highly glycosylated enzyme (10, 15), and we wanted to assess whether the glycosylation had any effect on the activity of hABHD12. Toward this, we first assessed whether WT hABHD12 expressed by us was glycosylated. We pretreated HEK293T membrane lysates overexpressing WT hABHD12 with FP-rhodamine and denatured these lysates by detergent treatment and heating, and the denatured lysates were treated with PNGase F as per the manufacturer's instructions. The resulting lysates were visualized by gel-based ABPP and Western blotting analysis for deglycosylation. We found from both these assays that overexpressed WT hABHD12 was indeed glycosylated, as evident from the shift of untreated band to a lower molecular weight band in PNGase F–treated samples (Fig. 3*B*). Next, we wanted to assess whether glycosylation of hABHD12 had any effect on its activity. However, we could not assess this by gel-based ABPP, because the PNGase F treatment is detergent-dependent, and addition of detergent to the gel-based ABPP assay protocol prevented any active FP-rhodamine labeling of WT hABHD12 in our experiments. We thus tested this in an established LC–MS lipase using C18:1 lyso-PS and C18:1 1-MAG lipid substrates (10, 15). We found from this assay that deglycosylation of WT hABHD12 results in complete loss of its lipase activity against both C18:1 lyso-PS and C18:1 1-MAG, suggesting that glycosylation of hABHD12 is critical for its lipase activity (Fig. 3*C*). Because we observed that detergent treatment from this protocol causes some loss of ABHD12 activity (∼25%), we avoided detergent in all subsequent ABHD12 activity assays.

### Substrate profiling study against recombinant hABHD12

**Figure 4.**
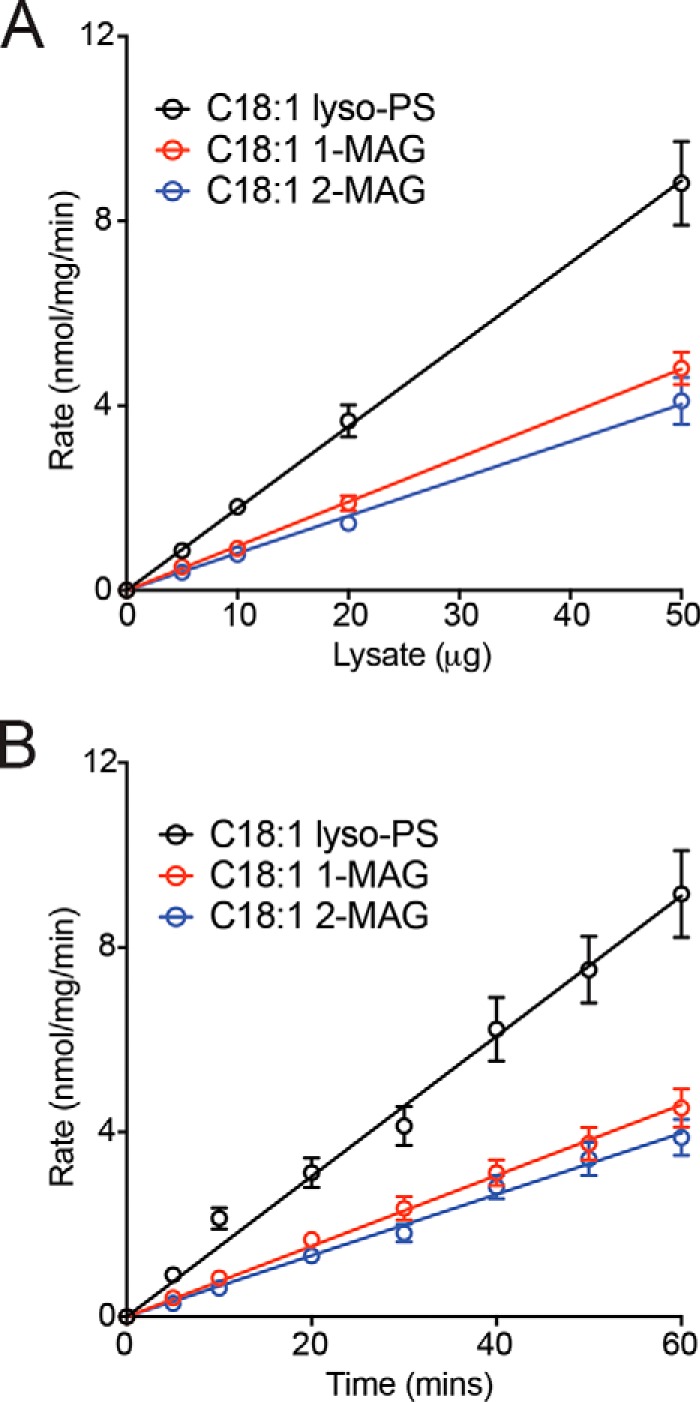
**Optimization of WT hABHD12 lipase activity assay.** *A*, the lipase activity of WT hABHD12 transfected HEK293T membrane lysates vary linearly with lysate concentrations (0–50 μg). *B*, the lipase activity of WT hABHD12 transfected HEK293T membrane lysate (20 μg) varies linearly with time of assay (0–1 h). All assays were done against three substrates, C18:1 lyso-PS, C18:1 1-MAG, and C18:1 2-MAG, at 100 μm concentration of each substrate as per the lipase assay protocol described under “Experimental procedures.” The data represent means ± S.D. for four biological replicates per group.

Having synthesized 16 1-MAG lipid substrates of varying lipid chain lengths and different unsaturation(s), we decided to perform enzyme kinetics studies on recombinant hABHD12 against this lipid substrate library, because such study is lacking for any mammalian ABHD12. Toward this, we first wanted to assess the relationship between enzyme concentrations and enzymatic rate. In this study we used C18:1 lyso-PS, C18:1 1-MAG, and C18:1 2-MAG as substrates (all 100 μm) and assayed them against varying concentrations of WT hABHD12 transfected HEK293T membrane lysates. We found a nice linear correlation between the enzyme concentration and all three substrates (Fig. 4*A*). Based on this study we chose 20 μg of membrane lysate for all subsequent studies, because this gave us a good enzymatic rate in our assays. Next, we assessed the relation between the enzymatic rate and the time of the assay. Like the previous study we used C18:1 lyso-PS, C18:1 1-MAG, and C18:1 2-MAG as substrates (all 100 μm) and assayed them against 20 μg of WT hABHD12 transfected HEK293T membrane lysate and measured lipase activity for these substrates as a function of time. We found that there was a nice linear relationship between the enzymatic rate and time of the assay up to 1 h, allowing initial velocity measurements for enzyme kinetics studies up to 1 h of this lipase assay (Fig. 4*B*). We chose 30 min for all subsequent lipase assays, because this allowed us enough time for any downstream sample processing and running multiple reactions at once. Because we were planning to assay different fatty acid containing 1-MAG lipids, we wanted to confirm that there was no difference in quantitatively measuring these free fatty acids in our LC–MS method. Indeed this was the case, where all free fatty acids from C10 to C24 behaved similarly and had a linear dynamic range from 1 pmol to 1 nmol in our LC–MS method (Table S1).

**Figure 5.**
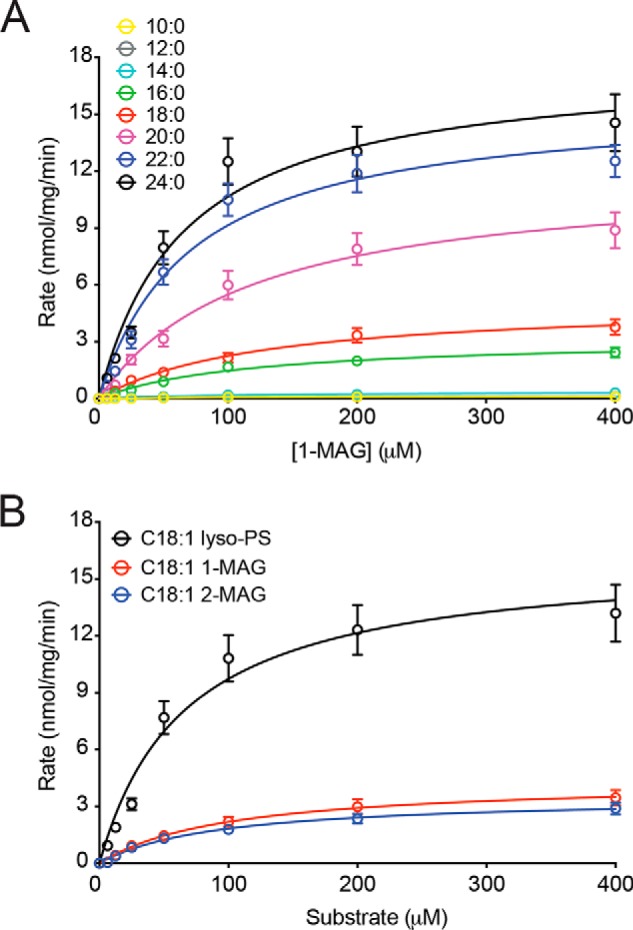
**Enzyme kinetic studies with recombinant hABHD12.** Enzyme kinetic assays of hABHD12 with saturated 1-MAG lipids of varying chain length (*A*) and C18:1 containing lyso-PS, 1-MAG, and 2-MAG substrates (*B*). The data presented in *A* show that hABHD12 has a strong preference for VLC 1-MAG lipids, whereas the data presented in *B* show that the substrate preference of hABHD12 is lyso-PS > 1-MAG > 2-MAG. The assays were performed with membrane lysates from mock or WT hABHD12 transfected HEK293T cells. The mock membrane lysate background enzymatic rate was subtracted from the WT hABHD12 membrane lysate rate to yield a corrected value for hABHD12 enzymatic rate, which is represented in this enzyme kinetics plot. Each concentration represents rates calculated from three biological replicates. The data are plotted as means ± S.D. for each concentration, and the *lines* connecting the points represent a fit to a classical Michaelis–Menten enzyme kinetics equation.

Having established the appropriate assay conditions for the enzyme kinetic studies, we tested both mock and WT hABHD12 against the 1-MAG substrate library at varying substrate concentrations (0–400 μm). The corrected enzymatic rate for WT hABHD12 for a particular concentration for a lipid was obtained by subtracting the corresponding mock rate at that concentration for that lipid, and the corrected WT hABHD12 enzymatic rates at different substrate concentrations for a particular lipid were plotted and fit to a classical Michaelis–Menten kinetics equation. The kinetic constants from these enzymatic assays for WT hABHD12 are reported in Table 1. Based on the enzyme kinetics data from the 1-MAG substrate profiling, *in vitro*, ABHD12 has a strong preference for VLC-containing 1-MAG lipids from all the kinetic constants, *i.e. V*_max_, *K_m_*, and *V*_max_/*K_m_* (Table 1). Comparing the kinetic constants for the saturated fatty acid 1-MAG lipids, we find a C24:0 ≈ C22:0 > C20:0 > C18:0 > C16:0 > C14:0 > C12:0 > C10:0 trend for *V*_max_ and *V*_max_/*K_m_* and a C24:0 < C22:0 < C20:0 < C18:0 < C16:0 ≈ C14:0 ≈ C12:0 < C10:0 trend for *K_m_* (Fig. 5*A*). Next, when comparing for 1-MAG lipids for a particular fatty acid chain length, we do not find any significant change in kinetic constants with increasing degree of unsaturation (*e.g.* for the C20:0, C20:1, and C20:4 group or the C22:0, C22:1, C22:4, and C22:6 group) (Table 1). To complement our 1-MAG substrate profiling studies, we purchased three commercially available 2-MAG lipid substrates with increasing fatty acid chain length and assayed them against recombinant hABHD12. Consistent with the enzyme kinetics data from the 1-MAG substrate profiling, we find for 2-MAG lipids that hABHD12 prefers C20:4 > C18:1 > C16:0 from all the kinetic constants for these lipids (Table 1). We also purchased three commercially available lyso-PS lipids and assayed them against hABHD12. Because only C16 and C18 fatty acid chain length lyso-PS lipids are readily available commercially, we find that hABHD12 prefers C18:0 and C18:1 lyso-PS lipids over C16:0 lyso-PS as substrates, and there is not much difference in the kinetic constants C18:0 and C18:1 lyso-PS (Table 1). Based on all the kinetic constants from our lipid substrate profiling, we find that hABHD12 has lyso-PS > 1-MAG > 2-MAG lipid substrate preference (Fig. 5*B*), which is consistent with literature precedence, and within the 1-MAG lipid substrates, we find that hABHD12 has a very strong preference for VLC-containing 1-MAG lipids (Fig. 5*A* and Table 1). We would like to note that we are not clear what physical form of the substrate (micellar or free lipid or both) hABHD12 can hydrolyze, and the kinetic constants reported in Table 1 are presented as a function of the total substrate concentration and are independent of these physical forms for the substrates. Finally, we tested the membrane lysates from S246A hABHD12 against the 1-MAG lipids at 100 μm and found no appreciable activity for the S246A hABHD12 against any 1-MAG lipid substrate compared with WT ABHD12 (Fig. S1).

**Table 1:** **Kinetic constants for the various lipid substrates tested in vitro against recombinant hABHD12**

Lipid species	*V*_max_	*K_m_*	*V*_max_/*K_m_*
	*nmol/mg protein/min*	μ*m*	*nmol/mg protein/min/m*
**1-MAG species**			
C10:0	0.14 ± 0.02	144 ± 14	(9.7 ± 0.9) × 10^2^
C12:0	0.16 ± 0.03	129 ± 21	(1.2 ± 0.3) × 10^3^
C14:0	0.38 ± 0.04	119 ± 22	(3.4 ± 0.4) × 10^3^
C16:0	3.2 ± 0.2	117 ± 21	(2.7 ± 0.4) × 10^4^
C18:0	5.1 ± 0.3	103 ± 19	(5.0 ± 0.5) × 10^4^
C18:1	5.6 ± 0.4	106 ± 15	(5.1 ± 0.6) × 10^4^
C18:3	5.4 ± 0.3	109 ± 15	(4.9 ± 0.5) × 10^4^
C20:0	12.0 ± 0.8	91 ± 11	(1.3 ± 0.2) × 10^5^
C20:1	12.3 ± 0.9	86 ± 14	(1.4 ± 0.3) × 10^5^
C20:4	12.6 ± 0.9	91 ± 12	(1.4 ± 0.4) × 10^5^
C22:0	15.7 ± 1.3	72 ± 12	(2.2 ± 0.3) × 10^5^
C22:1	15.6 ± 1.2	75 ± 13	(2.0 ± 0.4) × 10^5^
C22:4	15.1 ± 0.8	78 ± 8	(1.9 ± 0.3) × 10^5^
C22:6	14.9 ± 1.4	79 ± 9	(1.8 ± 0.3) × 10^5^
C24:0	17.7 ± 1.5	66 ± 9	(2.7 ± 0.3) × 10^5^
C24:1	17.9 ± 1.8	61 ± 8	(2.9 ± 0.4) × 10^5^

**2-MAG species**			
C16:0	2.3 ± 0.4	148 ± 31	(1.5 ± 0.3) × 10^4^
C18:1	3.6 ± 0.5	129 ± 24	(2.8 ± 0.3) × 10^4^
C20:4	6.8 ± 0.5	95 ± 17	(7.2 ± 0.8) × 10^4^

**Lyso-PS species**			
C16:0	7.5 ± 0.7	87 ± 14	(8.6 ± 0.8) × 10^4^
C18:0	14.8 ± 1.4	73 ± 12	(2.0 ± 0.3) × 10^5^
C18:1	14.3 ± 0.8	74 ± 11	(1.9 ± 0.3) × 10^5^

### Substrate profiling study against endogenous ABHD12 from mouse brain

**Figure 6.**
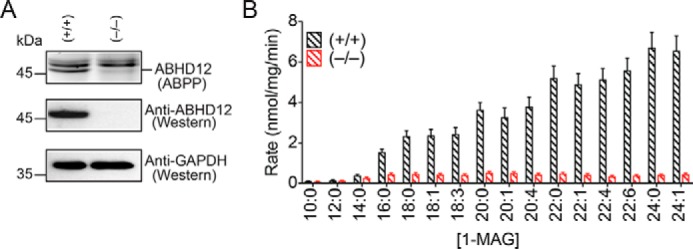
**Enzymatic assays of 1-MAG lipids with endogenous mouse brain ABHD12.** *A*, gel-based ABPP and Western blotting analysis were used to confirm the loss of ABHD12 activity and expression in the membrane lysates of brain membrane preparations from WT (+/+) and ABHD12 knockout (−/−) mice, respectively. As a loading control, GAPDH was used. This experiment was done in biological triplicates with reproducible results. *B*, brain membrane lysates from WT (+/+) and ABHD12 knockout (−/−) mice were treated with 1 μm each of JZL184 and KT195 and were assayed against the 1-MAG lipid library (100 μm of each 1-MAG substrate). The data represent means ± S.D. for three biological replicates per group.

To complement the enzyme kinetic studies that used recombinantly expressed hABHD12, we wanted to determine the 1-MAG lipid substrate preference of endogenous mammalian ABHD12. Toward this, we chose the mouse brain membrane lysates as a model system, because previous studies have shown that it expresses high levels of active ABHD12 (5, 10, 18, 19). In this study, we used WT and ABHD12 knockout mice that were littermates (5). In accordance with previous studies, we confirmed by gel-based ABPP that our brain membrane lysate preparations ABHD12 knockout mice were indeed devoid of any ABHD12 activity, whereas WT brain membrane preparations had robust ABHD12 activity (Fig. 6*A*). Additionally, we confirmed by Western blotting analysis that ABHD12 expression was indeed absent in ABHD12 knockout mouse brain membrane lysates (Fig. 6*A*). Having confirmed the lack of ABHD12 expression and activity in ABHD12 knockout brain membrane lysates, we decided to use this as an assay control, to measure the ABHD12-specific 1-MAG lipase activity of WT mouse brain membrane lysates.

Previous studies have also shown that the mouse brain membrane lysate possess three enzymes, namely monoacylglycerol lipase (MAGL), ABHD12 and ABHD6, which can hydrolyze MAG substrates, hence complicating the specific contribution that ABHD12 has toward 1-MAG lipase activity in the mouse brain membrane lysate (10–12). Fortunately, there are very selective and potent inhibitors available for both MAGL (JZL184: MAGL inhibitor) (20) and ABHD6 (KT195: ABHD6 inhibitor) (21), which can be used in tandem in mouse brain membrane lysates to assess the specific 1-MAG lipase activity of ABHD12 in this endogenous system. We therefore treated WT and ABHD12 knockout brain membrane lysates with both JZL184 and KT195 (37 °C, 1 h, 1 μm each) (Fig. S2) and assayed these inhibitor-treated brain membrane lysates against the 1-MAG lipid substrate library. Concomitant with the 1-MAG substrate profile of the recombinantly expressed human ABHD12, we find that the endogenous mouse brain ABHD12 also displays the best catalytic activity for VLC-containing 1-MAG lipids in the entire panel of 1-MAG substrates (Fig. 6*B*).

### Cellular localization of ABHD12

**Figure 7.**
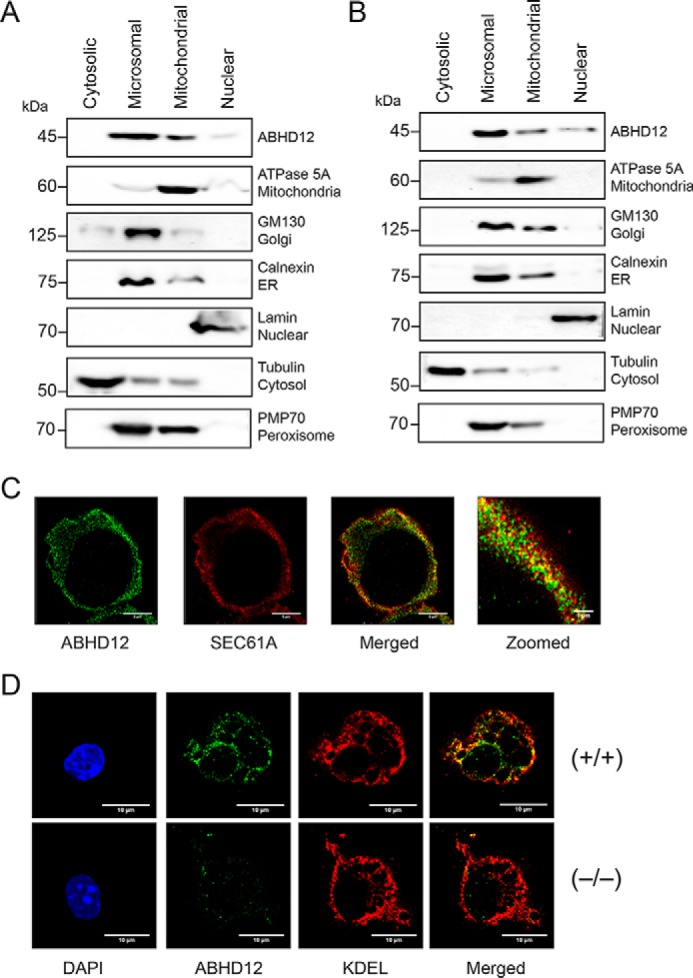
**Cellular organelle fractionation and immunofluorescence confirm the ER localization of mammalian ABHD12.** *A* and *B*, cellular organelle fraction studies showing the microsomal enrichment of mammalian ABHD12 in the mouse brain (*A*) and Neuro-2a cells (*B*). All the cellular fractionation experiments were done in at least biological triplicates with reproducible results. *C*, high-resolution cellular immunofluorescence studies in Neuro-2a cells showing that ABHD12 has strong colocalization with SEC61A, an ER marker (*yellow* in merged image). *D*, cellular immunofluorescence studies in primary peritoneal macrophages from WT (+/+) and ABHD12 knockout (−/−) mice, showing that ABHD12 has strong colocalization with KDEL, an ER marker, in the WT mice (*yellow* in merged images in (+/+) panels) and the lack of ABHD12 expression in the knockout mice (no *green* fluorescence in (−/−) panels). All the cellular immunofluorescence experiments were done in at least four biological replicates with reproducible results.

Having established a substrate profile for both recombinantly expressed and endogenous ABHD12, we wanted to determine whether there exists any correlation between the VLC fatty acid preference for this enzyme and its cellular localization. To address this, we used two approaches. First, we fractionated the different cellular components using established cellular organelle fractionation techniques and assessed the ABHD12 localization by Western blotting analysis. This cellular fractionation method is relatively inexpensive and typically affords cellular organelle fractions that are ≥90% enriched, and hence provides a tentative idea of the enrichment of a particular protein in a particular fraction before venturing into more sophisticated techniques like microscopy (22). We performed this fractionation study on WT mouse brains and in two mammalian cell lines (Neuro-2a and MCF7), because ABHD12 expression and activity has previously been confirmed in these mammalian cells (15, 21). In mouse brains, we found that ABHD12 was significantly enriched (>85%) in the microsomal fraction, which is composed primarily of the endoplasmic reticulum (ER) (anti-calnexin) (Fig. 7*A*). Corroborating the cellular fractionation studies from the mouse brains, we find that in both Neuro-2a (Fig. 7*B*) and MCF7 cells (Fig. S3), ABHD12 is significantly enriched in the microsomal fraction (> 90%).

Second, to complement these organelle fraction studies and confirm the cellular localization of mammalian ABHD12, we performed cellular immunofluorescence assays in conjunction with high-resolution microscopy in Neuro-2a cells (Fig. 7*C*). We found from these studies that ABHD12 had significant overlap with the ER marker (anti-SEC61A) and hence had significant ER localization (Fig. 7*C*). The microscopy-based cellular immunofluorescence studies also showed that ABHD12 had virtually no localization in the nucleus, Golgi, mitochondria, or with the actin cytoskeleton in Neuro-2a cells (Fig. S4). Similar results were also seen in MCF7 cells (Figs. S5 and S6). Finally, we also performed similar cellular immunofluorescence studies in primary peritoneal macrophages derived from WT or ABHD12 knockout mice and found that ABHD12 was indeed localized to the ER membrane in primary mouse peritoneal macrophages (Fig. 7*D*).

## Discussion

The integral membrane serine hydrolase enzyme ABHD12 is expressed highly in the central nervous and immune system (5, 15, 18, 19), and mutations to this enzyme in humans result in the neurological disorder PHARC (1). Recently the murine model of PHARC, *i.e.* the ABHD12 knockout mouse was generated, characterized, and shown to have massive accumulation of VLC-containing lyso-PS lipids in the brain, which were correlated to the age-dependent PHARC like phenotypes displayed by these mice (5). Here, using a classical biochemical approach, broadly encompassing synthetic organic chemistry to make a substrate library, rigorous enzyme kinetics studies, coupled with cellular fractionation and immunofluorescence, we set out to provide an explanation as to why the VLC-containing lyso-PS lipids are massively elevated in the brains of these mice.

Previous *in vitro* studies have shown that ABHD12 accepts 1-MAG lipids as substrates with catalytic efficiencies quite comparable with lyso-PS lipids, the putative endogenous substrates of ABHD12 (Fig. 1) (11, 12). First, we developed a facile synthetic scheme for the generation of a library of 1-MAG lipids from well-known reactions, and this synthetic route afforded the generation of 1-MAG lipids of varying chain length and unsaturation with fairly good yields, even at a low-milligram synthetic scale (Fig. 2). We leveraged this synthesis to generate VLC 1-MAG lipids, which have to date not been assayed against mammalian ABHD12. We overexpressed hABHD12 in HEK293T cells and show that glycosylation of hABHD12 is critical for its activity (Fig. 3). We also report the optimization of our LC–MS–based lipase assay for enzyme kinetic measurements and found a nice linear correlation between enzyme activity with enzyme concentration and assay time (Fig. 4). Next, we tested this 1-MAG lipid library consisting of fatty acids ranging from C10 to C24 against recombinant hABHD12 and found from the enzyme kinetics study that hABHD12 has a strong preference for VLC 1-MAG lipids as substrates (Fig. 5 and Table 1). Based on the kinetic constants (Table 1), it appears that the VLC substrate specificity for hABHD12 (*V*_max_/*K_m_*) is likely due to the increased rate of the reaction (*V*_max_) rather than the decreased apparent affinity (*K_m_*). We thus speculate that this might be another example of an enzyme showing increased specificity caused by improved catalysis and not improved substrate binding. Further, we find that hABHD12 does not have any preference for unsaturated 1-MAG lipids for a particular chain length, suggesting that the lipid substrate preference lies predominantly for the fatty acid chain length and not the extent of unsaturation of the 1-MAG substrate (Table 1). We also tested the endogenous mouse brain ABHD12 against the 1-MAG lipid library and found that endogenous mouse brain ABHD12 also exhibits a similar substrate profile as that of recombinant hABHD12, in that VLC 1-MAG lipids are the most preferred substrates for ABHD12 (Fig. 6). We would also like to add that because we are performing these substrate assays with membrane lysates containing the overexpressed enzyme and not the purified enzyme, we cannot rule out the possibility that the apparent substrate specificity of hABHD12 might be due to the increased solubility of the VLC substrates in the membrane, which might make these substrates more accessible to the enzyme. Finally, we show by both cellular organelle fractionation and cellular immunofluorescence that mammalian ABHD12 has a predominant ER membrane localization in different mammalian cells (including primary peritoneal macrophages) and the mouse brain (Fig. 7).

In mammals, VLC fatty acids (≥C22) are biosynthesized predominantly by an enzyme named fatty acid elongase (23). This enzyme in humans, is encoded by the ELOVL genes, has seven isoforms and is a membrane-bound ER-resident enzyme (23). As a consequence, significant cellular biosynthesis of VLC fatty acids occurs in the ER (23, 24). In humans, VLC fatty acids serve several functions, like maintenance of myelin sheath, normal neuronal function, liver homeostasis, retinal functions, skin barrier formation, anti-inflammatory properties, and spermatogenesis, among others (24–26). Not surprisingly, mutations in genes that are involved in VLC fatty acid biosynthesis or metabolism cause several inherited disorders in humans. Before genetic mapping, PHARC was misdiagnosed as Refsum's disease (or syndrome), another autosomal recessive genetic disease, because human subjects with the latter also clinically present with peripheral neuropathy, retinitis pigmentosa, hearing loss, early onset of cataract, and cerebellar ataxia (2). Of note, Refsum's disease is caused by mutations to the *PHYH* gene, which is responsible for the metabolism of a dietary branched chain fatty acid, phytanic acid, through its α-oxidation in the peroxisomes. The defective metabolism and resulting accumulation of phytanic acid in the blood and nervous system tissues caused by the faulty *PHYH* gene function causes the aforementioned symptoms. Interestingly, the symptoms of both Refsum's disease and PHARC are very similar, and studies from the murine model of PHARC suggest that the PHARC-like phenotypes might also be attributed to defective lipid metabolism, in the central nervous system (lyso-PS in case of PHARC). Here, we show that ABHD12 is localized at the cellular compartment (ER membrane) where the biosynthesis of both VLC fatty acids and phosphatidylserine lipids is the highest (24, 27). Our biochemical studies, which include enzyme kinetics and cellular localization data, along with the studies from others (5) suggest speculatively that given ABHD12's preference for VLC lipids, it functions as a major lipase controlling the concentrations and flux of the VLC lipids (lyso-PS), which are constantly biosynthesized in the ER membrane, and perturbation in the activity of ABHD12 causes unchecked accumulation of these VLC lipids particularly lyso-PS lipids, resulting in the pathophysiology observed in PHARC subjects.

Projecting forward, it would be beneficial to develop a synthetic route toward making lyso-PS lipids of varying chain lengths, so that our current biochemical study can be validated against the putative endogenous substrate of ABHD12. Additionally, the new synthetic route might afford the generation of photoreactive bioorthogonal lyso-PS probes, which in conjunction with recently established MS-based chemoproteomics (28–30) would greatly facilitate the discovery of protein ligands and/or receptors of the possibly pathological VLC lyso-PS lipids that are as of yet unknown. Although it is now known that much of the VLC PS biosynthesis occurs in the ER (24, 27), the biosynthetic origins of VLC lyso-PS lipids remain elusive. There are currently two candidate enzymes namely ABHD16A (15) and PS-PLA1 (31, 32) that are responsible for the biosynthesis of lyso-PS lipids from PS precursors. Understanding the substrate scope, especially the VLC PS preference and mapping the cellular localization of these enzymes, would greatly facilitate the understanding of the biosynthesis of VLC lyso-PS lipids and its spatiotemporal localization in mammalian cells.

## Experimental procedures

### Materials

All chemicals, buffers, solvents, and reagents were purchased from Sigma–Aldrich (now Merck). All lipids and lipid standards were purchased from Avanti Polar Lipids Inc., and all primary and secondary antibodies were purchased from Abcam, unless otherwise mentioned.

### Synthesis of 1-MAG lipids

#### 

##### For reaction 1

To a solution of the desired free fatty acid (1.0 equivalent) and 1,2-isopropylideneglycerol (1.0 equivalent) in anhydrous dichloromethane (CH_2_Cl_2_, 1.5 ml) maintained at 0 °C, *N*,*N*-dimethyl-4-aminopyridine (0.25 equivalent) and EDC·HCl (1.0 equivalent) was sequentially added. The reaction mixture was warmed to room temperature and stirred for 16 h. Upon disappearance of starting compound observed on TLC, the reaction was quenched with saturated sodium bicarbonate (NaHCO_3_) and extracted three times with CH_2_Cl_2_. The combined organic layer was dried over sodium sulfate (Na_2_SO_4_) and filtered, and the filtrate was concentrated. The crude residue was purified by column chromatography using 5% ethyl acetate/hexane as an eluent to afford the corresponding fatty acid ester.

##### For reaction 2

Amberlyst-15 (H^+^ form, 0.5 equivalent) was added to a solution of fatty acid ester (1.0 equivalent) in MeOH. The resulting reaction mixture was stirred for 16 h at room temperature. After completion of reaction (TLC analysis), Amberlyst-15 was filtered off, and the filtrate was evaporated under reduced pressure. The crude residue was purified by column chromatography using 40% ethyl acetate/hexane as an eluent to afford the corresponding 1-MAG lipid. The detailed synthesis and compound characterization data of each individual 1-MAG lipid can be found in the supporting information.

##### General material and methods for the synthesis

Merck silica gel TLC plates (0.25 mm, 60 F254) were used to monitor all chemical reactions. Column chromatography was performed using silica gel Rankem (60–120 mesh) or silica gel Spectrochem (100–200 mesh). Unless otherwise specified, ^1^H and ^13^C spectra were recorded on a JEOL 400 MHz (or 100 MHz for ^13^C) or a Bruker 400 MHz (or 100 MHz for ^13^C) spectrometer using either residual solvent signals (CDCl_3_ δ_H_ = 7.26 ppm, δ_C_ = 77.2 ppm) or as an internal tetramethylsilane (δ_H_ = 0.00, δ_C_ = 0.0). Chemical shifts (δ) are reported in ppm and coupling constants (*J*) in Hz. The following abbreviations are used: br (broad signal), m (multiplet), s (singlet), d (doublet), t (triplet), and dd (doublet of doublets). High-resolution mass spectra were obtained from high-resolution mass spectrometry (HRMS)–electrospray ionization–Q-TOF–LC–MS/MS (Sciex) available at Indian Institute of Science Education and Research Pune Mass Spectrometry Core.

### Expression of hABHD12 in HEK293T cells and preparation of membrane lysates

The full-length WT hABHD12 cDNA was purchased from GE Life Sciences Dharmacon, cloned in pCMV-Sport6 vector between the NotI and SalI restriction sites. The catalytically inactive S246A hABHD12 mutant (5) was generated using DpnI-based site-directed mutagenesis using Phusion polymerase (New England Biolabs) as per the manufacturer's instructions. HEK293T cells were purchased from ATCC and cultured in complete DMEM that contained DMEM (HiMedia) supplemented with 10% (v/v) fetal bovine serum (Thermo Fisher Scientific), and 1× penicillin streptomycin (MP Biomedicals) at 37 °C with 5% (v/v) CO_2_. The cells were periodically stained with 4′,6-diamidino-2-phenylindole to ensure they were devoid of any mycoplasma contamination. The hABHD12 was expressed recombinantly in HEK293T cells using a previously described transfection protocol (15). Briefly, HEK293T cells were grown to 35% confluence in complete DMEM (15-cm dish) at 37 °C with 5% (v/v) CO_2_, and the cells were transiently transfected with the above-mentioned vectors (WT- and S246A-ABHD12) using polyethyleneimine “max” 40,000 MW (Polysciences, Inc.) (1:3 = plasmid:polyethyleneimine, 15 μg of plasmid for a 15-cm dish). Mock control cells were transfected with an empty vector using the same protocol (15). The cells were harvested by scraping 48 h after transfection, washed with sterile Dulbecco's phosphate-buffered saline without calcium and magnesium (pH 7.2) (DPBS) (Thermo Fisher Scientific) (three times), resuspended 1 ml of DPBS, and lysed by sonication. The cellular debris was pelleted by centrifugation at 200 × *g* for 5 min at 4 °C, and the resulting lysate (∼900 μl) was separated and centrifuged at 100,000 × *g* for 45 min at 4 °C. The supernatant was discarded, and the pellet (membrane proteome) was washed with cold sterile DPBS (three times) and resuspended in 500 μl of cold sterile DPBS by pipetting. The protein concentration was estimated using a BCA protein assay kit (Pierce), and the expression and activity (lack of it in S246A hABHD12) of hABHD12 was confirmed by Western blotting analysis and gel-based ABPP, respectively (16). For the gel-based ABPP assays, the membrane proteomes (1 mg/ml, 100 μl) were treated with 2 μm FP-rhodamine for 45 min at 37 °C with constant shaking. The addition of 4× SDS loading buffer followed by boiling the samples at 95 °C for 10 min, which quenched these reactions. Fluorescently labeled proteomes were resolved on a 10% SDS–PAGE gel, and the samples were visualized for enzyme activity by in-gel fluorescence using a Syngene G-Box Chemi-XRQ gel documentation system.

### Preparation of brain membrane lysates

All mouse studies were performed following protocols that received approval from the Indian Institute of Science Education and Research Pune Institutional Animal Ethics Committee. The mice were genotyped using an established protocol (5). The mouse brain membrane proteomes were prepared using a previously described procedure (15). Briefly, the mice were first anesthetized with isoflurane and euthanized by cervical dislocation, following which the brains of the mice were harvested. A fresh half brain was suspended in 500 μl of cold sterile DPBS and homogenized using a tissue homogenizer (Bullet Blender 24, Next Advance) with one scoop of glass beads (0.5-mm diameter; Next Advance) at a speed setting of 8 for 3 min at 4 °C. To the brain homogenate, an additional 500 μl of cold sterile DPBS was added, mixed by pipetting, and centrifuged at 1000 × *g* for 5 min at 4 °C to separate the tissue debris. The resulting supernatant (∼700 μl) was separated and centrifuged at 100,000 × *g* for 45 min at 4 °C. The resulting supernatant was discarded, and the pellet (membrane proteome) was washed with cold sterile DPBS (three times) and resuspended in 1 ml of cold sterile DPBS by pipetting. The protein concentration of the brain membrane proteome was estimated using BCA protein assay kit (Pierce).

### PNGase F treatments

For the gel-based ABPP assays, the PNGase F treatments were done under denaturing conditions as per the manufacturer's instructions (Sigma–Aldrich). Briefly, 50 μg of HEK293T transfected ABHD12 membrane lysates were treated with FP-rhodamine (4 μm, 1 h, 37 °C, 650 rpm shaking). To this 0.5× of glycoprotein denaturing buffer containing 5% (w/v) SDS and 400 mm DTT was added and denatured by boiling at 95 °C for 10 min. The samples were than cooled instantly on ice for 5 min, and 0.1% (w/v) Nonidet P-40 was added. To this denatured labeled proteome, PNGase F at 0.01 unit/μg proteome was added and incubated at 37 °C for 1 h with constant shaking (750 rpm). The reaction was quenched with 4× loading buffer, and proteome was resolved on 12% SDS–PAGE gel. In-gel fluorescence of labeled protein was visualized using a Syngene G-Box Chemi-XRQ gel documentation system, and Western blotting was performed for the same gel. For LC–MS–based lipase assays, the PNGase F treatments were done under nondenaturing conditions. Briefly, the membrane proteome was treated with PNGase F at 0.1 unit/μg proteome and 0.1% (w/v) Nonidet P-40 and incubated at 37 °C for 1 h with constant shaking (750 rpm). 20 μg of this membrane proteome was used for lipase assays as described below. All of the substrates were assayed at 100 μm in this assay.

### Lipid substrate assays

All of the lipase assays were performed with 20 μg of membrane proteome in a final volume of 100 μl. The enzyme kinetics assays were performed with varying substrate concentrations (0- 400 μm), whereas all single concentration assays were performed at 100 μm of the respective lipid substrate. The lipase assays were performed using a previously established protocol in glass vials (15, 33, 34). Briefly, 20 μg of the membrane proteome was incubated with lipid substrate of desired concentration in DPBS (100 μl total volume) at 37 °C with constant shaking. After 30 min, the reaction was quenched by addition of 250 μl of 2:1 (v/v) chloroform (CHCl_3_):methanol (MeOH) containing 1.25 nmol of the internal standard (heptadecenoic acid, C17:1 free fatty acid). The two phases were separated by centrifugation at 3000 × *g* for 5 min, and the organic phase (bottom) was removed. The organic extracts were dried under a stream of N_2_, and resolubilized in 100 μl of 2:1 (v/v) CHCl_3_:MeOH. The LC–MS analysis was done using a previously established protocol (15, 33, 34). Measuring the area under the curve and normalizing to the respective internal standard was used to quantify the free fatty acid release in this lipase assay. The nonenzymatic rate of substrate hydrolysis was obtained by using heat-denatured proteomes (15 min at 95 °C, followed by cooling at 4 °C for 10 min, three times) as a control, and this value was subtracted from the substrate hydrolysis rates of native proteomes to yield the corrected rates. For the enzyme kinetics assays, the mock membrane hydrolysis rate was subtracted from the WT hABHD12 membrane hydrolysis rate for a particular lipid substrate concentration to yield a corrected enzymatic rate for the WT hABHD12 membrane lysates.

### Western blotting analysis

Membrane lysates were resolved on a 10% SDS–PAGE gel and transferred onto a PVDF membrane (GE Healthcare) (60 V for 12 h at 4 °C). Post-transfer, the membrane was blocked with 5% (w/v) milk in TBS (pH 7.4) containing 0.1% (w/v) Tween 20 (TBST) and subsequently probed with a primary antibody (dilution 1:1000). Thereafter the membrane was washed with TBST (three times) and incubated with the appropriate secondary antibody for 90 min at 25 °C. The membrane was subsequently washed with TBST (three times), and the signal was visualized using the Thermo West Pico Western blotting substrate (Thermo Fisher Scientific) using a Syngene G-Box Chemi-XRQ gel documentation system. The primary antibodies used in the study were anti-ABHD12 (rabbit, Abcam, 182011), anti-ATP5A (mouse, Abcam, ab14748), anti-GM130 (mouse, Abcam, ab169276), anti-calnexin (rabbit, Abcam, ab10286), anti-PMP70 (mouse, Sigma–Aldrich, SAB4200181), anti-lamin (rabbit, Cloud Clone Corp, CAF548Hu01), anti-tubulin (rabbit, Cloud Clone Corp, CAB870Hu01), and anti-GAPDH (mouse, Cloud Clone Corp, CAB932Hu01). The secondary antibodies used in this study were horseradish peroxidase–conjugated anti-rabbit IgG (goat, Thermo Fisher Scientific, 31460) and horseradish peroxidase–linked anti-mouse IgG (goat, Cloud Clone Corp, SAA544Mu19).

### Cellular fractionation studies

The cellular organelle fractionation from mouse brains and mammalian cell lines were performed using a previously described protocol (22). Briefly, mammalian cells from six 15-cm tissue culture plates (80% confluent) or half-mice brain was processed in a Dounce homogenizer in 7 ml of 250-STM homogenization buffer containing 250 mm sucrose, 50 mm Tris-HCl (pH 7.4), 5 mm MgCl_2_, 1 mm DTT, 25 μg/ml spermine, 25 μg/ml spermidine, and 1 mm phenylmethylsulfonyl fluoride until floating nuclei were seen under a phase contrast microscope. The unlysed cells were pelleted by centrifuging at 200 × *g* for 3 min at 4 °C, and the resulting supernatant was centrifuged at 1000 × *g* for 15 min at 4 °C to pellet the nuclear fraction. The supernatant from this step was further centrifuged at 10,000 × *g* for 15 min at 4 °C to pellet the crude mitochondrial fraction, and the leftover supernatant was centrifuged at 100,000 × *g* for 60 min at 4 °C. The resulting pellet was collected as microsomal fraction, and supernatant was collected as the cytosolic fraction. The crude mitochondrial fraction was additionally cleaned by resuspending this fraction in 10 volumes of 2 m-STMDPS buffer containing 2 m sucrose, 50 mm Tris-HCl (pH 7.4), 5 mm MgCl_2_, 1 mm DTT, 25 μg/ml spermine, 25 μg/ml spermidine, and 1 mm phenylmethylsulfonyl fluoride and centrifuging it at 6,000 × *g* for 15 min at 4 °C to pellet a pure mitochondrial fraction. Each pelleted fraction was resuspended in cold sterile DPBS, and protein was quantified using Bradford reagent (Sigma–Aldrich), following which the proteome was denatured by boiling with 4× loading buffer at 95 °C for 15 min. The denatured proteomes were resolved on a 10% SDS–PAGE gel and assessed by above described Western blotting analysis for enrichment of appropriate proteins in the respective cellular fraction.

### Cellular immunofluorescence studies

The cellular immunofluorescence studies were performed using manufacturer-recommended protocols (Thermo Fisher Scientific). The Neuro-2a and MCF7 cell lines were purchased from ATCC and cultured in DMEM (HiMedia) with 10% (v/v) fetal bovine serum (Thermo Fisher Scientific) and 1% penicillin-streptomycin (MP Biomedicals). The cells were counted using a trypan blue method on a BTC20 automated cell counter (Bio-Rad) as per the manufacturer's instructions, and a 35-mm tissue culture dish containing a 18-mm coverslip was seeded with 2.5 × 10^6^ cells for 24 h at 37 °C and 5% (v/v) CO_2_. The cells were washed with sterile DPBS and fixed with 4% (w/v) paraformaldehyde in DPBS for 20 min at 25 °C. Subsequently, the fixed cells were washed with DPBS (three times) and permeabilized with 0.5% (v/v) Triton X-100 in DPBS containing 5% (w/v) BSA for 15 min at 25 °C. Thereafter, the permeabilized cells were blocked with 5% (w/v) BSA in DPBS (blocking buffer) for 30 min at 25 °C and probed with primary antibody (1:100 dilution) for 90 min at 25 °C, following which the secondary antibody treatments (1:1000 dilution) were done for 60 min at 25 °C. Lastly, the nuclei were stained with 4′,6-diamidino-2-phenylindole (nuclear marker; Sigma–Aldrich) for 5 min at 25 °C.

For confocal microscopy, the coverslip was then mounted on a glass slide with a drop of Fluoromount^TM^ aqueous mounting medium (Sigma–Aldrich). The mounting medium was allowed to dry in the dark at 25 °C, and the slides were imaged on a Zeiss LSM 710 confocal laser-scanning microscope fitted with a 63×, 1.4 oil immersion objective. For stimulated emission depletion microscopy, the coverslip was then mounted on a glass slide with a drop of Mowiol-DABCO mounting medium. The mounting medium was allowed to dry in the dark at 25 °C, and the slides were imaged on a Leica TCS stimulated emission depletion super-resolution microscope with HC PL APO CS2, 100×, 1.4 oil immersion objective. The raw images were deconvoluted using Huygens professional software (Scientific Volume Imaging BV), and all microscopy images were analyzed using ImageJ 1.50i software (National Institutes of Health for Windows OS). The primary antibodies used in these studies were anti-ABHD12 (rabbit, Abcam, ab87048 or mouse, Abcam, ab68949), anti-KDEL (mouse, Abcam, ab12223, ER marker), anti-ATP5A (mouse, Abcam, ab14748, mitochondrial marker), and anti-GM130 (mouse, BD Transduction Labs, 610822, Golgi marker). Alexa Fluor 488 phalloidin (Thermo Fisher Scientific, A12379) or Alexa Fluor 568 phalloidin (Thermo Fisher Scientific, A12380) was used to stain the actin cytoskeleton. Goat anti-rabbit IgG (H&L) conjugated with DyLight® 488 (Thermo Fisher Scientific, 35552), donkey anti-rabbit IgG (H&L) conjugated with Alexa Fluor 568 (Thermo Fisher Scientific, A-10042), goat anti-mouse IgG (H&L) conjugated with Alexa Fluor Plus 488 (Thermo Fisher Scientific, A-11017), and goat anti-mouse IgG (H&L) conjugated with Alexa Fluor 568 (Thermo Fisher Scientific, A-11004) were used as secondary antibodies.

### Data fitting and plotting

The enzyme kinetics data were fit to a classical Michaelis–Menten equation built in the Prism 7 software for Mac OS X (GraphPad). All data were plotted using the same software. All data are shown as means ± S.D. for three (or more) biological replicates.

## Author contributions

A. J., M. S., and A. R. investigation; A. J., M. S., S. S., A. R., and A.M. methodology; A. J., M. S., and S. S. writing-review and editing; S. S. and A. R. validation; A.M. and S. S. K. formal analysis; A.M. and S. S. K. supervision; S. S. K. conceptualization; S. S. K. funding acquisition; S. S. K. writing-original draft.

## Supplementary Material

Supporting Information

## Footnotes

3 The abbreviations used are:

PHARC: polyneuropathy, hearing loss, ataxia, retinitis pigmentosa, cataract
VLC: very long chain
MAG: mono-acyl-glycerol
1-MAG: mono-1-(fatty)acyl-glycerol
2-MAG: mono-2-(fatty)acyl-glycerol
lyso-PS: lysophosphatidylserine
2-AG: 2-arachidonoyl-glycerol
EDC: 1-ethyl-3-(3-dimethylaminopropyl)carbodiimide
ABPP: activity–based protein profiling
FP: fluorophosphonate
PNGase F: peptide:*N*-glycosidase F
h: human
MAGL: monoacylglycerol lipase
ER: endoplasmic reticulum
DMEM: Dulbecco's modified Eagle's medium
DPBS: Dulbecco's phosphate-buffered saline without calcium and magnesium (pH 7.2).
