# Supplementary material for: Biochemical characterization of the PHARC-associated serine hydrolase ABHD12 reveals its preference for very-long-chain lipids
Source: J Biol Chem. 2018 Sep 20;293(44):16953–63. doi: 10.1074/jbc.RA118.005640 (PMC6217928; doi:10.1074/jbc.RA118.005640)
Supplement: Supporting Information [file supp_293_44_16953__index.html]

Biochemical characterization of the PHARC associated serine hydrolase ABHD12 reveals its preference for very long chain lipids — ABHD12 prefers very long chain lipids — Biochemical characterization of the PHARC-associated serine hydrolase ABHD12 reveals its preference for very-long-chain lipids — ABHD12 prefers very-long-chain lipids — Supporting Information 

# Biochemical characterization of the PHARC-associated serine hydrolase ABHD12 reveals its preference for very-long-chain lipids

## Supporting Information

- Supporting Information - Supplementary Figures and Table
- Supporting Information: synthesis - Detailed compound synthesis and characterisation
