# Supplementary material for: Biochemical characterization of the PHARC-associated serine hydrolase ABHD12 reveals its preference for very-long-chain lipids
Source: J Biol Chem. 2018 Sep 20;293(44):16953–63. doi: 10.1074/jbc.RA118.005640 (PMC6217928; doi:10.1074/jbc.RA118.005640)
Supplement: Supporting Information [file supp_RA118.005640_140484_1_supp_203915_pf6z84.pdf]

Supplementary Figures

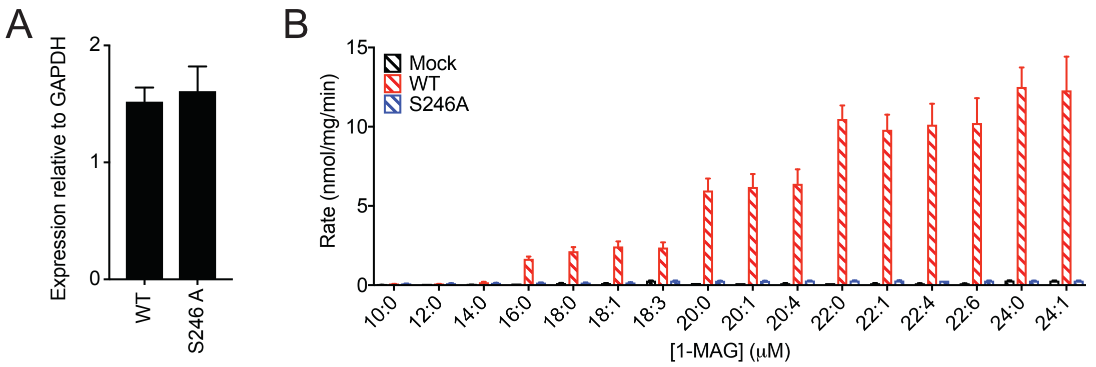

Supplementary Figure 1. (**A**) Quantitative western blot analysis showing comparable expression of WT hABHD12 and S246A hABHD12 relative to GAPDH in HEK293T membrane lysates transfected with WT hABHD12 or S246A hABHD12 respectively. Data represents mean ± standard deviation for four biological replicates per group. (**B**) Enzymatic lipase assays for membrane lysates from mock, or WT ABHD12 or S246A ABHD12 transfected HEK293T cells, tested against 100 μM of respective 1-MAG lipid. Data represents mean ± standard deviation for three biological replicates per group. The lipase assays show negligible activity for the S246A hABHD12 mutant relative to WT hABHD12.

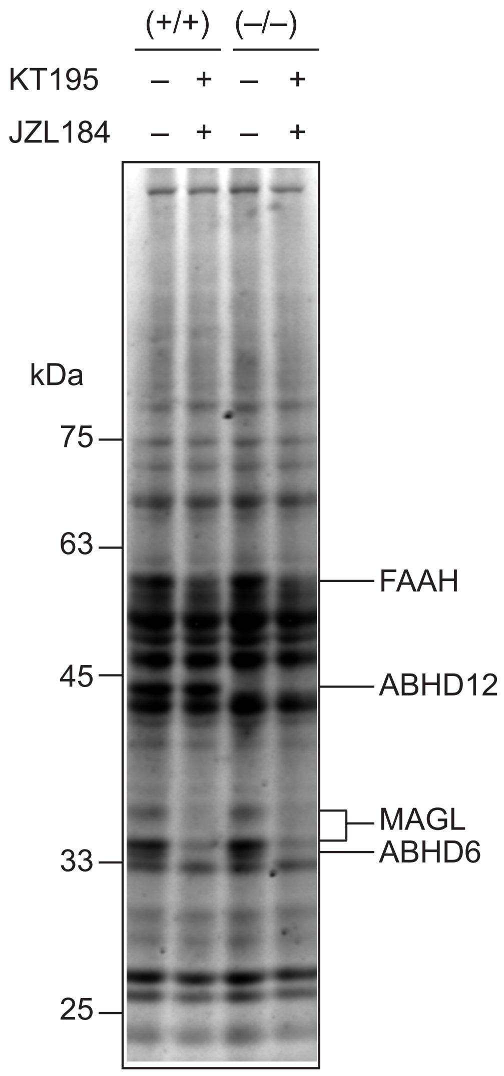

Supplementary Figure 2. Gel based ABPP assays showing the inhibition of MAGL and ABHD6 following JZL184 and KT195 treatment in brain membrane lysates respectively. The brain membrane lysates (1 mg/mL, 100 µL) from wild type (+/+) and ABHD12 knockout (–/–) mice were treated with 1 µM of each JZL184 and KT195 using established gel based ABPP protocols. Following this treatment loss of both MAGL and ABHD6 was observed in brain membrane lysates from wild type (+/+) and ABHD12 knockout (–/–) mice. FAAH is a known off-target of JZL184, and is inhibited in both brain membrane lysates. This experiment was done in biological triplicates with reproducible results.

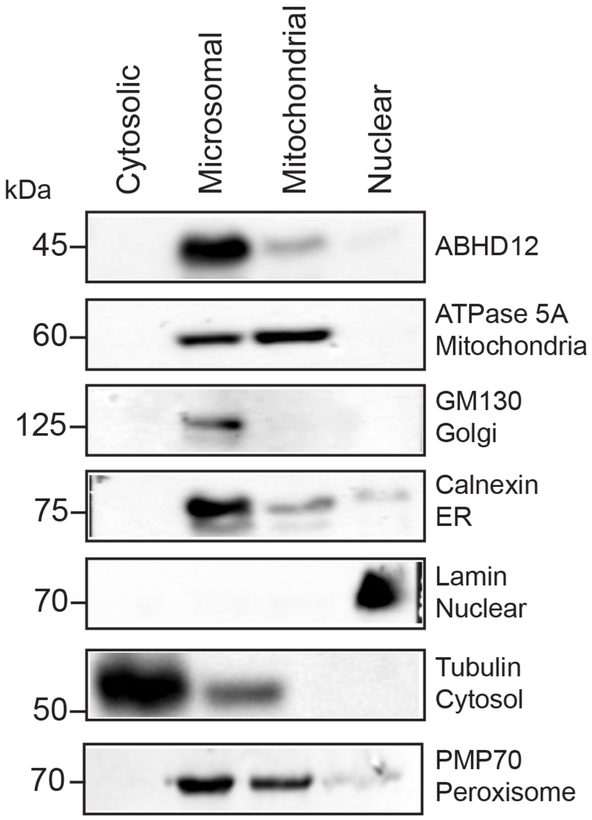

Supplementary Figure 3. Cellular organelle fractionation studies showing enrichment of ABHD12 in the microsomal fraction of MCF7 cells. All the cellular fractionation experiments were done in biological triplicates with reproducible results.

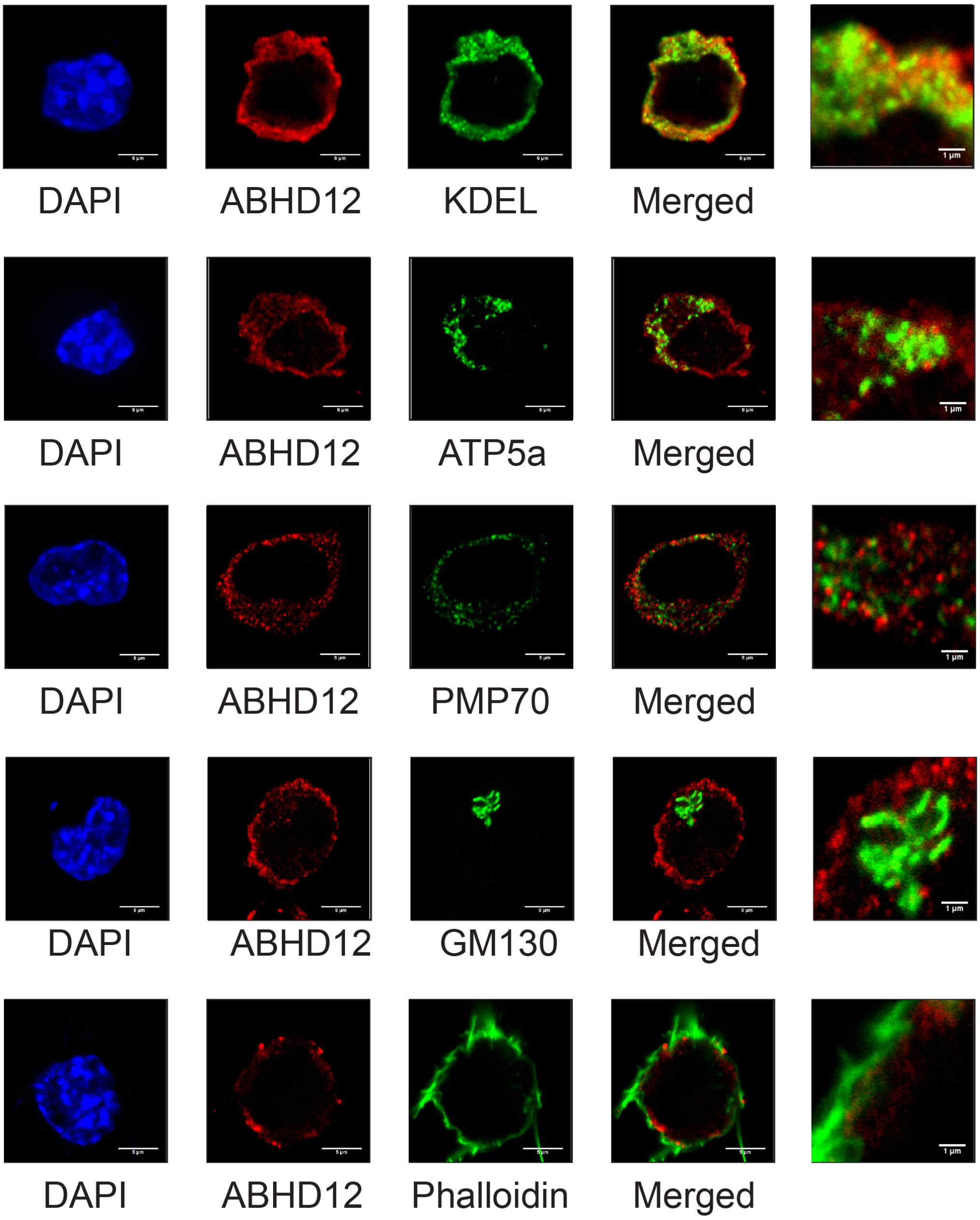

Supplementary Figure 4. Cellular immunofluorescence studies by confocal microscopy showing the ER localization (co-localization with KDEL, ER marker) of ABHD12 in the Neuro-2a cells. All the cellular immunofluorescence experiments were done in at least 4 biological replicates with reproducible results

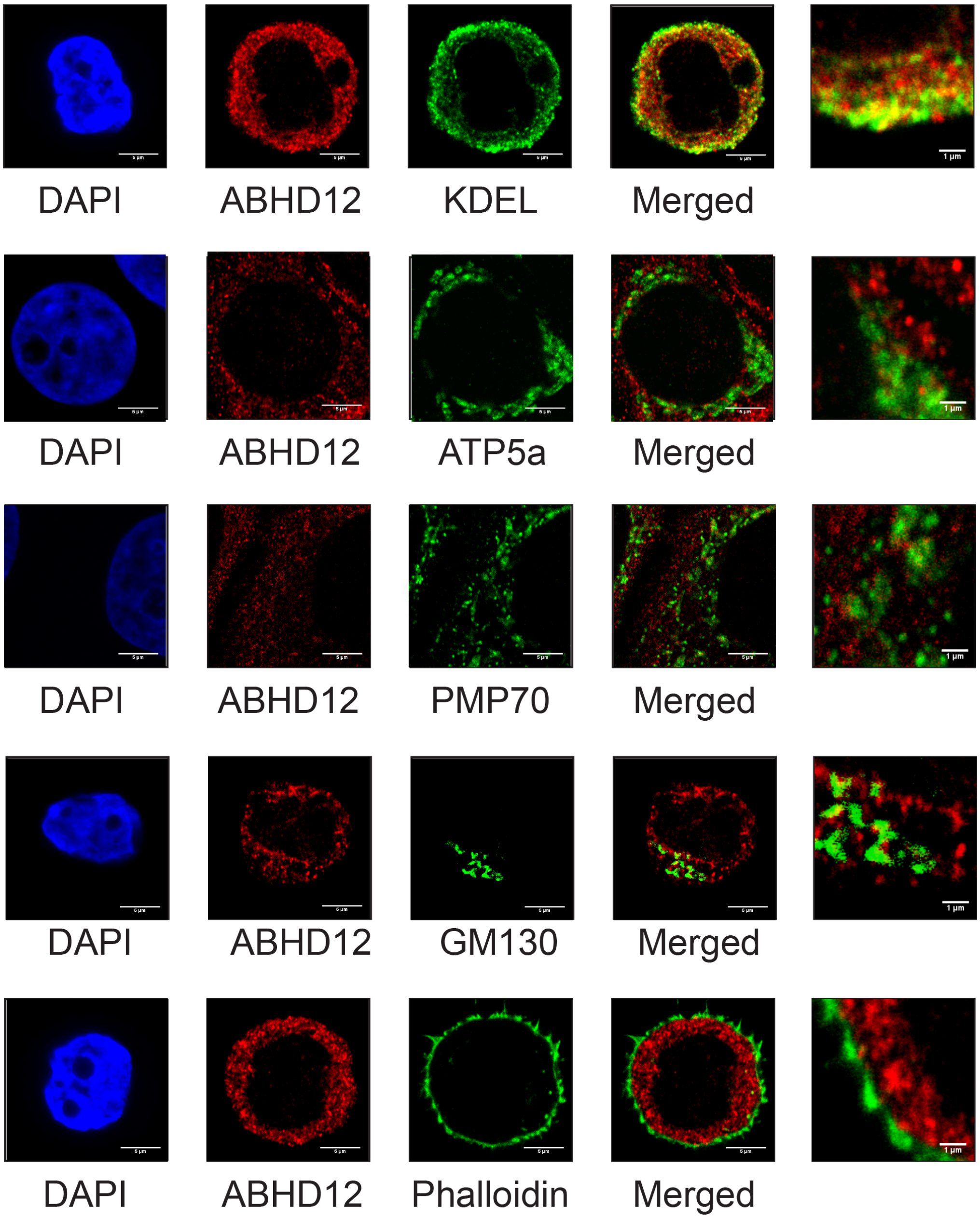

Supplementary Figure 5. Cellular immunofluorescence studies by confocal microscopy showing the ER localization (co-localization with KDEL, ER marker) of ABHD12 in the MCF7 cells. All the cellular immunofluorescence experiments were done in at least 4 biological replicates with reproducible results.

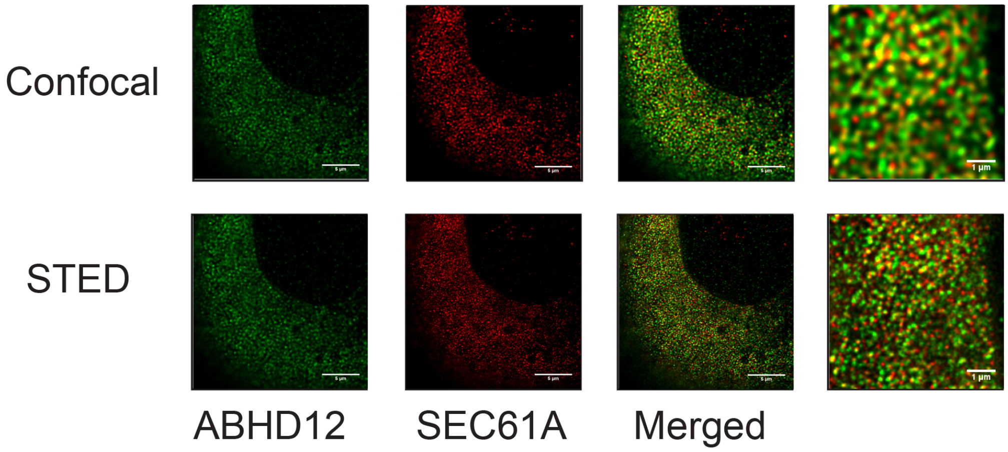

Supplementary Figure 6. High-resolution cellular immunofluorescence studies using STED microscopy in MCF7 cells, showing that ABHD12 has strong co-localization with SEC61A, an ER-marker (yellow in merged image).

Supplementary Table 1

Free fatty acid species	Mean area from five replicate run			
	1 pmol ($x 10^4$)	10 pmol ($x 10^5$)	100 pmol ($x 10^6$)	1000 pmol ($x 10^7$)
C10:0	4.5	3.9	4.3	3.8
C12:0	4.3	4.1	3.5	3.7
C14:0	4.8	3.5	3.7	4.3
C16:0	5.1	3.8	3.8	4.1
C17:1	4.7	4.3	3.5	4.3
C18:0	4.8	4.2	3.9	4.5
C18:1	4.3	4.1	3.8	4.1
C18:3	4.1	4.3	3.7	4.3
C20:0	5.1	3.5	3.6	3.8
C20:1	4.8	3.7	4.1	3.8
C20:4	4.4	3.9	4.3	3.9
C22:0	4.3	3.3	3.7	4.2
C22:1	4.5	3.5	3.8	4.3
C22:4	4.2	4.3	3.8	4.1
C22:6	3.9	3.8	3.9	3.9
C24:0	4.1	3.6	3.5	3.7
C24:1	4.3	3.8	3.9	4.0

The data shows that the mean areas from five independent experiments for different free fatty acids tested in our LC-MS method to ensure that all the free fatty acids tested in this assay have the comparable detection efficiencies in our assay. Based on the data, it is clear that all free fatty acids “fly” the same in the MS, and hence C17:1 free fatty acid can be used as a quantitative standard in this assay. Additionally this result also shows a nice linear dynamic range for measuring free fatty acids from 1 pmol to 1 nmol, which is within our experimental condition.
