# Supplementary material for: Biochemical characterization of the PHARC-associated serine hydrolase ABHD12 reveals its preference for very-long-chain lipids
Source: J Biol Chem. 2018 Sep 20;293(44):16953–63. doi: 10.1074/jbc.RA118.005640 (PMC6217928; doi:10.1074/jbc.RA118.005640)

(2,2-Dimethyl-1,3-dioxolan-4-yl)methyl decanoate (1a)

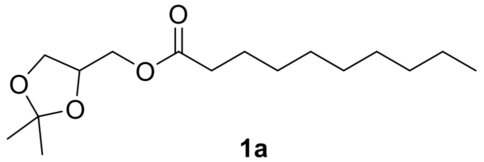

1a

According to general procedure **1a** (49 mg, 100% yield) as a yellowish white solid was prepared from the corresponding decanoic acid: ^{1}H NMR (400 MHz, $CDCl_3$) δ 4.35−4.29 (m, 1H), 4.17 (dd, *J* = 4.72, 11.5 Hz, 1H), 4.11−4.06 (m, 2H), 3.74 (dd, *J* = 6.1, 8.4 Hz, 1H), 2.34 (t, *J* = 7.4 Hz, 2H), 1.66−1.59 (m, 2H), 1.44 (s, 3H), 1.37 (s, 3H), 1.25 (br, 12H), 0.88 (t, *J* = 6.6 Hz, 3H).

Synthesis of 2,3-Dihydroxypropyl decanoate (1a')

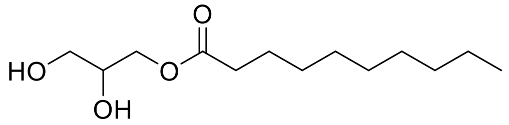

1a'

According to general procedure **1a'** (27 mg, 65% yield) as a yellowish white solid was prepared from the corresponding (2,2-dimethyl-1,3-dioxalan-4-yl)methyl decanoate (**1a**): ^{1}H NMR (400 MHz, $CDCl_3$) δ 4.20 (dd, *J* = 4.7, 11.6 Hz, 1H), 4.14 (dd, *J* = 6.0, 11.6 Hz, 1H), 3.96−3.91 (m, 1H), 3.70 (dd, *J* = 3.6, 11.4 Hz, 1H), 3.60 (dd, *J* = 5.8, 11.4 Hz, 1H), 2.80 (s, 1H), 2.41 (s, 1H), 2.35 (t, *J* = 7.4 Hz, 2H), 1.66−1.59 (m, 2H), 1.26 (br, 12H), 0.88 (t, *J* = 6.6 Hz, 3H); ^{13}C NMR (400 MHz, $CDCl_3$) δ 174.5, 70.4, 65.3, 63.5, 34.3, 32.0, 29.8, 29.5, 29.4, 29.2, 25.0, 22.8, 14.2; HRMS-ESI: $[M + H]^+$ calcd for $C_{13}H_{26}O_4$, 247.1904; found, 247.1897.

(2,2-Dimethyl-1,3-dioxolan-4-yl)methyl dodecanoate (1b)

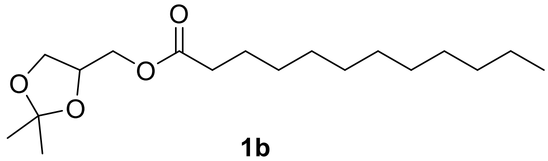

1b

According to general procedure **1b** (47 mg**,** 100%) as a yellowish white solid was prepared from the corresponding dodecanoic acid: ^{1}H NMR (400 MHz, $CDCl_3$) δ 4.35−4.29 (m, 1H), 4.17 (dd, *J* = 4.67,11.4 Hz, 1H), 4.11−4.06 (m, 2H), 3.74 (dd, *J* = 6.3, 8.4 Hz, 1H), 2.34 (t, *J* = 7.4 Hz, 2H), 1.66−1.59 (m, 2H), 1.43 (s, 3H), 1.37 (s, 3H), 1.29−1.26 (m, 16H), 0.88 (t, *J* = 6.6 Hz, 3H).

Synthesis of 2,3-Dihydroxypropyl dodecanoate (1b')

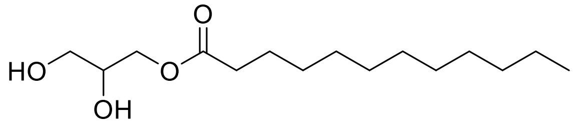

1b'

According to general procedure **1b'** (27 mg, 65% yield) as a yellowish white solid was prepared from the corresponding (2,2-dimethyl-1,3-dioxalan-4-yl)methyl dodecanoate (**1b**): ^{1}H NMR (400 MHz, $CDCl_3$) δ 4.19 (dd, *J* = 4.7, 11.6 Hz, 1H), 4.14 (dd, *J* = 6.0, 11.6 Hz, 1H), 3.96-3.91 (m, 1H), 3.70 (dd, *J* = 3.8, 11.5 Hz, 1H), 3.59 (dd, *J* = 5.8, 11.5 Hz, 1H), 2.88 (s, 1H), 2.51 (s, 1H), 2.35 (t, *J* = 7.4 Hz, 2H), 1.66-1.59 (m, 2H), 1.26 (br, 16H), 0.88 (t, *J* = 6.6 Hz, 3H); ^{13}C NMR (400 MHz, $CDCl_3$) δ 174.5, 70.4, 65.2, 63.5, 34.3, 32.0, 29.7, 29.6 (2C), 29.5, 29.4, 29.3, 25.0, 22.8, 14.2; HRMS-ESI: $[M + H]^+$ calcd for $C_{15}H_{30}O_4$, 275.2217; found, 275.2209.

(2,2-Dimethyl-1,3-dioxolan-4-yl)methyl tetradecanoate (1c)

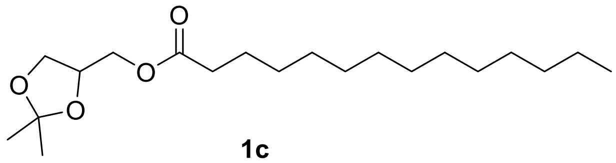

1c

According to general procedure **1c** (44 mg, 91% yield) as a yellowish white solid was prepared from the corresponding myristic acid: ^{1}H NMR (400 MHz, $CDCl_3$) δ 4.35−4.29 (m, 1H), 4.16 (dd, *J* = 4.6, 11.4 Hz, 1H), 4.11−4.06 (m, 2H), 3.74 (dd, *J* = 6.2, 8.4 Hz, 1H), 2.34 (t, *J* = 7.4 Hz, 2H), 1.66−1.59 (m, 2H), 1.43 (s, 3H), 1.37 (s, 3H), 1.26−1.29 (m, 20H), 0.88 (t, *J* = 6.6 Hz, 3H).

Synthesis of 2,3-Dihydroxypropyl tetradecanoate (1c')

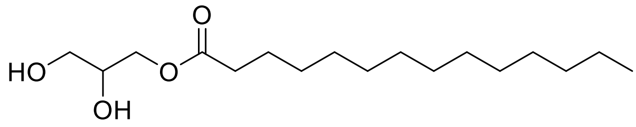

1c'

According to general procedure **1c'** (25 mg, 72% yield) as a yellowish white solid was prepared from the corresponding (2,2-dimethyl-1,3-dioxalan-4-yl)methyl tetradecanoate (**1c**): ^{1}H NMR (400 MHz, $CDCl_3$) δ 4.21 (dd, *J* = 4.6, 11.6 Hz, 1H), 4.15 (dd, *J* = 6.0, 11.6 Hz, 1H), 3.94 (s, 1H), 3.70 (d, *J* = 11.3 Hz, 1H), 3.60 (d, *J* = 3.9 Hz, 1H), 2.56 (s, 1H), 2.35 (t, *J* = 7.4 Hz, 2H), 2.12 (s, 1H, 1.66−1.59 (m, 2H), 1.26 (br, 20H), 0.88 (t, *J* = 6.6 Hz, 3H); ^{13}C NMR

(400 MHz, $CDCl_3$) δ 174.5, 70.4, 65.2, 63.5, 34.3, 32.1, 29.8, 29.8, 29.7, 29.6 (2C), 29.5, 29.4, 29.3, 25.1, 22.8, 14.3; HRMS-ESI: $[M + H]^+$ calcd for $C_{17}H_{34}O_4$, 303.2530; found, 303.2520.

(2,2-Dimethyl-1,3-dioxolan-4-yl)methyl palmitate (1d)

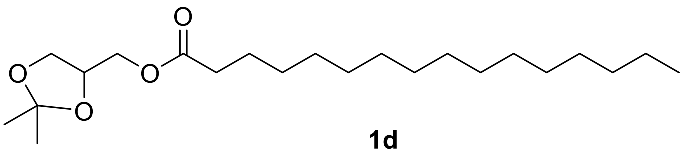

1d

According to general procedure **1d** (20 mg, 100% yield) as a white solid was prepared from the corresponding palmitic acid: ^{1}H NMR (400 MHz, $CDCl_3$) δ 4.35−4.29 (m, 1H), 4.17 (dd, *J* = 4.7, 11.4 Hz, 1H), 4.11−4.06 (m, 2H), 3.74 (dd, *J* = 6.2, 8.4 Hz, 1H), 2.34 (t, *J* = 7.4 Hz, 2H), 1.66−1.59 (m, 2H), 1.44 (s, 3H), 1.37 (s, 3H), 1.25 (br, 24H), 0.88 (t, *J* = 6.6 Hz, 3H).

Synthesis of 2,3-Dihydroxypropyl palmitate (1d')

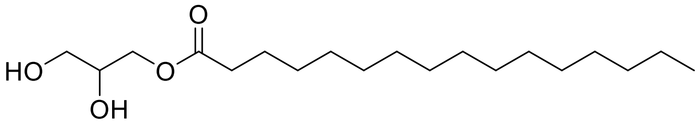

1d'

According to general procedure **1d'** (16 mg, 94% yield) as a white solid was prepared from the corresponding (2,2-dimethyl-1,3-dioxalan-4-yl)methyl palmitate (**1d**): ^{1}H NMR (400 MHz, $CDCl_3$) δ 4.21 (dd, *J* = 4.6, 11.6 Hz, 1H), 4.15 (dd, *J* = 6.0, 11.6 Hz, 1H), 3.96−3.91 (m, 1H), 3.70 (dd, *J* = 3.7, 11.3 Hz, 1H), 3.60 (dd, *J* = 5.7, 11.4 Hz, 1H), 2.60 (s, 1H), 2.35 (t, *J* = 7.4 Hz, 2H), 2.17 (s, 1H), 1.65−1.59 (m, 2H), 1.26 (br, 24H), 0.88 (t, *J* = 6.6 Hz, 3H); ^{13}C NMR (400 MHz, $CDCl_3$) δ 174.5, 70.4, 65.3, 63.5, 34.3, 32.1, 29.8, 29.8, 29.8, 29.8, 29.7, 29.6 (2C), 29.5, 29.4, 29.3, 25.1, 22.8, 14.3; HRMS-ESI: $[M + H]^+$ calcd for $C_{19}H_{38}O_4$, 331.2843; found, 331.2847.

(2,2-Dimethyl-1,3-dioxolan-4-yl)methyl stearate (1e)

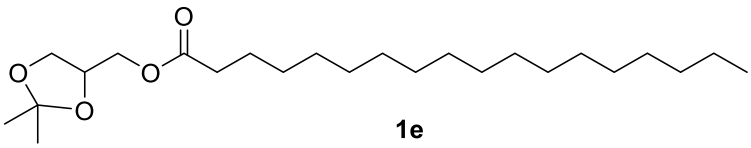

1e

According to general procedure **1e** (20 mg, 100% yield) as a white solid was prepared from the corresponding stearic acid: ^{1}H NMR (400 MHz, $CDCl_3$) δ 4.34−4.29 (m, 1H), 4.17 (dd, *J*

= 4.7,11.4 Hz, 1H), 4.11−4.06 (m, 2H), 3.74 (dd, *J* = 6.1, 8.4 Hz, 1H), 2.34 (t, *J* = 7.4 Hz, 2H), 1.66−1.59 (m, 2H), 1.44 (s, 3H), 1.37 (s, 3H), 1.25 (br, 28H), 0.88 (t, *J* = 6.6 Hz, 3H).

Synthesis of 2,3-Dihydroxypropyl stearate (1e')

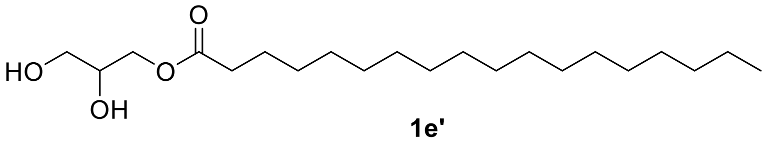

1e'

According to general procedure **1e'** (20 mg, 71%) as a white solid was prepared from the corresponding (2,2-dimethyl-1,3-dioxalan-4-yl)methyl stearate (**1e**): ^{1}H NMR (400 MHz, $CDCl_3$) δ 4.20 (dd, *J* = 4.4, 11.5 Hz, 1H), 4.15 (dd, *J* = 6.0, 11.6 Hz, 1H), 3.94−3.92 (m, 1H), 3.70 (dd, *J* = 3.7, 11.3 Hz, 1H), 3.60 (dd, *J* = 5.7, 11.4 Hz, 1H), 2.61(s, 1H), 2.35(t, *J* = 7.4 Hz, 2H), 2.17 (s, 1H), 1.64−1.59 (m, 2H), 1.25 (br, 28H), 0.88 (t, *J* = 6.6 Hz, 3H); ^{13}C NMR (400 MHz, $CDCl_3$) δ 174.5, 70.4, 65.3, 63.5, 34.3, 32.1, 29.8 (5C), 29.7, 29.6 (2C), 29.5 (2C), 29.4, 29.3, 25.1, 22.8, 14.3; HRMS-ESI: $[M + H]^+$ calcd for $C_{21}H_{42}O_4$, 359.3156; found, 359.3155.

(2,2-Dimethyl-1,3-dioxolan-4-yl)methyl oleate (1f)

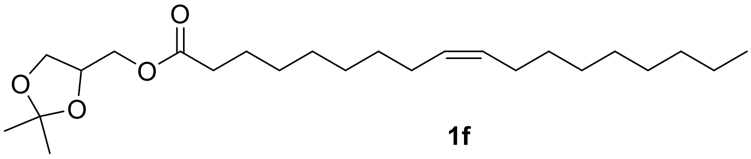

1f

According to general procedure **1f** (20 mg, 71.4% yield) as a yellowish semisolid was prepared from the corresponding oleic acid: ^{1}H NMR (400 MHz, $CDCl_3$) δ 5.36−5.33 (m, 2H), 4.35−4.29 (m, 1H), 4.17 (dd, *J* = 4.6,11.5 Hz, 1H), 4.11−4.06 (m, 2H), 3.74 (dd, *J* = 6.2, 8.4 Hz, 1H), 2.34 (t, *J* = 7.4 Hz, 2H), 2.05−1.98 (m, 4H), 1.64−1.58 (m, 2H), 1.44 (s, 3H), 1.37 (s, 3H), 1.25 (br, 20H), 0.88 (t, *J* = 6.4 Hz, 3H).

Synthesis of 2,3-Dihydroxypropyl oleate (1f')

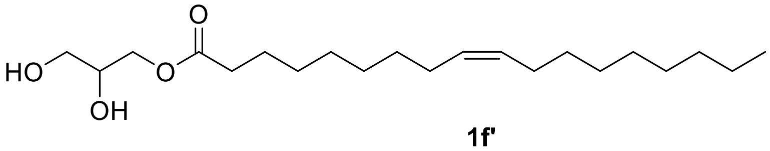

1f'

According to general procedure **1f'** (12 mg, 71% yield) as a yellowish semisolid was prepared from the corresponding (2,2-dimethyl-1,3-dioxalan-4-yl)methyl oleate (**1f**): ^{1}H NMR (400 MHz, $CDCl_3$) δ 5.41−5.30 (m, 2H), 4.20 (dd, *J* = 6.0, 11.6 Hz, 1H), 4.14 (dd, *J* = 6.0,

11.6 Hz, 1H), 3.96−3.91 (m, 1H), 3.69 (dd, J = 3.8, 11.4 Hz, 1H), 3.59 (dd, J = 5.8, 11.4 Hz, 1H), 2.69 (s, 1H), 2.35(t, J = 7.4 Hz, 2H), 2.07−1.99 (m, 4H), 1.74 (s, 1H), 1.65−1.59 (m, 2H), 1.26-1.30 (m, 20H), 0.88 (t, J = 6.6 Hz, 3H); ^{13}C NMR (400 MHz, $CDCl_3$) δ 174.5, 130.2, 129.8, 70.4, 65.3, 63.5, 34.3, 32.0, 29.9, 29.8, 29.7 (2C), 29.5 (2C), 29.3, 29.2, 27.3, 27.4, 25.0, 22.8, 14.3; HRMS-ESI: $[M + H]^+$ calcd for $C_{21}H_{40}O_4$, 357.2999; found, 357.2995.

(2,2-Dimethyl-1,3-dioxoalan-4-yl)methyl (9Z,12Z,15Z)-octadeca-9,12,15-trienoate (1g)

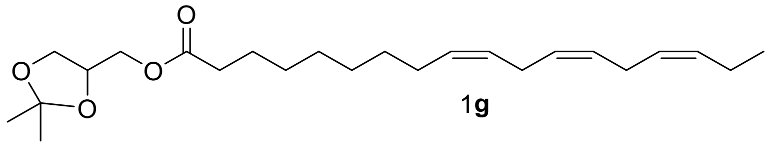

1g

According to general procedure **1g** (14mg, 100% yield) as a yellowish semisolid was prepared from the corresponding α-linolenic acid: ^1H NMR (400 MHz, $CDCl_3$) δ 5.43-5.27 (m, 6H), 4.34-4.28 (m, 1H), 4.17 (dd, J = 4.6,11.5 Hz, 1H), 4.10-4.06 (m, 2H), 3.73 (dd, J = 6.3, 8.4 Hz, 1H), 2.82-2.76 (m, 4H) 2.34 (t, J = 7.4 Hz, 2H), 2.11-2.02 (m, 4H), 1.64-1.59 (m, 2H), 1.43 (s, 3H), 1.37 (s, 3H), 1.35−1.25 (m, 8H), 0.97 (t, J = 7.4 Hz, 3H).

Synthesis of 2,3-Dihydroxypropyl (9Z, 12Z, 15Z)-octadeca-9,12,15-trienoate (1g')

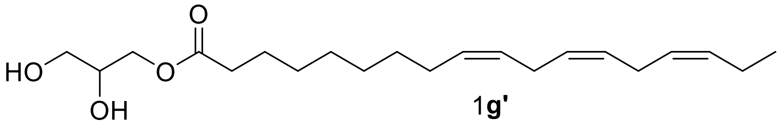

1g'

According to general procedure **1g'** (8 mg, 83% yield) as a yellowish semisolid was prepared from the corresponding (2,2-dimethyl-1,3-dioxoalan-4-yl)methyl oleate (**1g**): ^1H NMR (400 MHz, $CDCl_3$) δ 5.43−5.28 (m, 6H), 4.21 (dd, J = 6.0, 11.6 Hz, 1H), 4.15 (dd, J = 6.0, 11.6 Hz, 1H), 3.95−3.90 (m, 1H), 3.70 (dd, J = 3.8, 11.4 Hz, 1H), 3.60 (dd, J = 5.8, 11.4 Hz, 1H), 2.82−2.79 (m, 4H), 2.57 (s, 1H), 2.35 (t, J = 7.4 Hz, 2H), 2.09−2.02 (m, 4H), 1.65−1.59 (m, 2H), 1.31 (br, 8H), 0.97 (t, J = 7.5 Hz, 3H); ^{13}C NMR (400 MHz, $CDCl_3$) δ 174.4, 132.1, 130.4, 128.4, 128.4, 127.9, 127.3, 70.4, 65.3, 63.5, 34.3, 29.7, 29.3 (2C), 29.2, ,27.3, 25.8, 25.7, 25.0, 20.7, 14.4; HRMS-ESI: $[M + H]^+$ calcd for $C_{21}H_{36}O_4$, 353.2686; found, 353.2687.

(2,2-Dimethyl-1,3-dioxolan-4-yl)methyl icosanoate (1h)

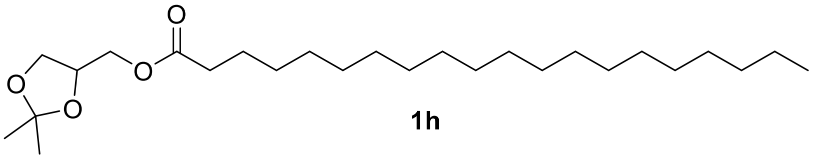

1h

According to general procedure **1h** (20 mg, 100% yield) as a white solid was prepared from the corresponding arachidic acid: ^{1}H NMR (400 MHz, $CDCl_3$) δ 4.35−4.29 (m, 1H), 4.17 (dd, *J* = 4.7,11.5 Hz, 1H), 4.11−4.06 (m, 2H), 3.74 (dd, *J* = 6.1, 8.4 Hz, 1H), 2.34 (t, *J* = 7.4 Hz, 2H), 1.64−1.59 (m, 2H), 1.43 (s, 3H),1.37 (s, 3H), 1.25 (br, 32H), 0.88 (t, *J* = 6.6 Hz, 3H).

Synthesis of 2,3-Dihydroxypropyl icosanoate (1h')

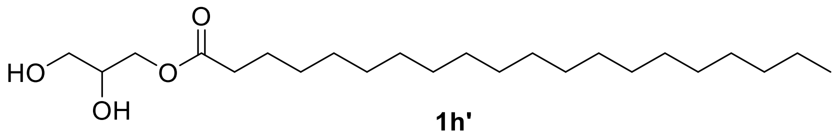

1h'

According to general procedure **1h'** (16 mg, 89% yield) as a white solid was prepared from the corresponding (2,2-dimethyl-1,3-dioxalan-4-yl)methyl icosanoate (**1h**): ^{1}H NMR (400 MHz, $CDCl_3$) δ 4.21 (dd, *J* = 4.6, 11.6 Hz, 1H), 4.15 (dd, *J* = 6.0, 11.6 Hz, 1H), 3.93 (s, 1H), 3.68 (d, *J* = 11.0 Hz, 1H), 3.60 (dd, *J* = 5.3, 11.2 Hz, 1H), 2.57 (s, 1H), 2.35 (t, *J* = 7.4 Hz, 2H), 2.17 (s, 1H), 1.63−1.59 (m, 2H), 1.25 (br, 32H), 0.88 (t, *J* = 6.6 Hz, 3H); ^{13}C NMR (400 MHz, $CDCl_3$) δ 174.5, 70.4, 65.3, 63.5, 34.3, 32.1, 29.8 (9C), 29.7, 29.6, 29.5, 29.4, 29.3, 25.1, 22.8, 14.3; HRMS-ESI: $[M + H]^+$ calcd for $C_{23}H_{46}O_4$, 387.3469; found, 387.3464.

(2,2-Dimethyl-1,3-dioxolan-4-yl)methyl (*Z*)-icos-11-enoate (1i)

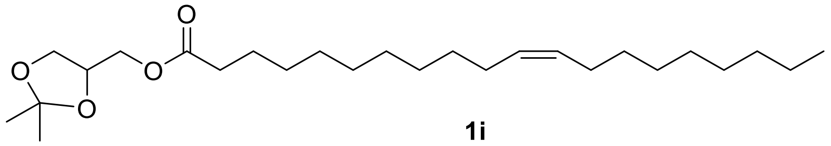

1i

According to general procedure **1i** (18 mg, 67% yield) as a white solid was prepared from the corresponding eicosenoic acid: ^{1}H NMR (400 MHz,$CDCl_3$) δ 5.36−5.33 (m, 2H),4.35−4.29 (m, 1H), 4.17 (dd, *J* = 4.7,11.5 Hz, 1H), 4.11−4.06 (m, 2H), 3.74 (dd, *J* = 6.1, 8.4 Hz, 1H), 2.34 (t, *J* = 7.4 Hz, 2H), 2.03−1.99 (m, 4H), 1.66−1.59 (m, 2H), 1.44 (s, 3H), 1.37 (s, 3H), 1.25 (br, 24H), 0.88 (t, *J* = 6.4 Hz, 3H).

Synthesis of 2,3-Dihydroxypropyl (*Z*)-icos-enoate (1i')

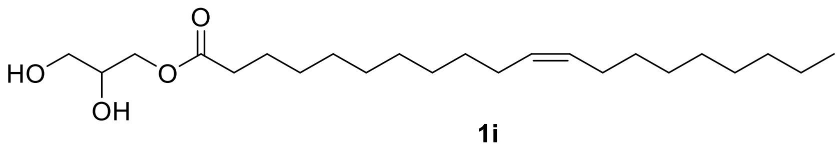

1i

According to general procedure **1i'** (8 mg, 50%) as a white solid was prepared from the corresponding (2,2-dimethyl-1,3-dioxalan-4-yl)methyl (*Z*)-icos-11-enoate (**1i**): ^{1}H NMR (400 MHz, $CDCl_3$) δ 5.39−5.31 (m, 2H), 4.21 (dd, *J* = 4.6, 11.6 Hz, 1H), 4.15 (dd, *J* = 6.1, 11.6 Hz,

1H), 3.93 (s, 1H), 3.71−3.69 (m, 1H), 3.59 (dd, *J* = 5.6, 11.3 Hz, 1H), 2.54 (s, 1H), 2.35 (t, *J* = 7.4 Hz, 2H), 2.10 (s, 1H) 2.03−1.99 (m, 4H), 1.68−1.61 (m, 2H), 1.27 (d, 24H), 0.88 (t, *J* = 6.6 Hz, 3H); ^{13}C NMR (400 MHz, $CDCl_3$) δ 174.5, 130.1, 130.0, 70.4, 65.3, 63.5, 34.3, 32.1, 29.9, 29.7 (2C), 29.6, 29.6, 29.5 (2C), 29.4, 29.4, 29.3, 27.4 (2C), 25.1, 22.8, 14.3; HRMS-ESI: $[M + H]^+$ calcd for $C_{23}H_{44}O_4$, 385.3312; found, 385.3315.

(2,2-Dimethyl-1,3-dioxalan-4-yl)methyl (5*Z*,8*Z*,11*Z*,14*Z*)-icosa-5,8,11,14-tetraenoate (1j)

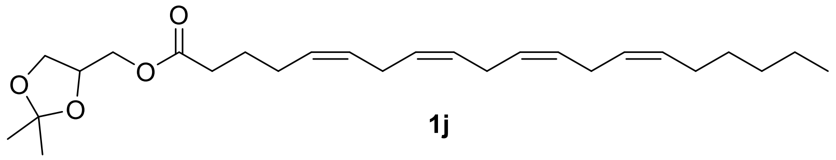

1j

According to general procedure **1j** (9 mg, 69% yield) as a yellowish semisolid was prepared from the corresponding arachidonic acid: ^{1}H NMR (400 MHz, $CDCl_3$) δ 5.43−5.30 (m, 8H), 4.34−4.29 (m, 1H), 4.17 (dd, *J* = 4.6,11.4 Hz, 1H), 4.11−4.06 (m, 2H), 3.74 (dd, *J* = 6.2, 8.4 Hz, 1H), 2.85−2.79 (m, 6H), 2.34 (t, *J* = 7.4 Hz, 2H), 2.17−2.03 (m, 4H), 1.75−1.68 (m, 2H), 1.41 (s, 3H), 1.37 (s, 3H), 1.23 (br, 6H), 0.88 (t, *J* = 6.7 Hz, 3H).

Synthesis of 2,3-Dihydroxypropyl (5*Z*,8*Z*,11*Z*,14*Z*)-icosa-5,8,11,14-tetraenoate (1j')

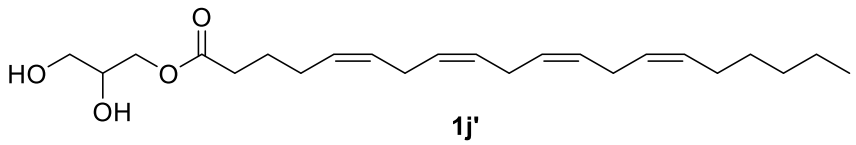

1j'

According to general procedure **1j'** (3 mg, 50% yield) as a yellowish semisolid was prepared from the corresponding (2,2-dimethyl-1,3-dioxalan-4-yl)methyl (5*Z*,8*Z*,11*Z*,14*Z*)-icosa-5,8,11,14-tetraenoate (**1j**): ^{1}H NMR (400 MHz, $CDCl_3$) δ 5.44−5.30 (m, 8H), 4.21 (dd, *J* = 6.1, 11.6 Hz, 1H), 4.15 (dd, *J* = 4.6, 11.6 Hz, 1H), 3.93 (m, 1H), 3.70 (dd, *J* = 3.6, 11.6 Hz, 1H), 3.60 (dd, *J* = 5.7, 11.4 Hz, 1H), 2.84 (dd, *J* = 5.4, 11.4 Hz, 6H), 2.47 (s, 1H), 2.37(t, *J* = 7.4 Hz, 2H), 2.17−2.01 (m, 4H), 1.76−1.69 (m, 2H), 1.62 (s, 1H), 1.25 (br, 6H), 0.89 (t, *J* = 6.7 Hz, 3H); ^{13}C NMR (400 MHz, $CDCl_3$) δ 174.2, 130.7, 129.2, 128.9, 128.8, 128.4, 128.2, 128.0, 127.7, 70.4, 65.4, 63.5, 33.6, 31.7, 29.9, 29.5, 27.4, 26.7, 25.8 (2C), 24.9, 22.7, 14.2**.** HRMS-ESI: $[M + H]^+$ calcd for $C_{23}H_{38}O_4$, 379.2843; found, 379.2834.

(2,2-Dimethyl-1,3-dioxolan-4-yl)methyl docosanoate (1k)

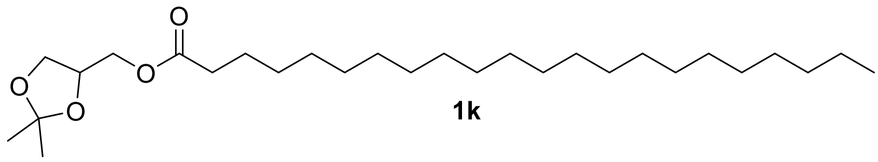

1k

According to general procedure **1k** (18 mg, 75% yield) as a white solid was prepared from the corresponding behenic acid: ^{1}H NMR (400 MHz, $CDCl_3$) δ 4.35−4.29 (m, 1H), 4.17 (dd, *J* = 4.7,11.6 Hz, 1H), 4.11−4.06 (m, 2H), 3.74 (dd, *J* = 6.6, 8.4 Hz, 1H), 2.34 (t, *J* = 7.4 Hz, 2H), 1.69−1.59 (m, 2H), 1.44 (s, 3H), 1.37 (s, 3H), 1.25 (br, 36H), 0.88 (t, *J* = 6.6 Hz, 3H).

Synthesis of 2,3-Dihydroxypropyl docosanoate (1k')

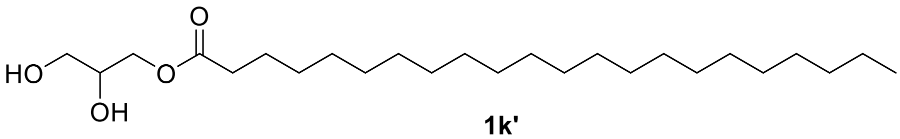

1k'

According to general procedure **1k'** (10 mg, 77% yield) as a white solid was prepared from the corresponding (2,2-dimethyl-1,3-dioxalan-4-yl)methyl docosanoate (**1k**): ^{1}H NMR (400 MHz, $CDCl_{3,}$) δ 4.20 (dd, *J* = 4.7, 11.6 Hz, 1H), 4.14 (dd, *J* = 6.1, 11.6 Hz, 1H), 3.93 (d, *J* = 4.8 Hz, 1H), 3.70 (d, *J* = 11.4 Hz, 1H), 3.59 (dd, *J* = 5.76, 11.4 Hz, 1H), 2.47 (d, *J* = 5.0 Hz, 1H), 2.35 (t, *J* = 7.4 Hz, 2H), 2.04 (s, 1H), 1.68 −1.57 (m, 2H), 1.25 (br, 36H), 0.88 (t, *J* = 6.6 Hz, 3H); ^{13}C NMR (400 MHz, $CDCl_3$) δ 174.5, 70.4, 65.3, 63.5, 34.3, 32.1, 29.9 (2C), 29.8 (3C), 29.6, 29.6, 29.5 (3C), 29.4 (3C), 29.3 (3C), 25.1, 22.8, 14.3; HRMS-ESI: $[M + H]^+$ calcd for $C_{25}H_{50}O_4$, 415.3782; found, 415.3781.

(2,2-Dimethyl-1,3-dicoxolan-4-yl)methyl (*Z*)-docos-13-enoate (1l)

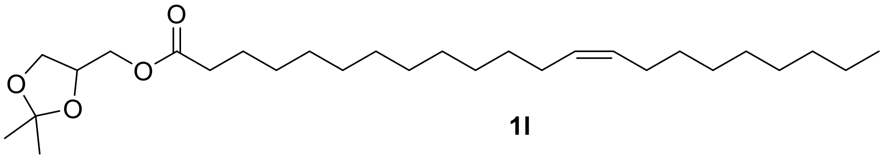

1l

According to general procedure **1l** (18 mg, 69% yield) as a white solid was prepared from the corresponding erucic acid: ^{1}H NMR (400 MHz, $CDCl_3$) δ 5.39−5.31 (m, 2H), 4.34−4.29 (m, 1H), 4.17 (dd, *J* = 4.7, 11.4 Hz, 1H), 4.11−4.06 (m, 2H), 3.74 (dd, *J* = 6.2, 8.4 Hz,1H), 2.34 (t, *J* = 7.4 Hz, 2H), 2.04−1.96 (m, 4H), 1.66−1.59 (m, 2H), 1.44 (s, 3H), 1.37 (s, 3H), 1.26 (br, 28H), 0.88 (t, *J* = 6.4 Hz, 3H).

Synthesis of 2,3-Dihydroxypropyl (*Z*)-docos-13-enoate (1l')

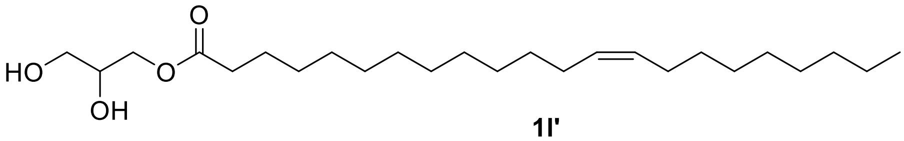

1l'

According to general procedure **1l'** (12 mg, 75% yield) as a white solid was prepared from the corresponding (2,2-dimethyl-1,3-dioxalan-4-yl)methyl (*Z*)-docos-13-enoate (**1l**): ^{1}H NMR

(400 MHz, $CDCl_3$) δ 5.39−5.31 (m, 2H), 4.21 (dd, *J* = 4.6, 11.6 Hz, 1H), 4.15 (dd, *J* = 6.0, 11.6 Hz, 1H), 3.96−3.91 (m, 1H), 3.70 (dd, *J* = 3.8, 11.4Hz, 1H), 3.60 (dd, *J* = 5.7, 11.4 Hz, 1H), 2.58 (s, 1H), 2.35(t, *J* = 7.4 Hz, 2H), 2.17 (s, 1H), 2.04−1.99 (m, 4H), 1.65−1.59 (m, 2H), 1.26 (br, 28H), 0.88 (t, *J* = 6.6 Hz, 3H); ^{13}C NMR (400 MHz, $CDCl_3$) δ 174.5, 130.1, 130.1, 130.0, 70.4, 65.3, 63.5, 34.3, 32.1, 29.9, 29.8, 29.7, 29.7, 29.7, 29.6, 29.5 (2C), 29.4 (2C), 29.3, 27.4 (2C), 25.1, 22.8, 14.3; HRMS-ESI: $[M + H]^+$ calcd for $C_{25}H_{48}O_4$, 413.3625; found, 413.3622.

Synthesis of 2,3-Dihydroxypropyl (7*Z*,10*Z*,13*Z*,16*Z*)-docosa-7,10,13,16-tetraenoate(1m')

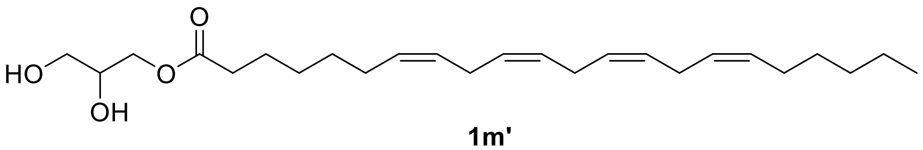

1m'

According to general procedure **1m'** (3 mg, 50% yield) as a yellowish semisolid was prepared from the corresponding (2,2-dimethyl-1,3-dioxolan-4-yl)methyl (7Z,10Z,13Z,16Z)-icosa-7,10,13,17-tetraenoate: ^1H NMR (400 MHz, $CDCl_3$) δ 5.38−5.33 (m, 8H), 4.21 (dd, *J* = 6.6, 10.7 Hz, 1H), 4.14 (dd, *J* = 6.1, 11.6 Hz, 1H), 3.93 (s, 1H), 3.67 (d, *J* = 9.4 Hz, 1H), 3.60 (d, *J* = 6.0 Hz, 1H), 2.87−2.79 (m, 6H), 2.45 (s, 1H), 2.34 (t, *J* = 7.4 Hz, 2H), 2.19−2.16 (m, 1H) 2.07−2.01(m, 4H), 1.68−1.55 (m, 2H), 1.25 (br, 10H), 0.88 (t, *J* = 6.7 Hz, 3H); HRMS-ESI: $[M + H]^+$ calcd for $C_{25}H_{42}O_4$, 407.3156; found, 407.3156.

(2,2-Dimethyl-1,3-dioxolan-4-yl)methyl (4*Z*,7*Z*,10*Z*,13*Z*,16*Z*,19*Z*)-docosa-4,7,10,13,17,19-tetraenoate (1n)

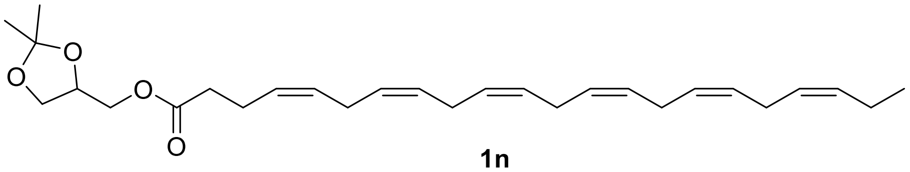

1n

According to general procedure **1n** (6 mg, 47% yield) as a yellowish semisolid was prepared from the corresponding docosahexaenoic acid: ^1H NMR (400 MHz, $CDCl_3$) δ 5.44−5.28 (m, 12H), 4.34−4.28 (m, 1H), 4.17 (dd, *J* = 4.6, 11.4 Hz, 1H), 4.12−4.06 (m, 2H), 3.74 (dd, *J* = 6.1, 8.4 Hz, 1H), 2.88−2.80 (m, 10H), 2.43−2.37 (m, 4H), 2.11−2.04 (m, 2H), 1.43 (s, 3H), 1.37 (s, 3H), 0.97 (t, *J* = 7.1 Hz, 3H).

Synthesis of 2,3-Dihydroxypropyl (4*Z*,7*Z*,10*Z*,13*Z*,16*Z*,19*Z*)-docosa-4,7,10,13,16,19-tetraenoate (1n')

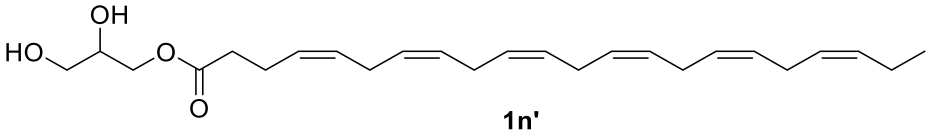

1n'

According to general procedure **1n’** (4 mg, 80% yield) as a yellowish semisolid was prepared from the corresponding (2,2-dimethyl-1,3-dioxolan-4-yl)methyl (4*Z*,7*Z*,10*Z*,13*Z*,16*Z*,19*Z*)-docosa-4,7,10,13,17,19-tetraenoate (**1n**): ^{1}H NMR (400 MHz, $CDCl_3$) δ 5.46−5.28 (m, 12H), 4.24−4.13 (m, 2H), 3.95 (dd, *J* = 5.5, 9.9Hz, 1H), 3.70 (dd *J* = 3.9, 11.4Hz, 1H), 3.59 (dd, *J* = 5.7, 11.4Hz, 1H), 2.84 (dd, *J* = 4.2,15.2 Hz, 10H), 2.46−2.33 (m, 4H), 2.11−2.06 (m, 2H), 2.04 (s, 1H), 1.63 (s, 1H), 0.99 (t, *J* = 7.5 Hz, 3H); ^{13}C NMR (400 MHz, $CDCl_3$) δ 173.7, 132.2, 129.8, 128.7, 128.5 (2C), 128.4, 128.4, 128.2, 128.1, 128.0, 127.8, 127.2, 70.4, 65.5, 63.4, 34.2, 29.9 (2C), 25.8, 25.8, 25.7, 22.9, 20.7, 14.4; HRMS-ESI: $[M + H]^+$ calcd for $C_{25}H_{38}O_4$, 403.2843; found, 403.2837.

(2,2-Dimethyl-1,3-dioxolan-4-yl)methyl tetracosanoate (1o)

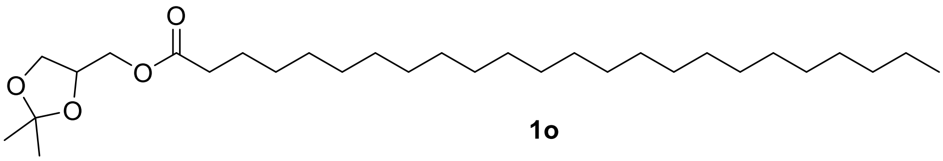

1o

According to general procedure **1o** (18 mg, 69% yield) as a white solid was prepared from the corresponding lignoceric acid: ^{1}H NMR (400 MHz, $CDCl_3$) δ 4.34−4.29 (m, 1H), 4.17 (dd, *J* = 4.7,11.5 Hz, 1H), 4.11−4.06 (m, 2H), 3.73 (dd, *J* = 6.2, 8.4 Hz, 1H), 2.34 (t, *J* = 7.4 Hz, 2H), 1.66−1.59 (m, 2H), 1.43 (s, 3H), 1.37 (s, 3H), 1.25 (br, 40H), 0.88 (t, *J* = 6.6 Hz, 3H).

Synthesis of 2,3-Dihydroxypropyl tetracosanoate (1o’)

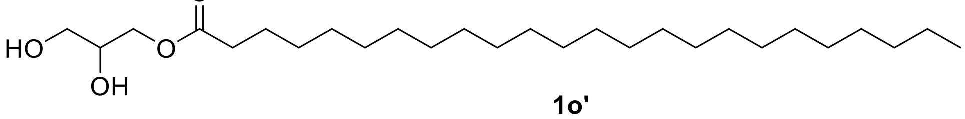

1o'

According to general procedure **1o’** (5 mg, 32% yield) as a white solid was prepared from the corresponding (2,2-dimethyl-1,3-dioxolan-4-yl)methyl tetracosanoate (**1o**): ^{1}H NMR (400 MHz, $CDCl_3$) δ 4.22 (dd, *J* = 4.6, 11.6 Hz, 1H), 4.14 (dd, *J* = 6.0, 11.6 Hz, 1H), 3.94 (s, 1H), 3.70 (d, J = 8.2 Hz, 1H), 3.60 (dd, *J* = 5.4, 11.3 Hz, 1H), 2.50 (s, 1H), 2.35 (t, *J* = 7.5 Hz, 2H), 2.05 (s, 1H), 1.65−1.59 (m, 4H), 1.25 (br, 38H), 0.88 (t, *J* = 6.6 Hz, 3H); ^{13}C NMR (400 MHz, $CDCl_3$) δ 174.5, 70.4, 65.3, 63.5, 34.3, 32.1, 29.9, 29.8, 29.8, 29.6 (6H), 29.5 (6H), 29.4 (2C), 29.3, 25.1, 22.8, 14.3; HRMS-ESI: $[M + H]^+$ calcd for $C_{27}H_{54}O_4$, 443.4095; found, 443.4097.

(2,2-Dimethyl-1,3-dioxolan-4-yl)methyl (*Z*)-tetracos-15-enoate (1p)

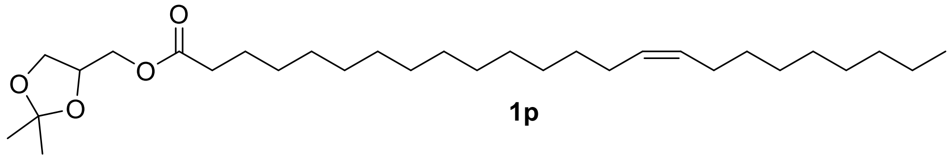

1p

According to general procedure **1p** (18 mg, 69% yield) as a white solid was prepared from the corresponding nervonic acid: ^{1}H NMR (400 MHz, $CDCl_3$) δ 5.39−5.31 (m, 2H), 4.35−4.29 (m, 1H), 4.17 (dd, *J* = 4.6,11.4 Hz, 1H), 4.11−4.06 (m, 2H), 3.74 (dd, *J* = 6.2, 8.4 Hz, 1H), 2.34 (t, *J* = 7.4 Hz, 2H), 2.04−1.99 (m, 4H), 1.66−1.59 (m, 2H), 1.44 (s, 3H), 1.37 (s, 3H), 1.2 (br, 32H), 0.88 (t, *J* = 6.4 Hz, 3H).

Synthesis of 2,3-Dihydroxypropyl (*Z*)-tetracos-15-enoate (1p')

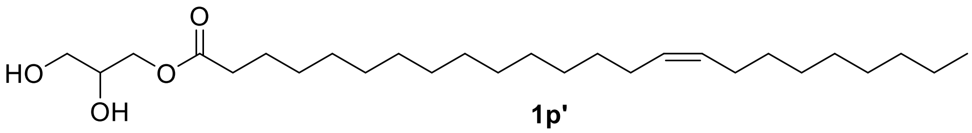

1p'

According to general procedure **1p'** (8 mg, 50% yield) as a white solid was prepared from the corresponding (2,2-dimethyl-1,3-dioxolan-4-yl)methyl (Z)-tetracos-15-enoate (**1p**): ^{1}H NMR (400 MHz, $CDCl_3$) δ 5.39−5.31 (m, 2H), 4.21 (dd, *J* = 4.6, 11.6 Hz, 1H), 4.15 (dd, *J* = 6.0, 11.6 Hz, 1H), 3.95−3.92 (m, 1H), 3.72−3.69 (m, 1H), 3.60 (dd, *J* = 5.8, 11.3 Hz, 1H), 2.58 (s, 1H), 2.35 (t, *J* = 7.4 Hz, 2H), 2.16 (s, 1H) 2.04−1.99 (m, 4H), 1.65−1.59 (m, 2H), 1.26 (br, 32H), 0.88 (t, *J* = 6.6 Hz, 3H); ^{13}C NMR (400 MHz, $CDCl_3$) δ 174.5, 130.0, 130.0, 70.4, 65.3, 63.5, 34.3, 32.1, 29.9, 29.8, 29.8, 29.7, 29.7, 29.7, 29.6, 29.5 (3C), 29.4 (2C), 29.3 (2C), 27.4 (2C), 25.1, 22.8, 14.3; HRMS-ESI: $[M + H]^+$ calcd for $C_{27}H_{52}O_4$, 441.3938; found, 441.3942.

^{1}H NMR spectra for compound **1a:**

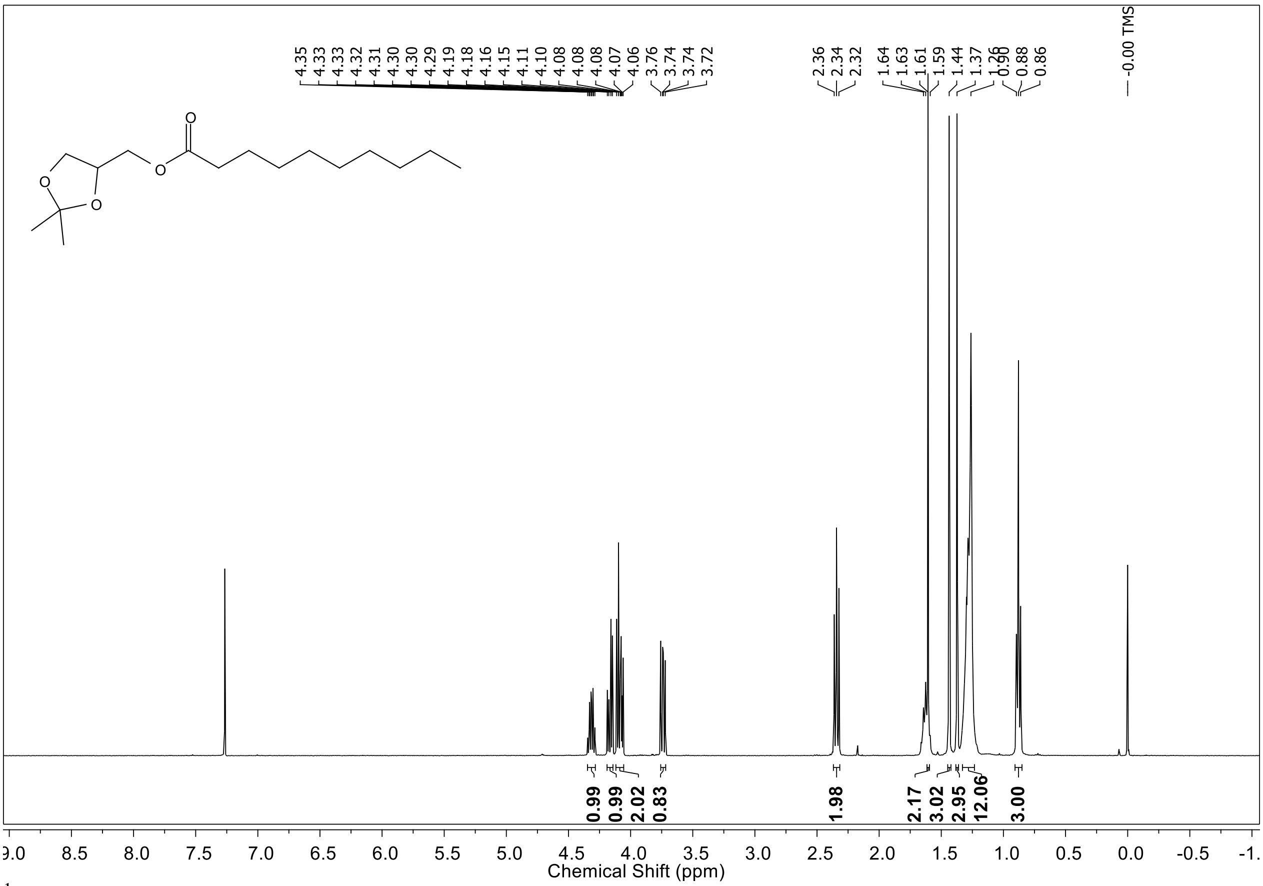

^{1}H NMR spectra for compound **1a':**

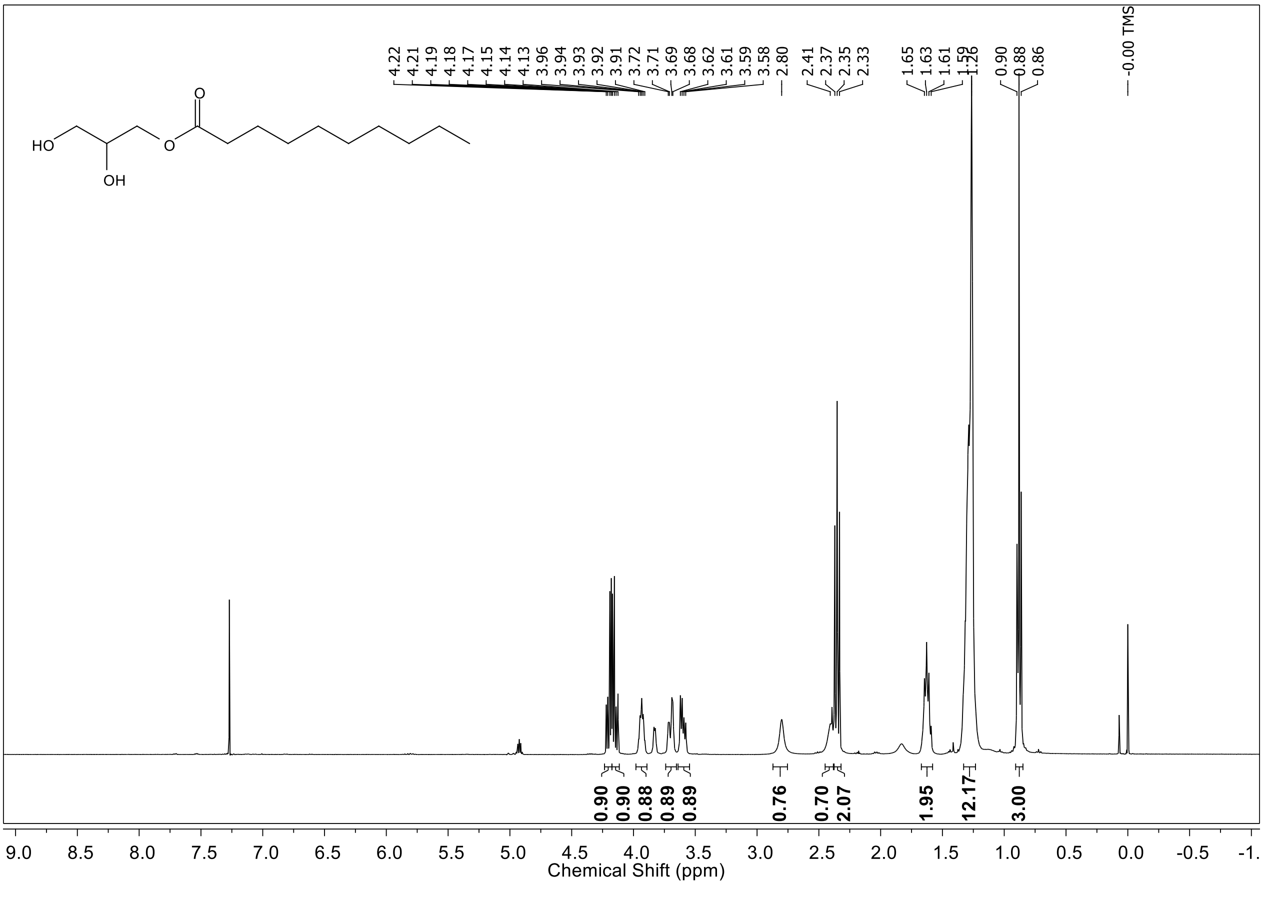

^{13}C NMR spectra for compound **1a’:**

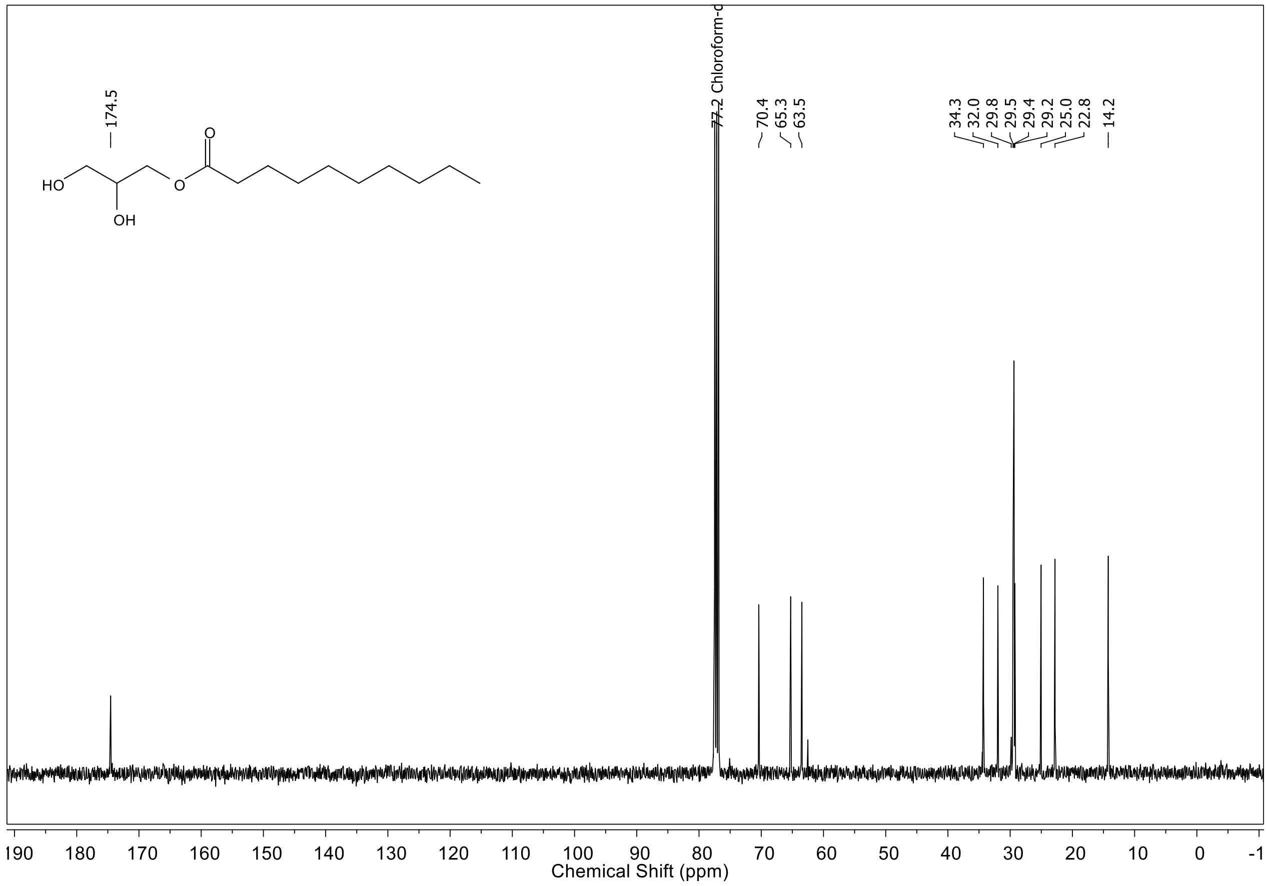

^{1}H NMR spectra for compound **1b:**

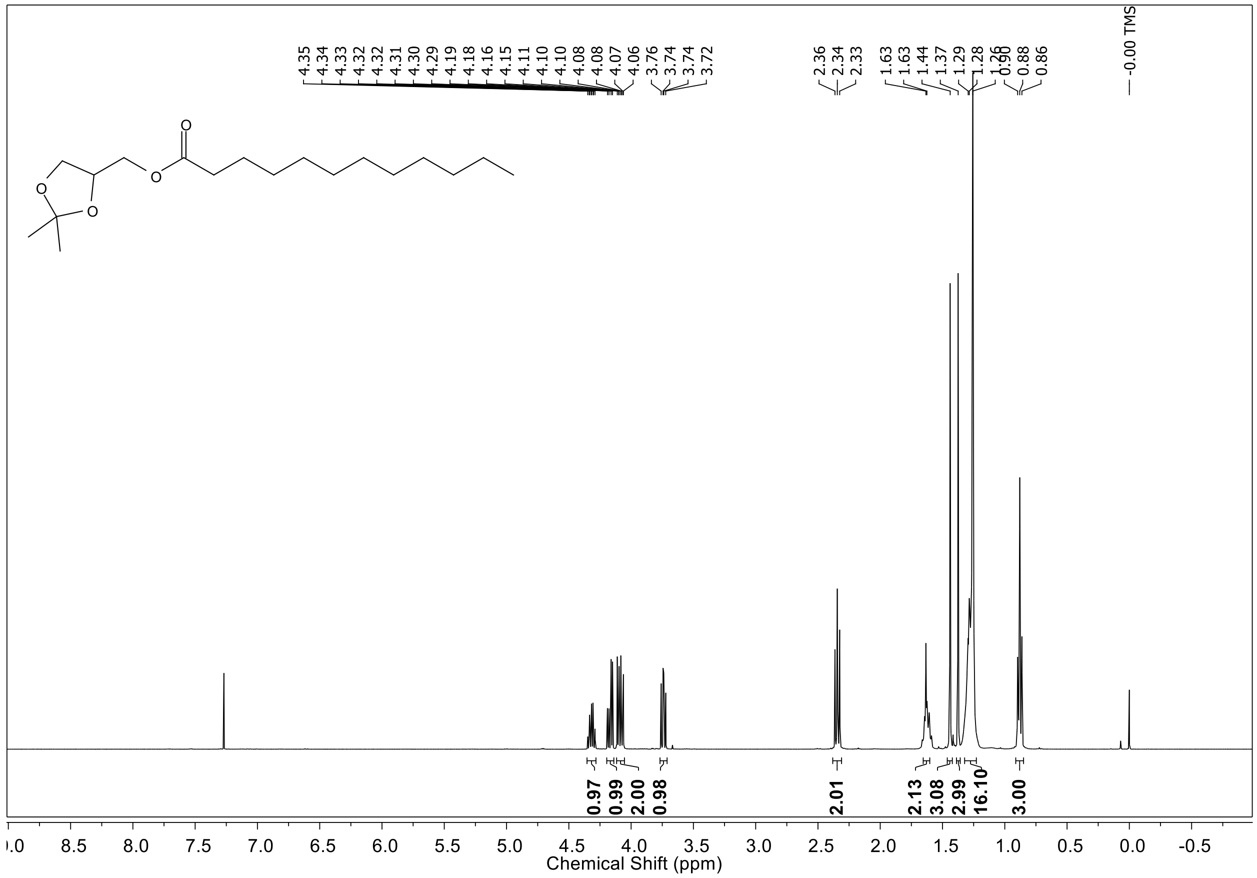

^{1}H NMR spectra for compound **1b'**:

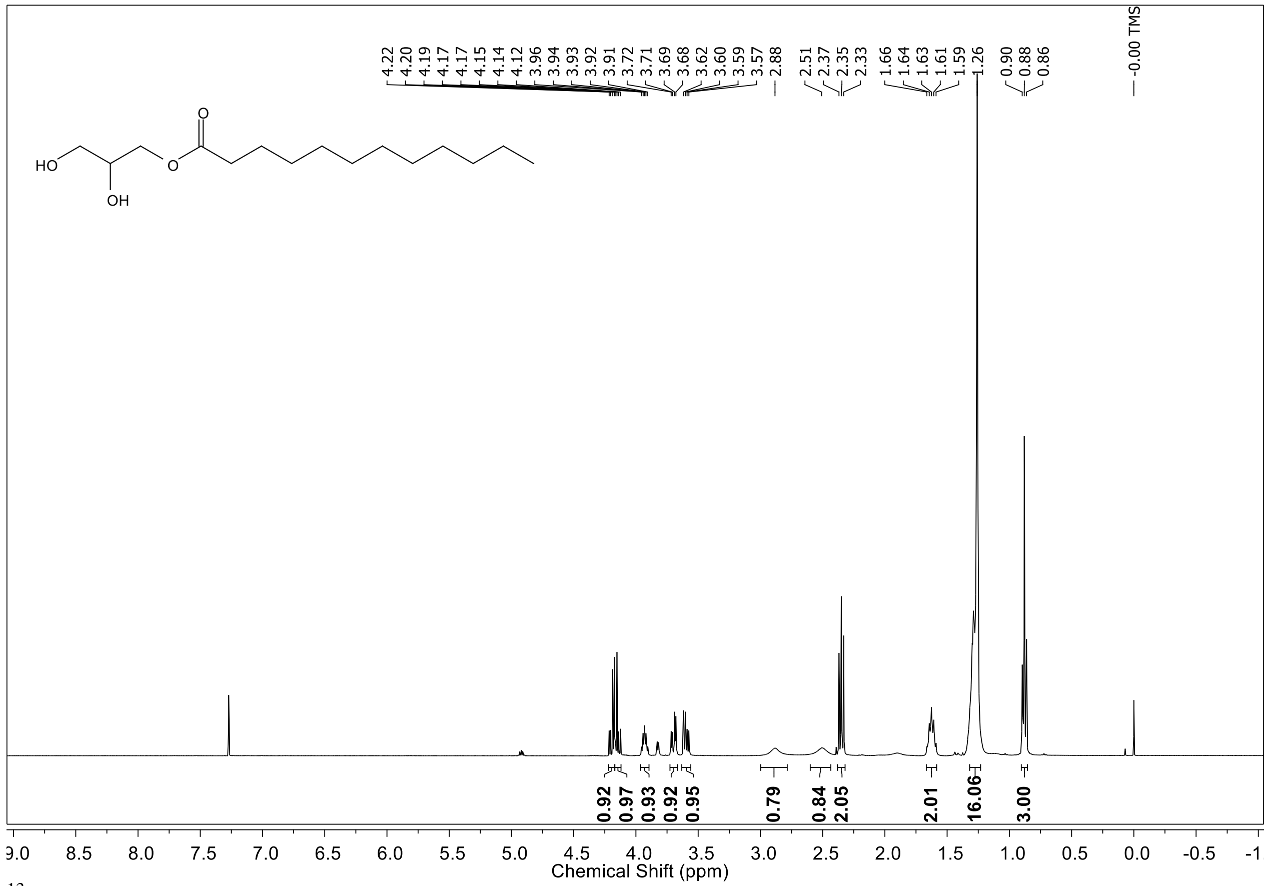

^{13}C NMR spectra for compound **1b'**:

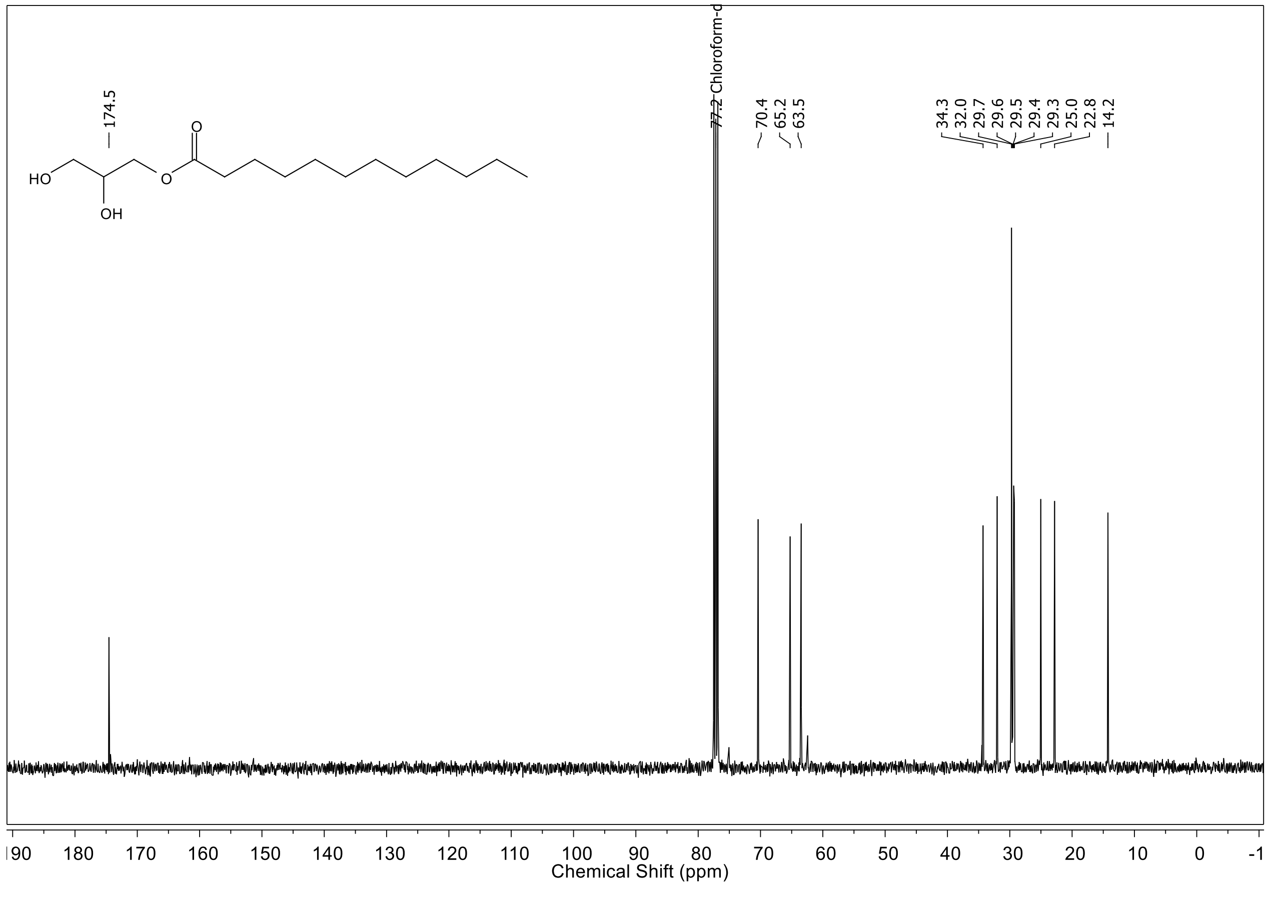

^{1}H NMR spectra for compound **1c:**

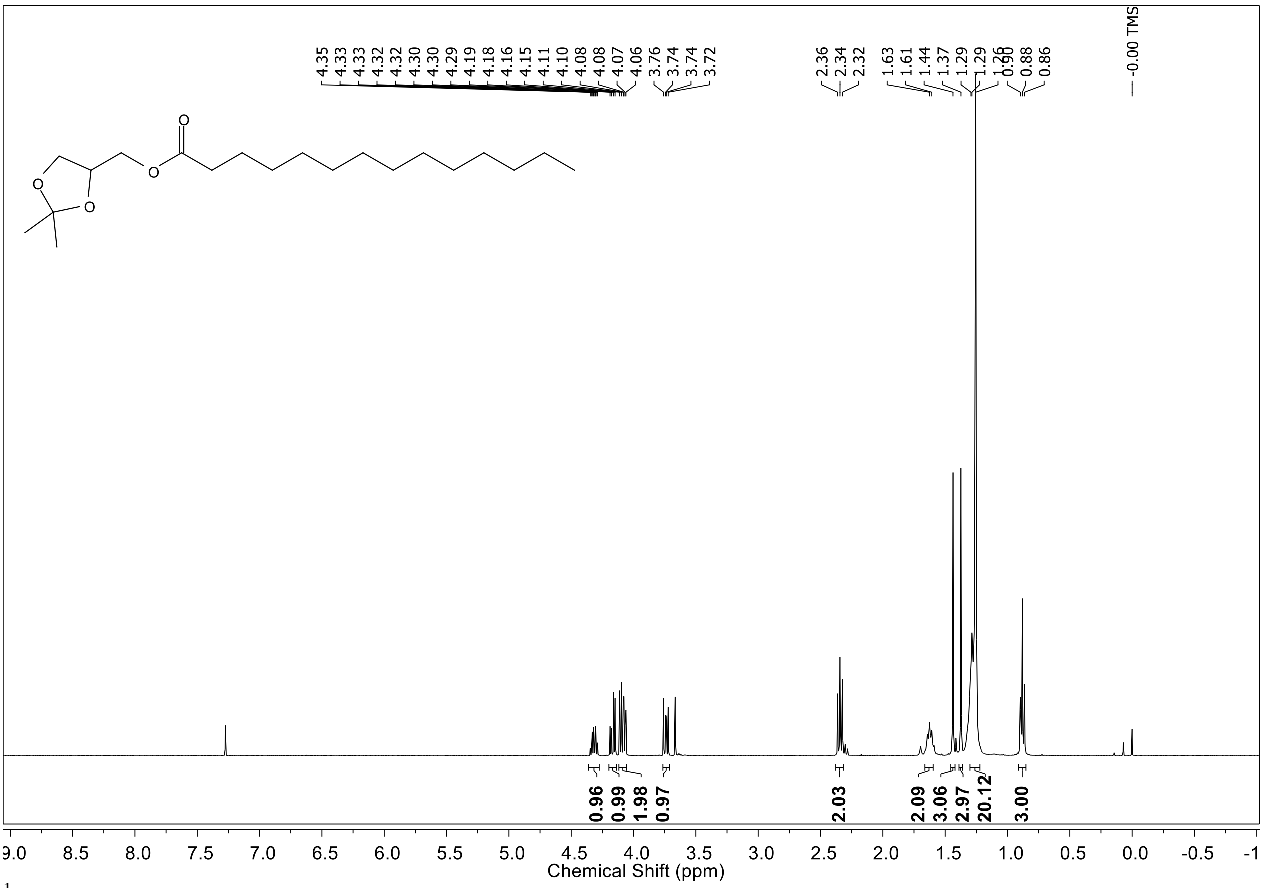

^{1}H NMR spectra for compound **1c':**

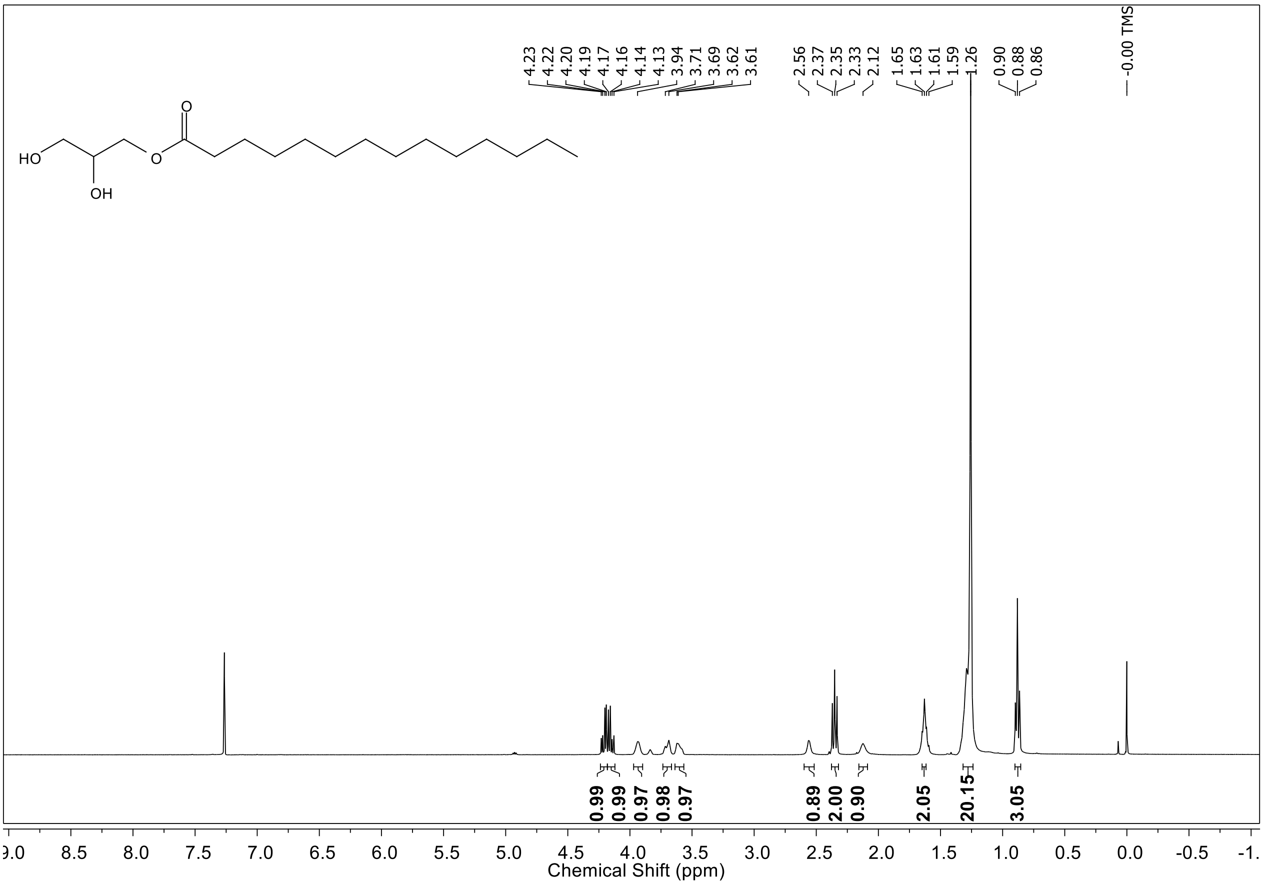

^{13}C NMR spectra for compound **1c'**:

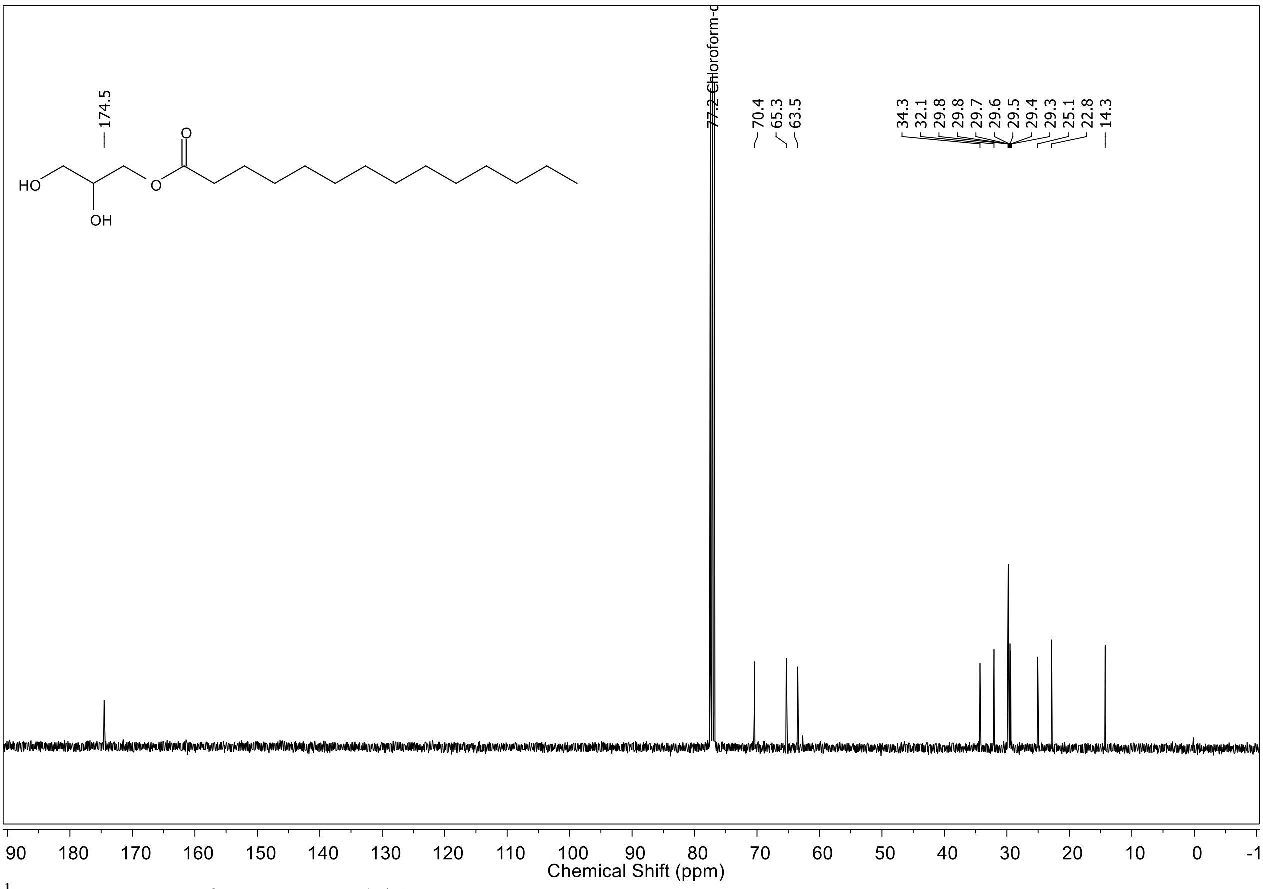

^{1}H NMR spectra for compound **1d**:

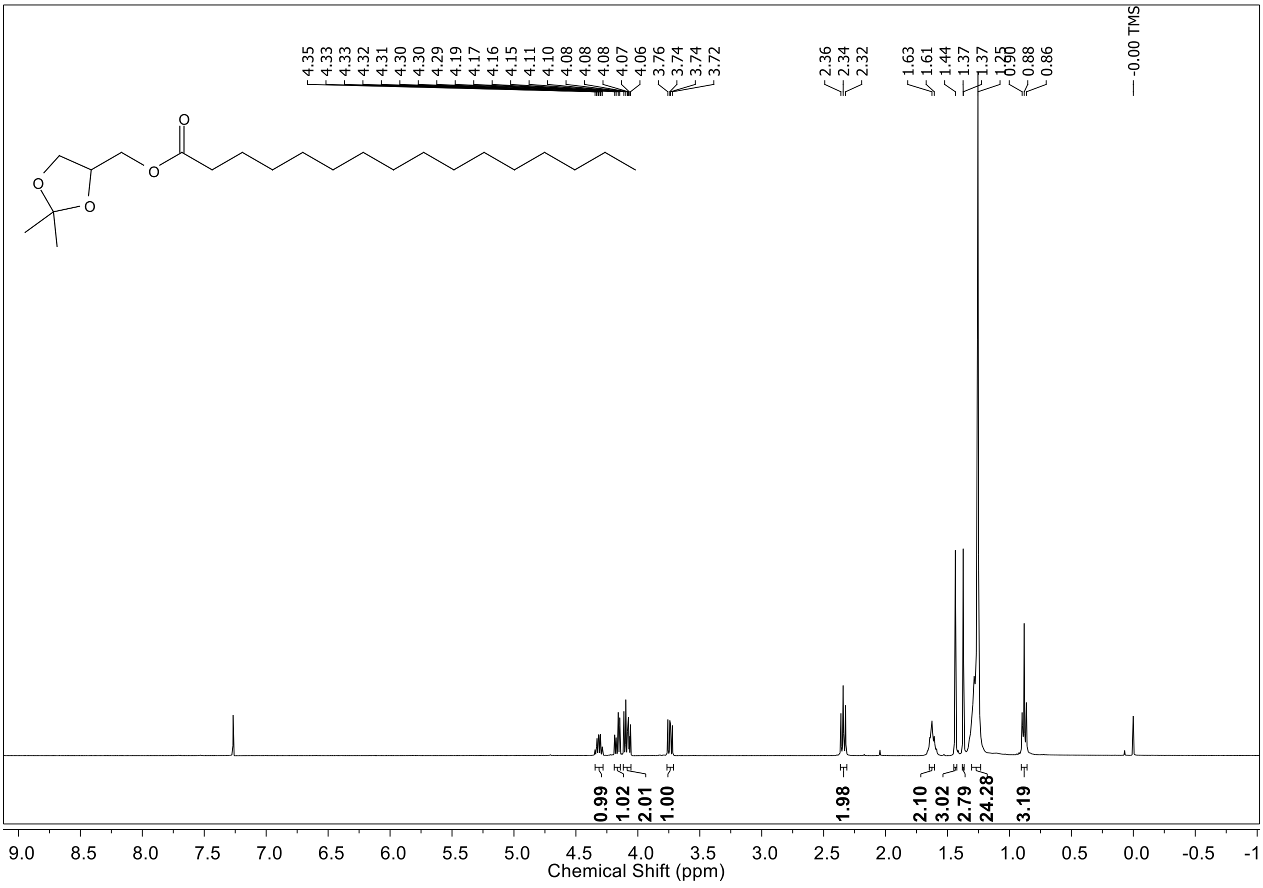

^{1}H NMR spectra for compound **1d'**:

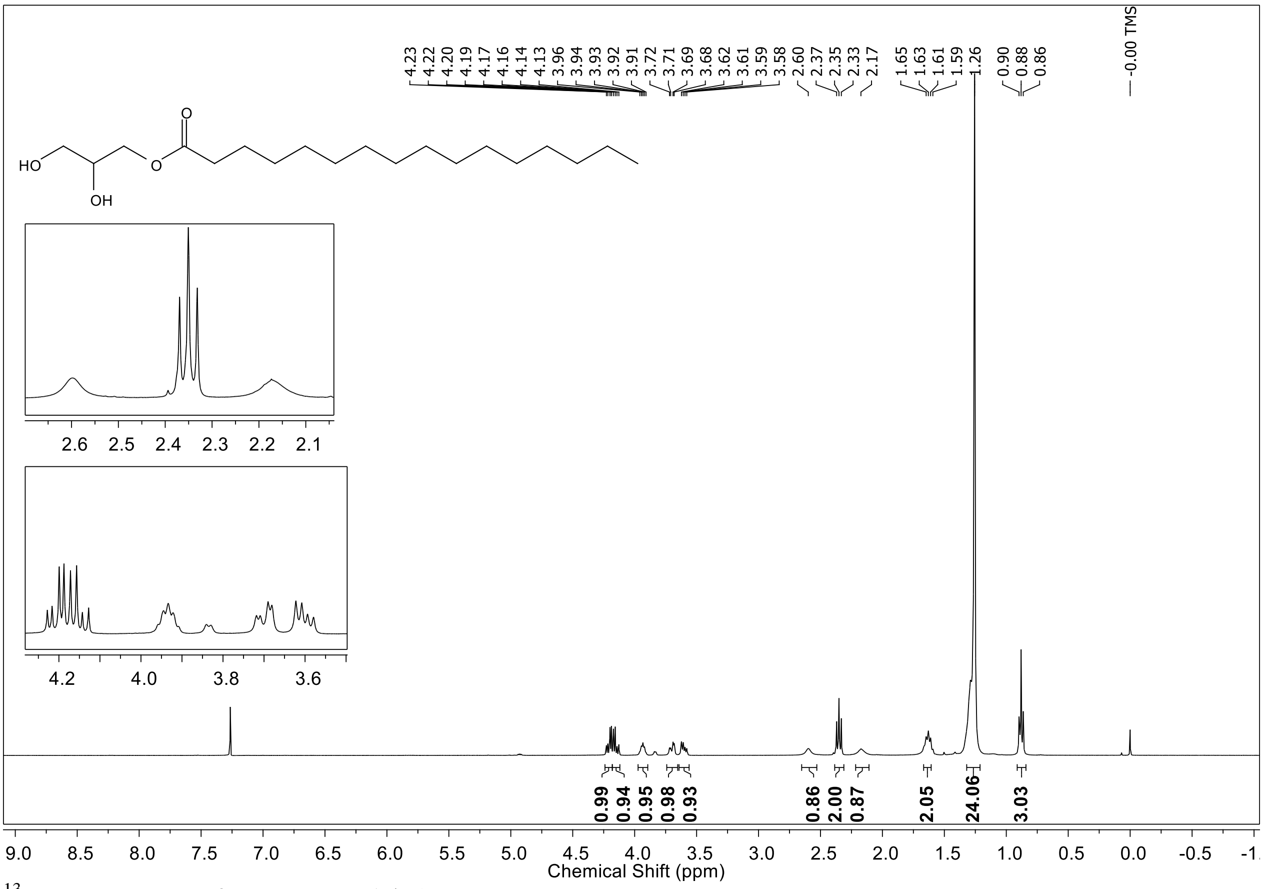

^{13}C NMR spectra for compound **1d'**:

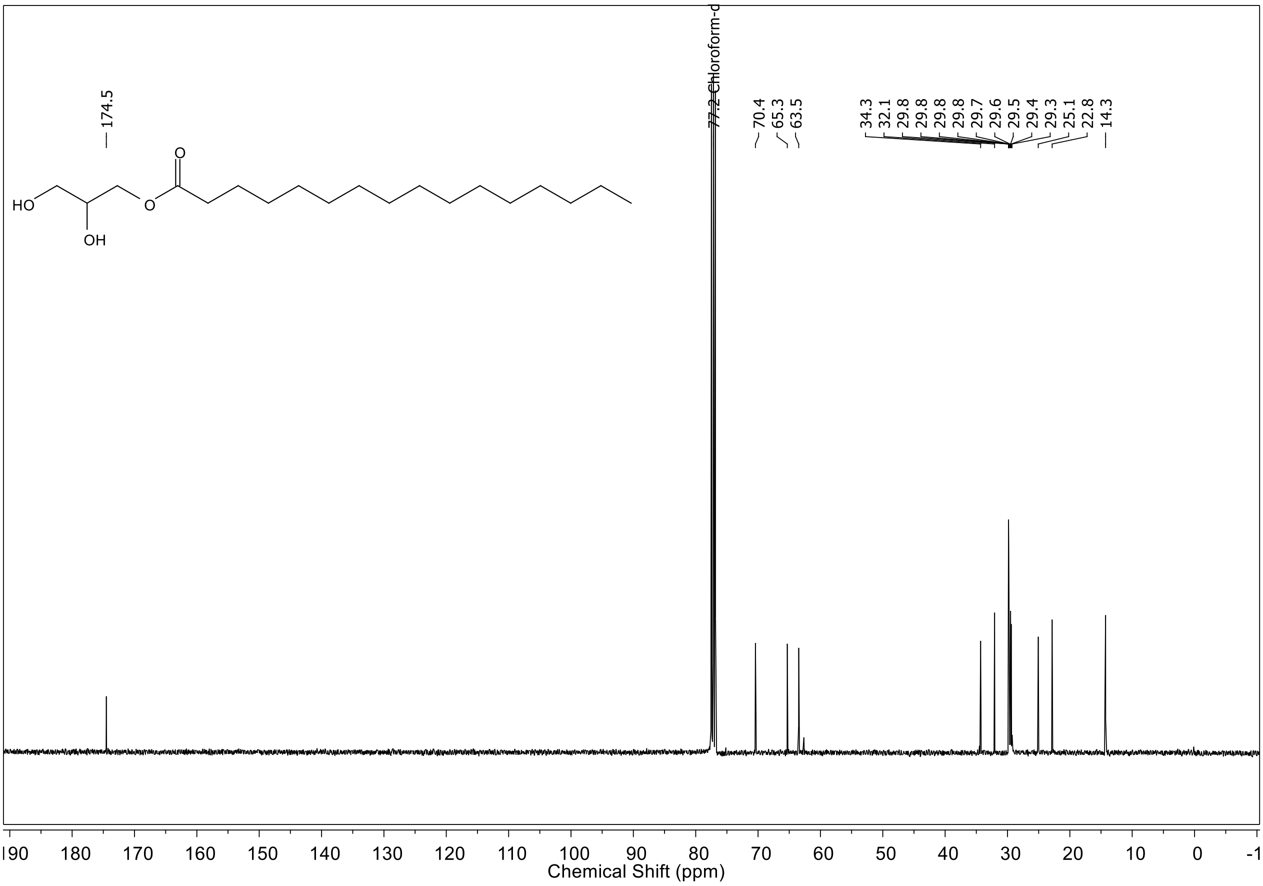

^{1}H NMR spectra for compound **1e:**

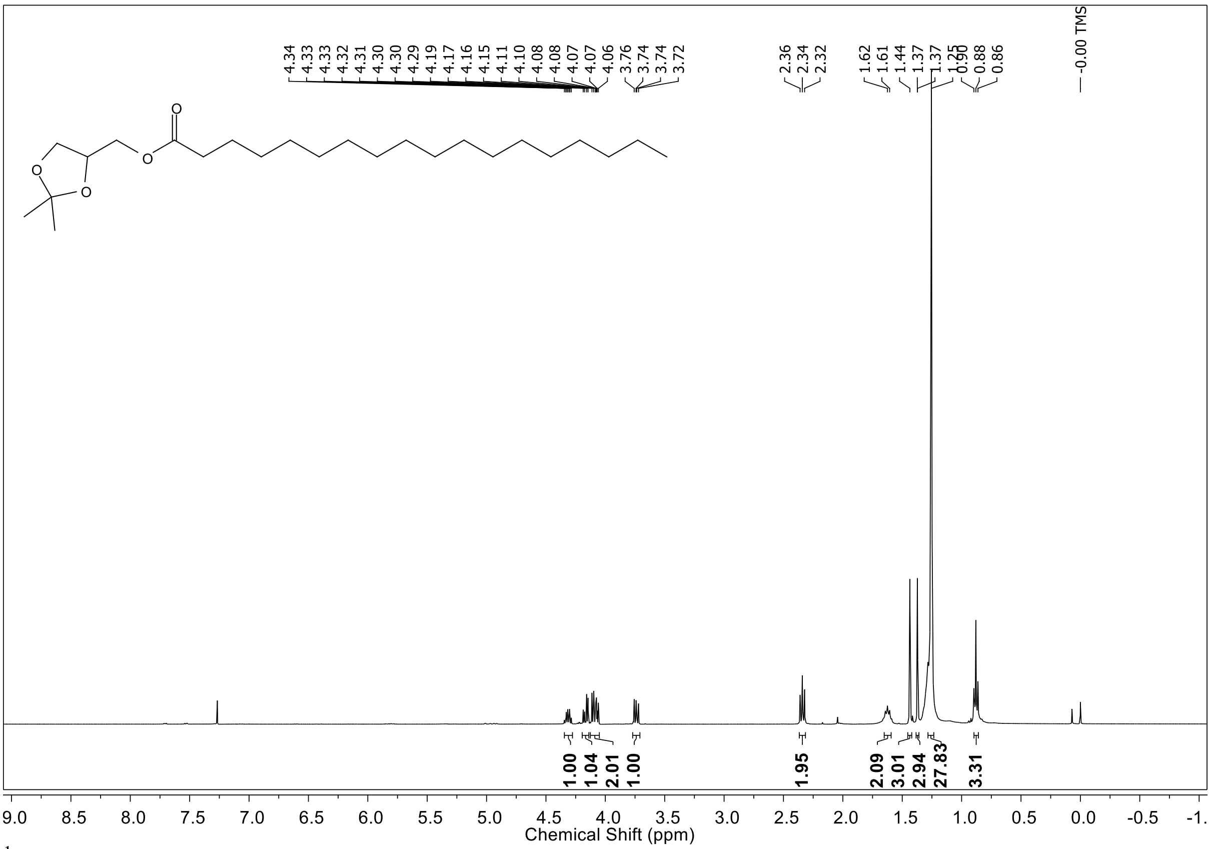

^{1}H NMR spectra for compound **1e'**:

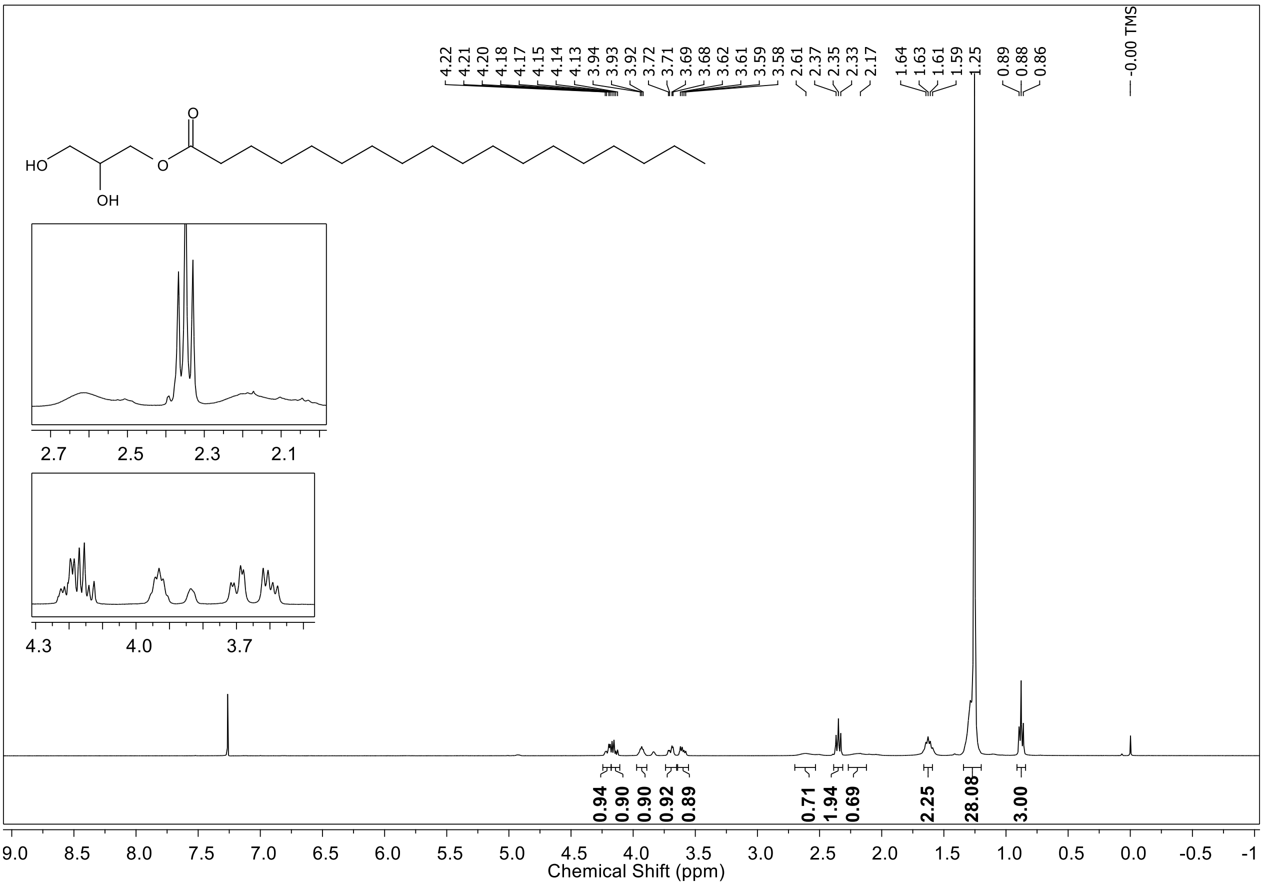

^{13}C NMR spectra for compound **1e'**:

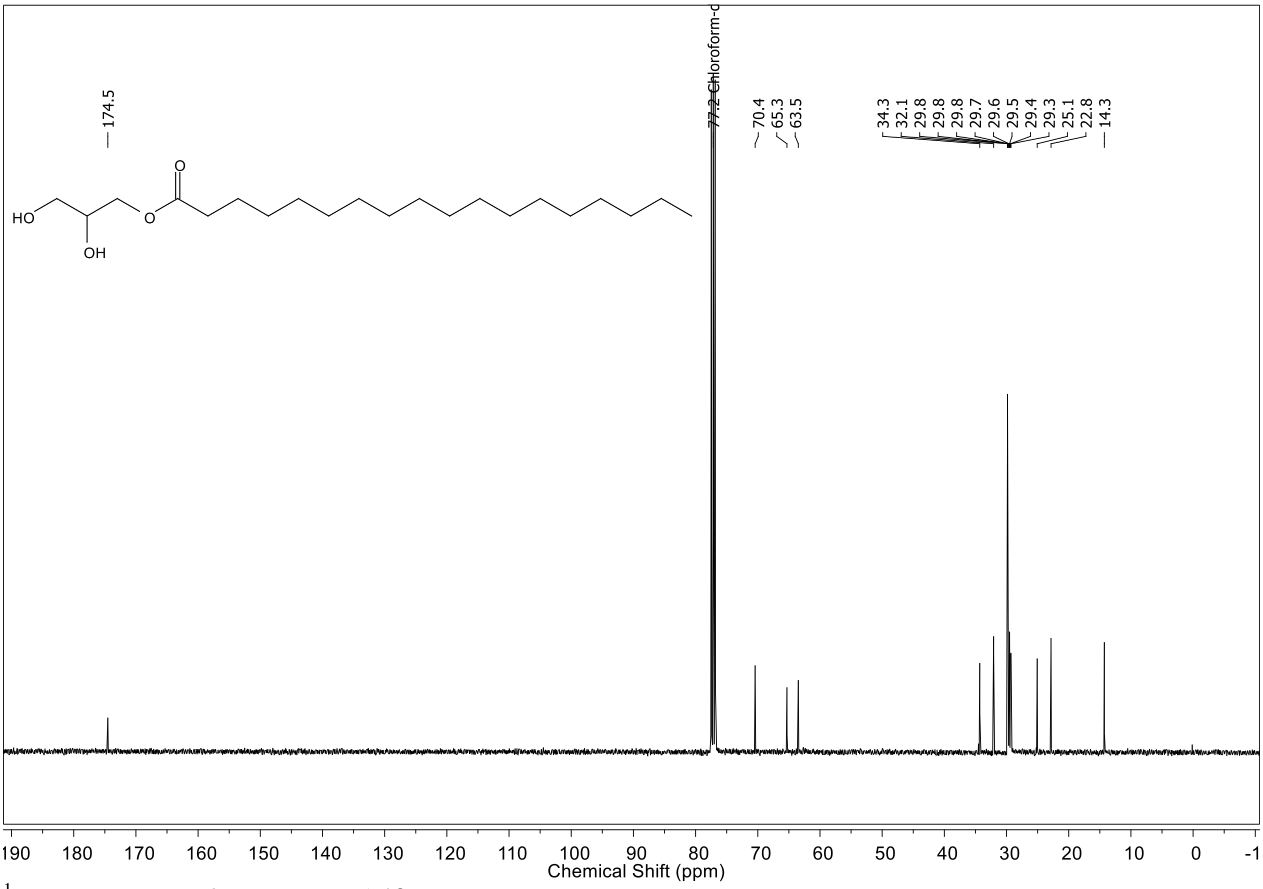

^{1}H NMR spectra for compound **1f**:

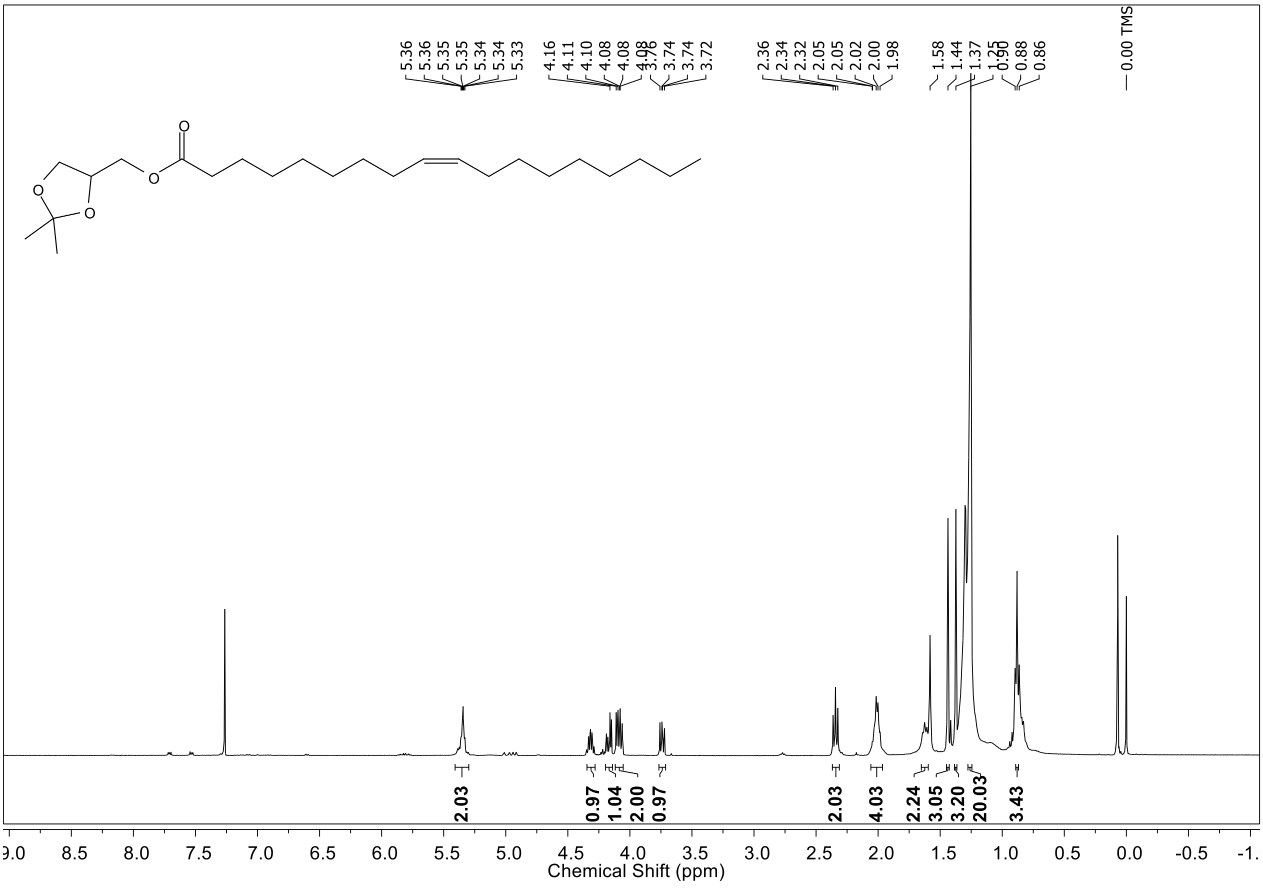

^{1}H NMR spectra for compound **1f'**:

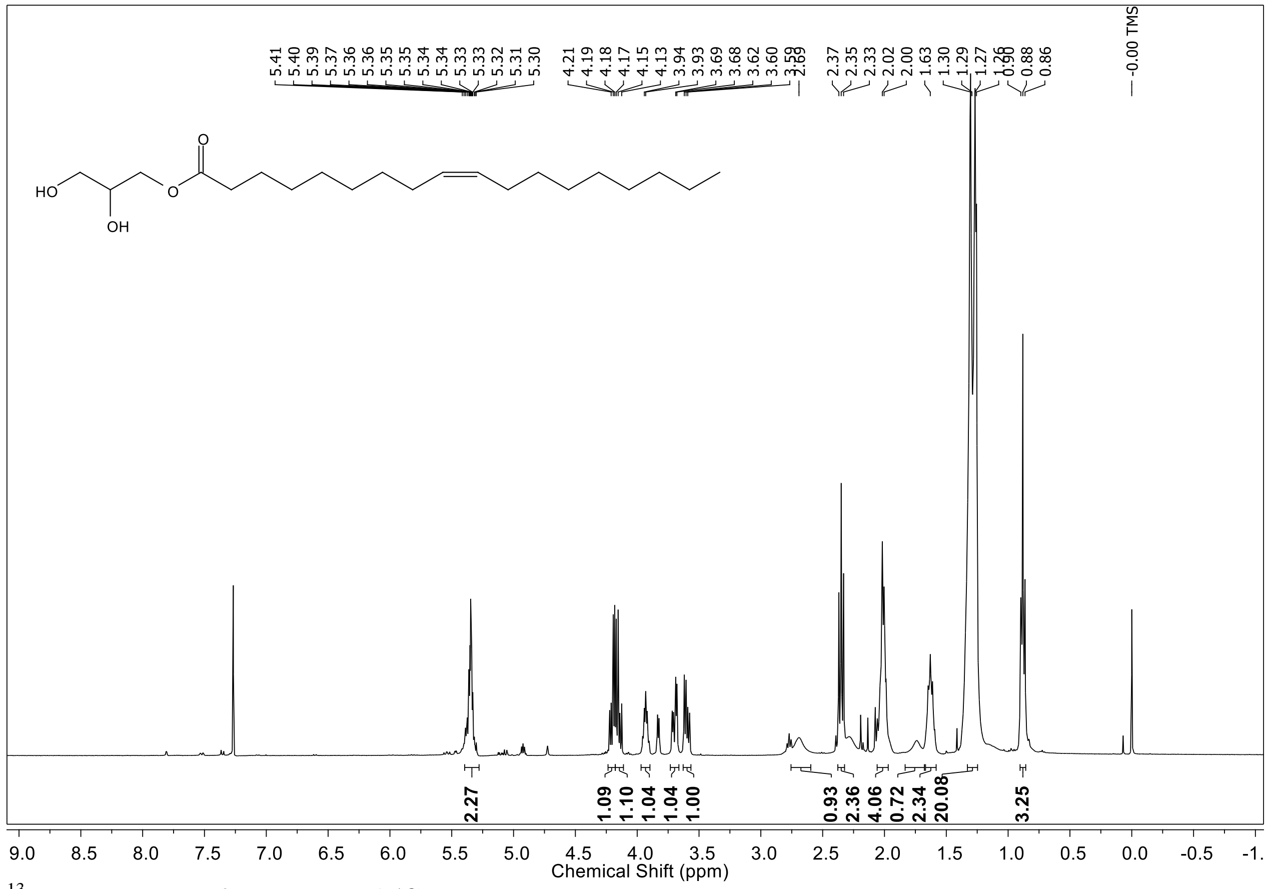

^{13}C NMR spectra for compound **1f'**:

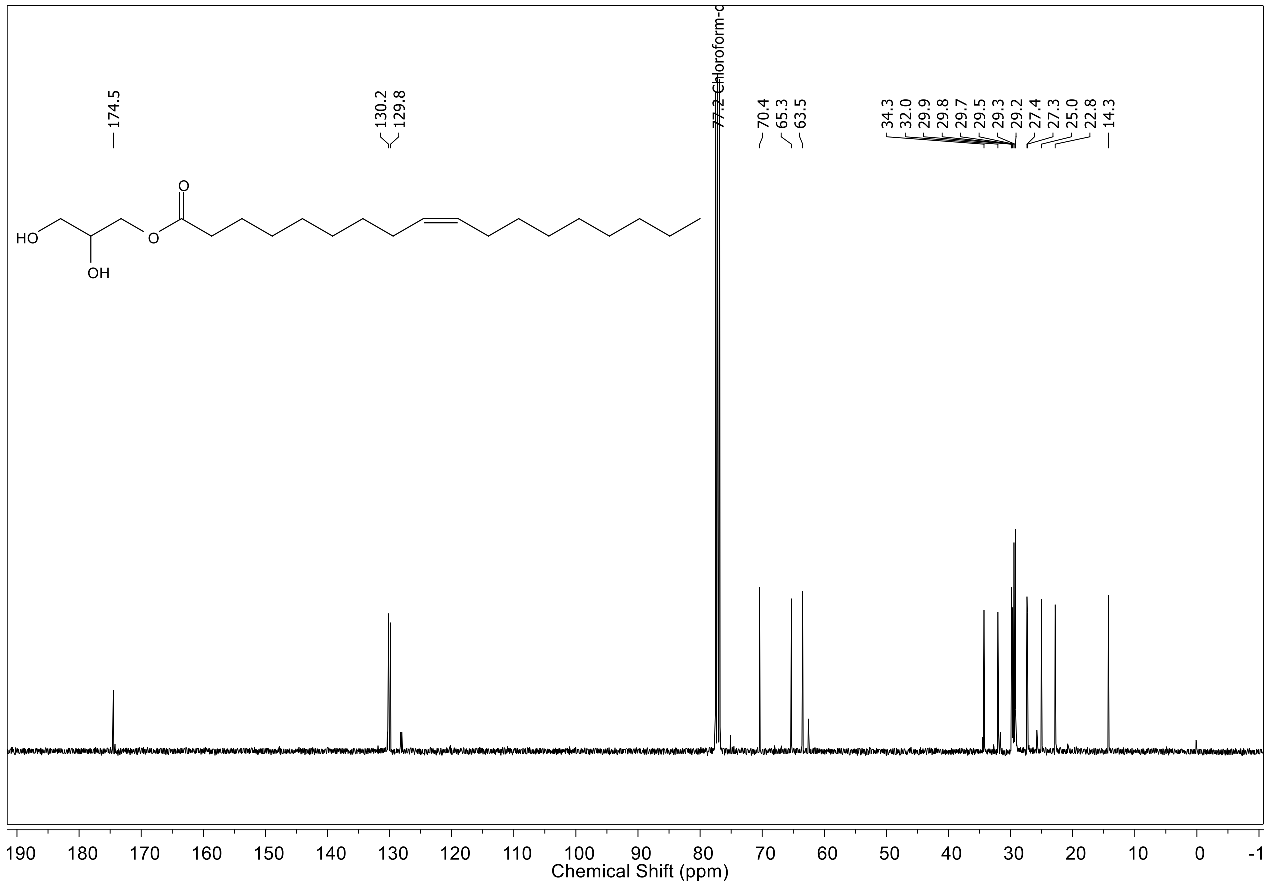

^{1}H NMR spectra for compound **1g:**

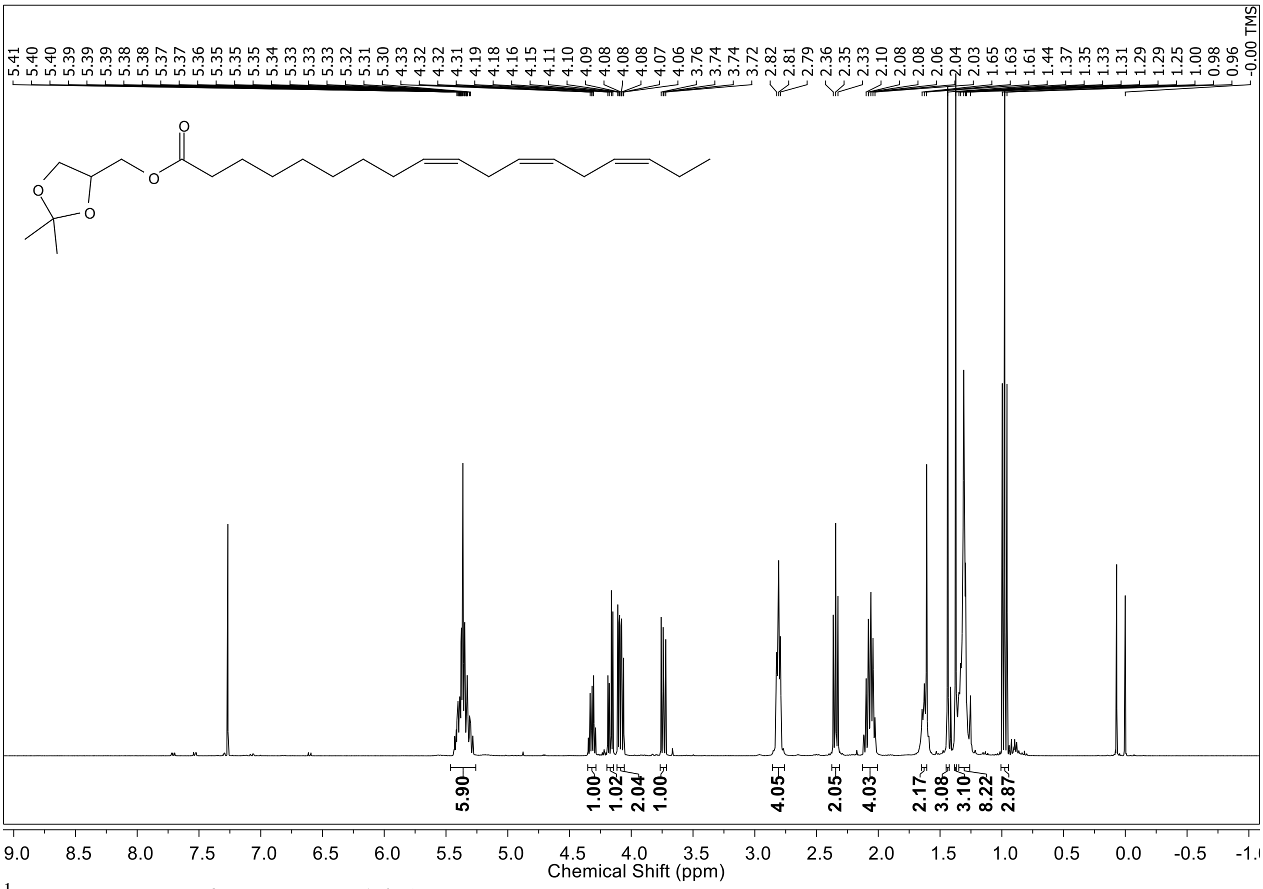

^{1}H NMR spectra for compound **1g':**

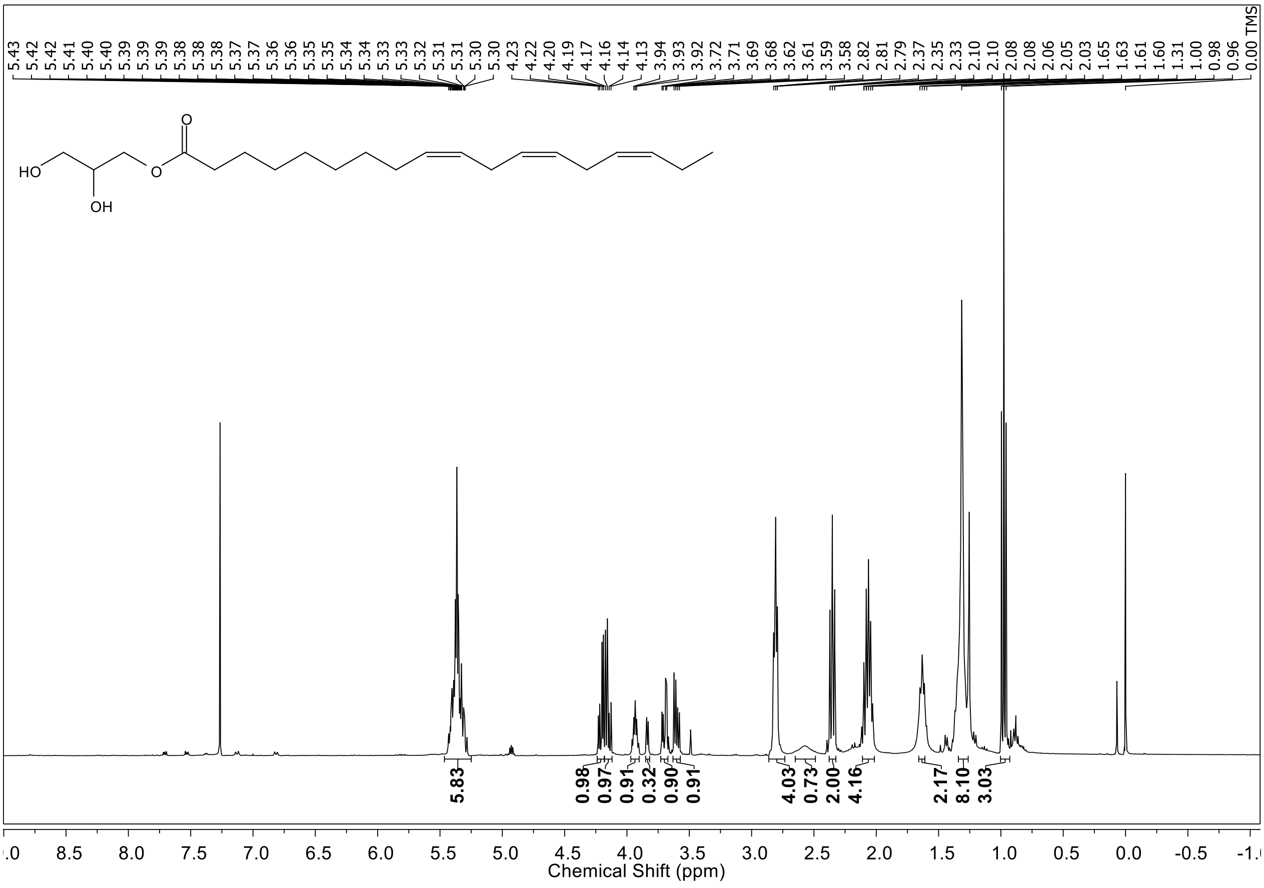

^{13}C NMR spectra for compound **1g'**:

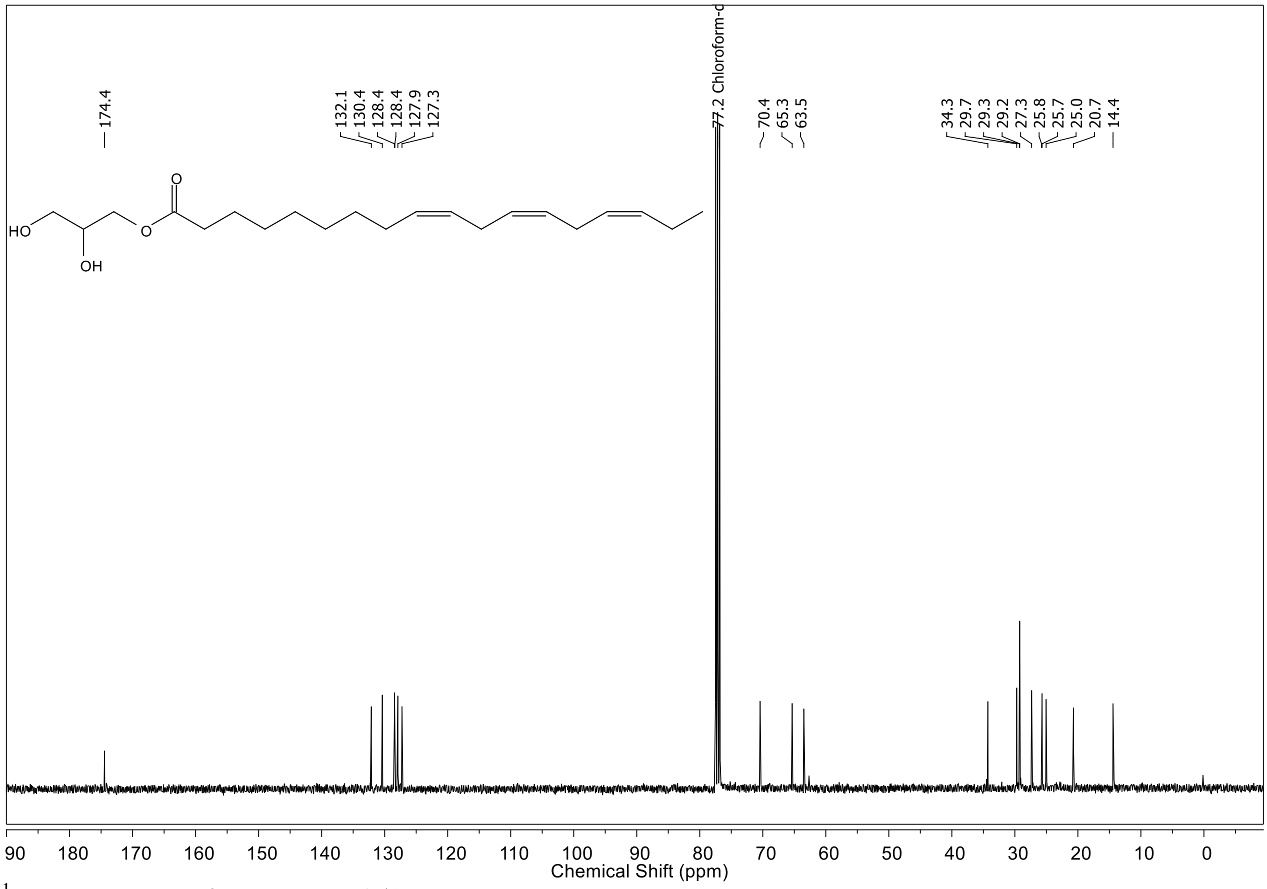

^{1}H NMR spectra for compound **1h:**

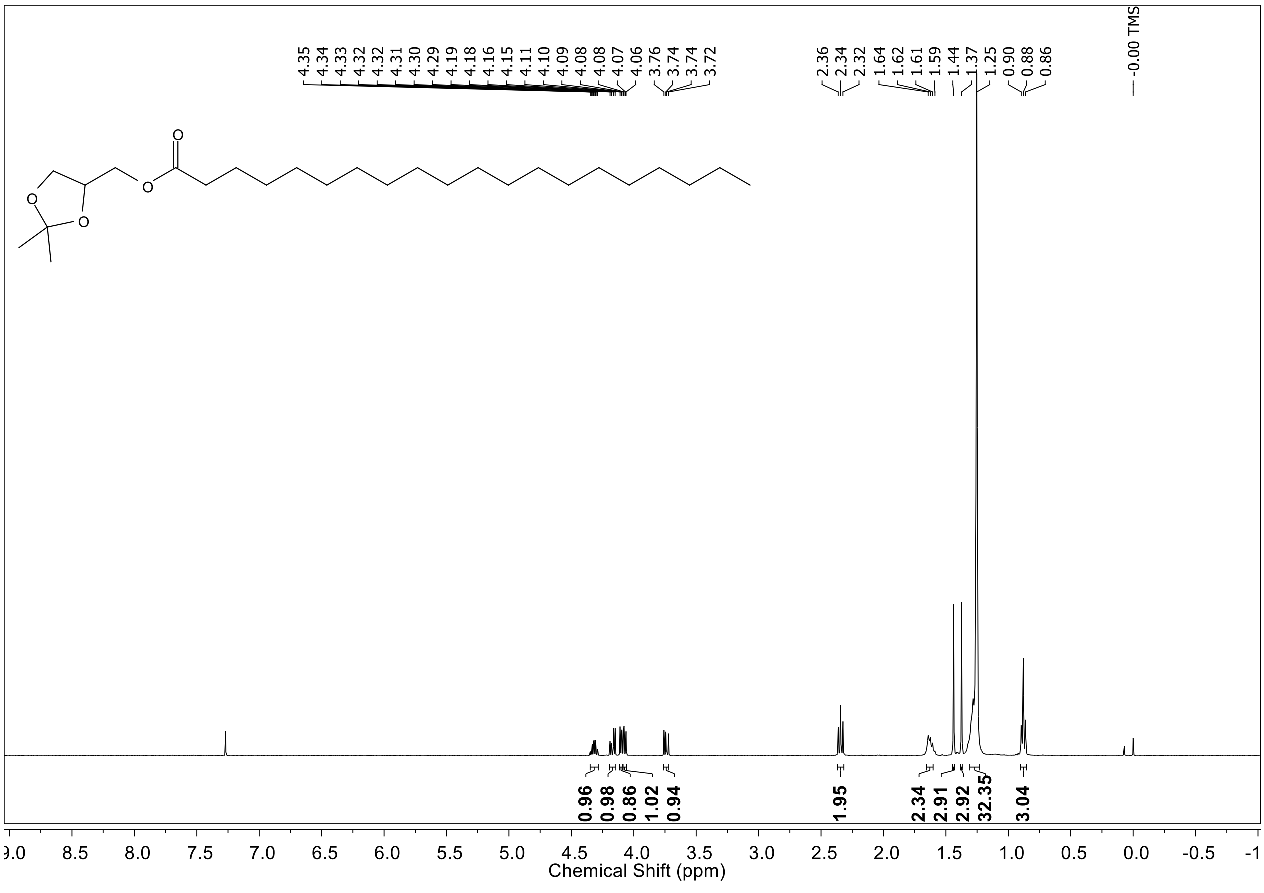

^{1}H NMR spectra for compound **1h'**:

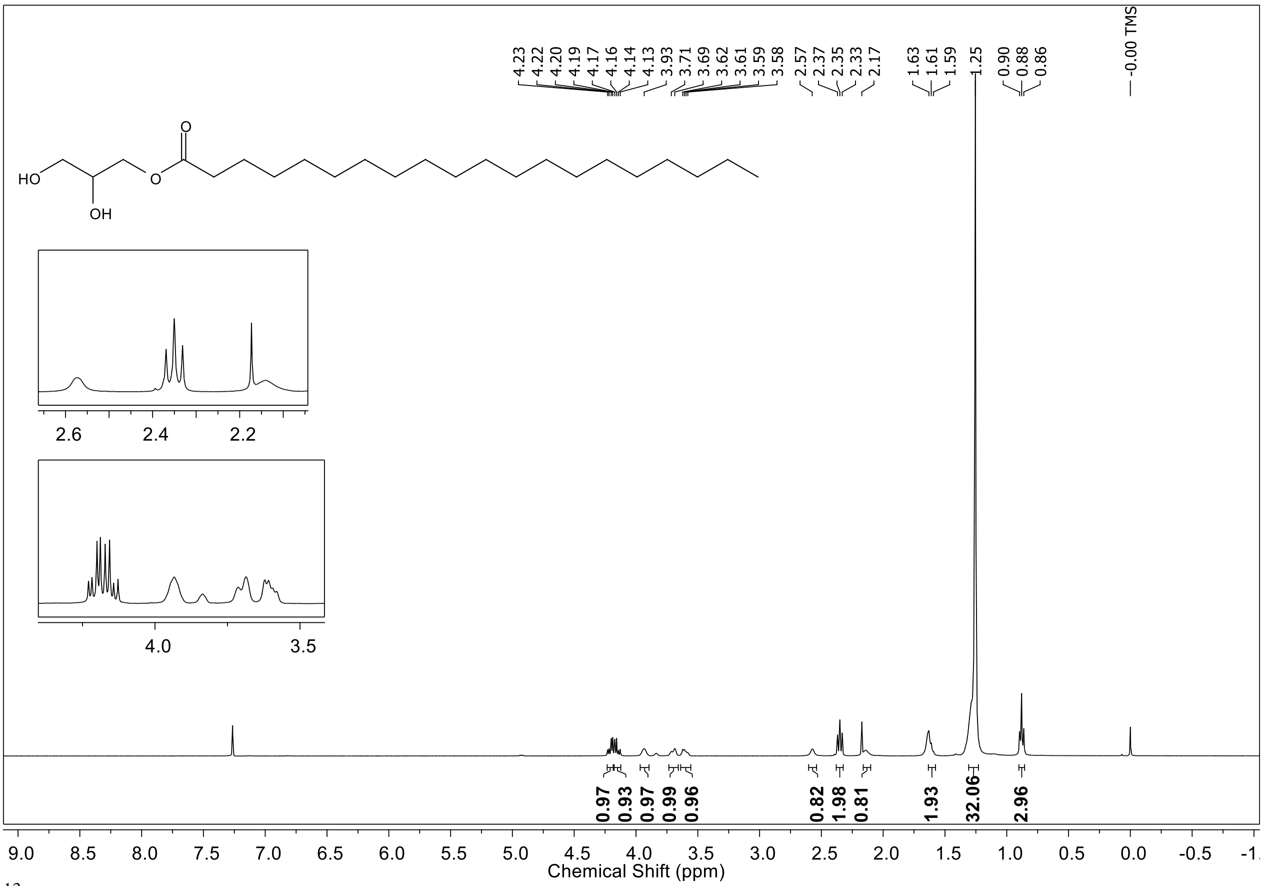

^{13}C NMR spectra for compound **1h'**:

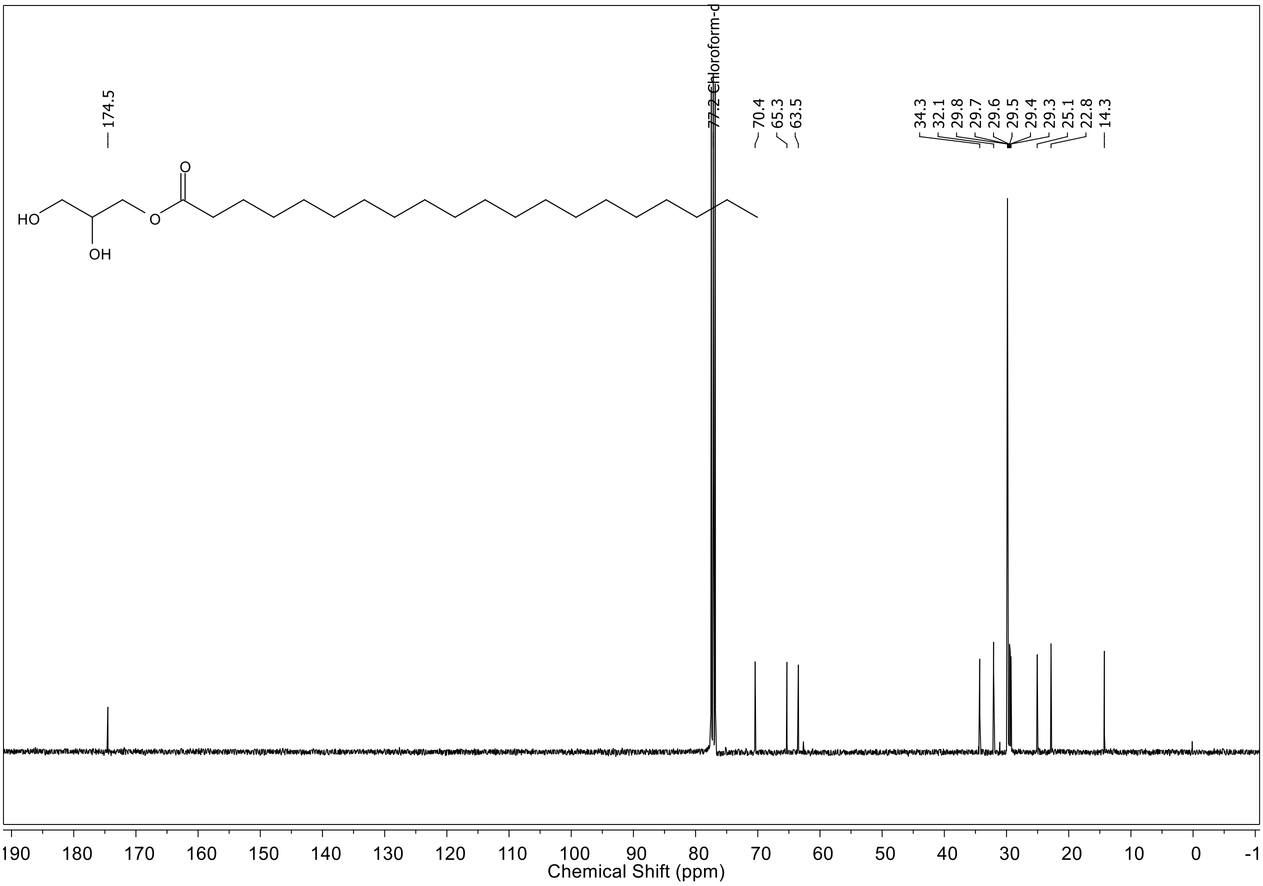

^{1}H NMR spectra for compound **1i:**

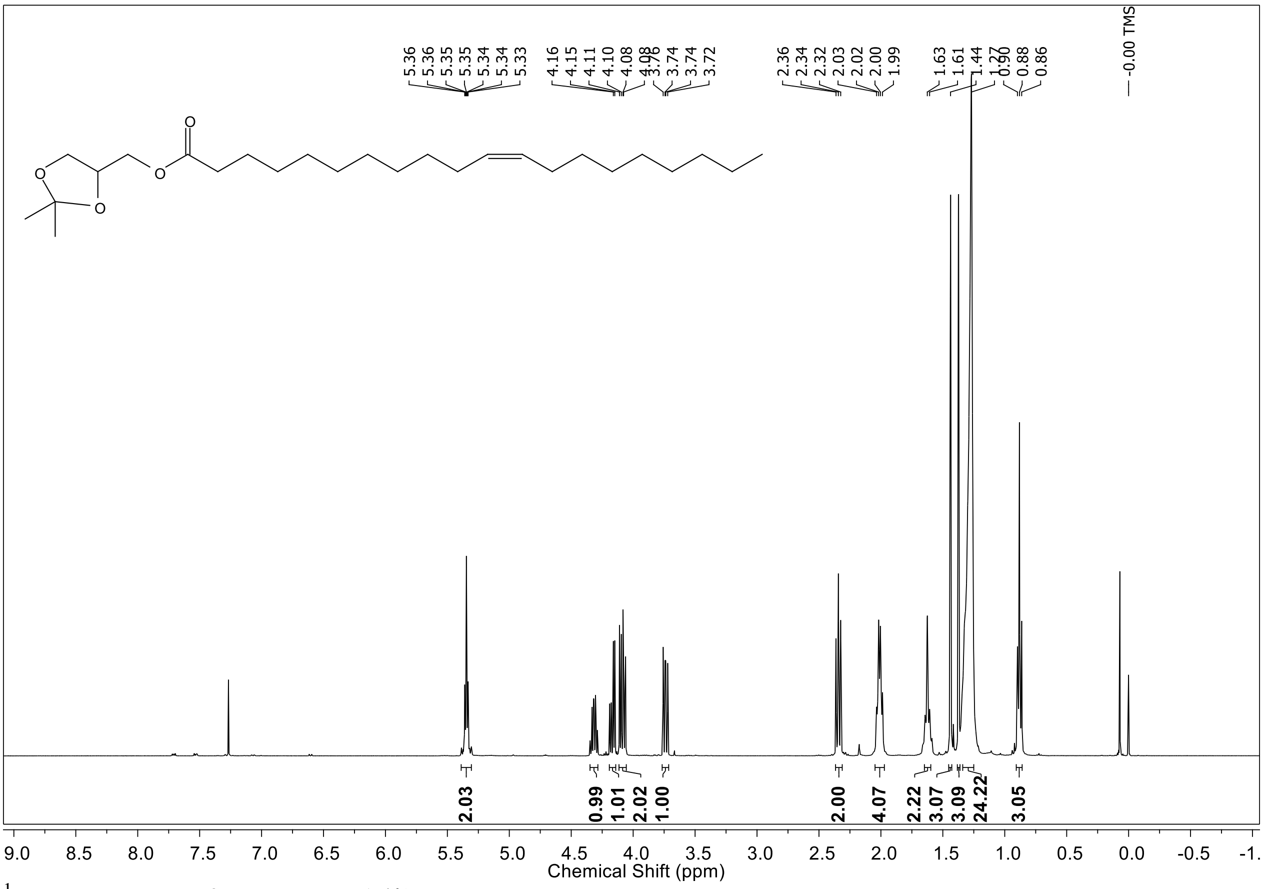

^{1}H NMR spectra for compound **1i’:**

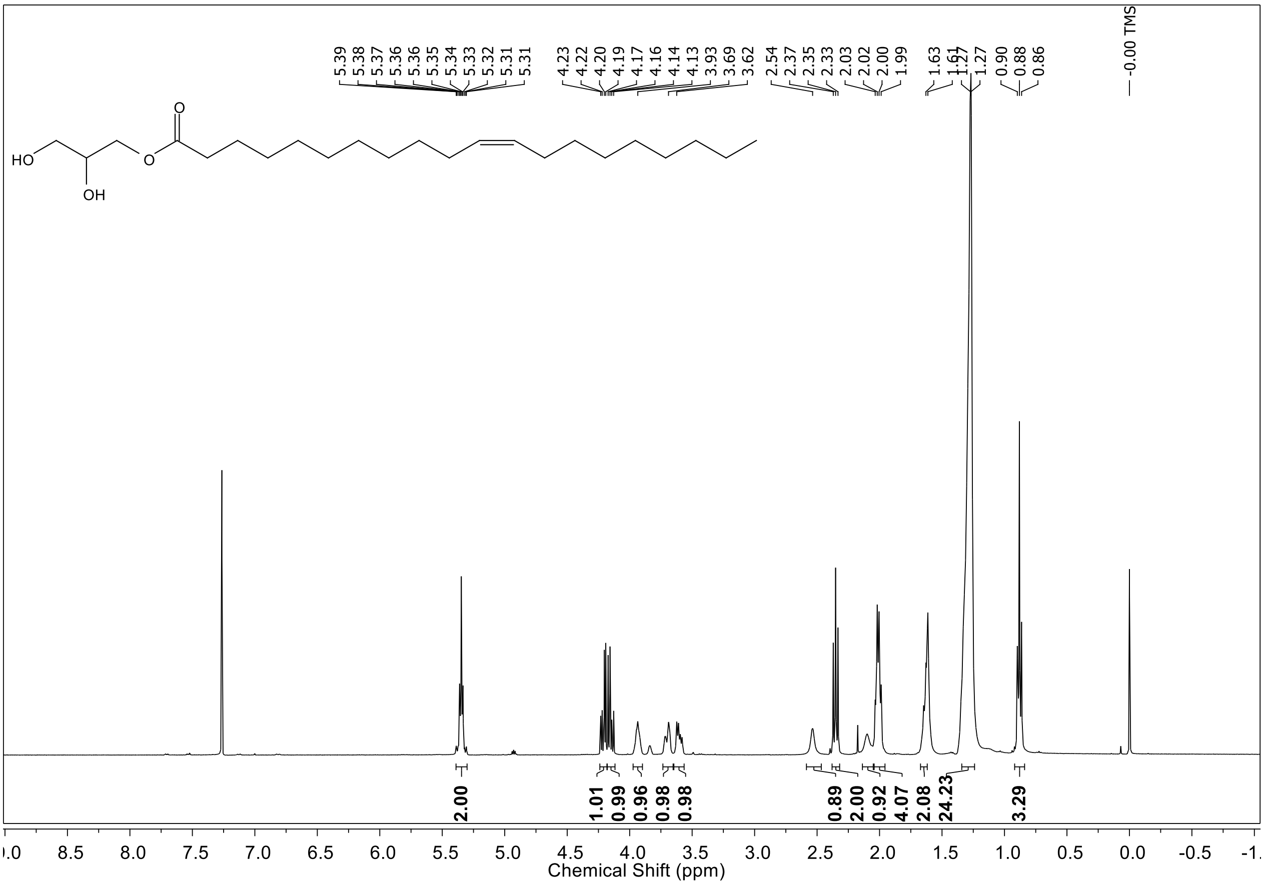

^{13}C NMR spectra for compound **1i'**:

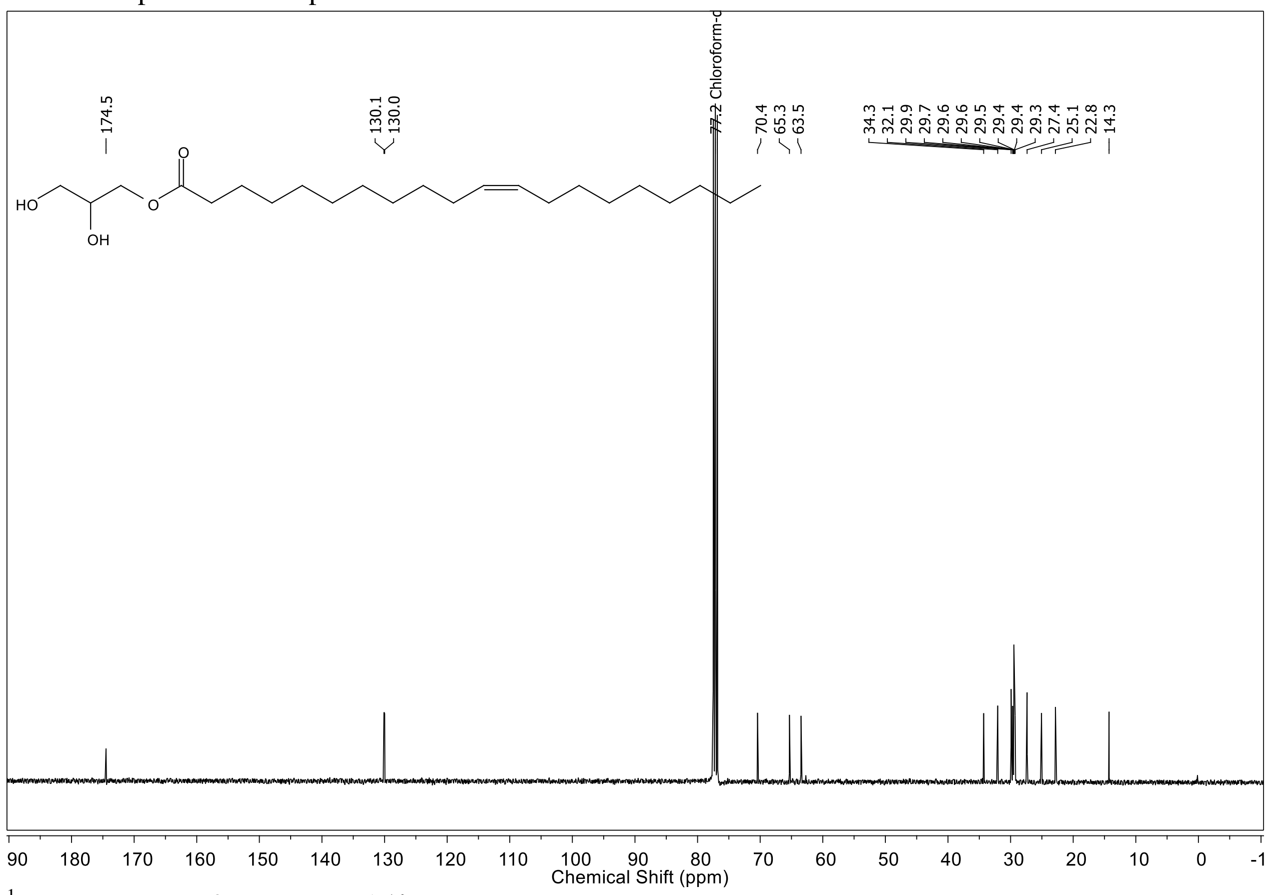

^{1}H NMR spectra for compound **1j**:

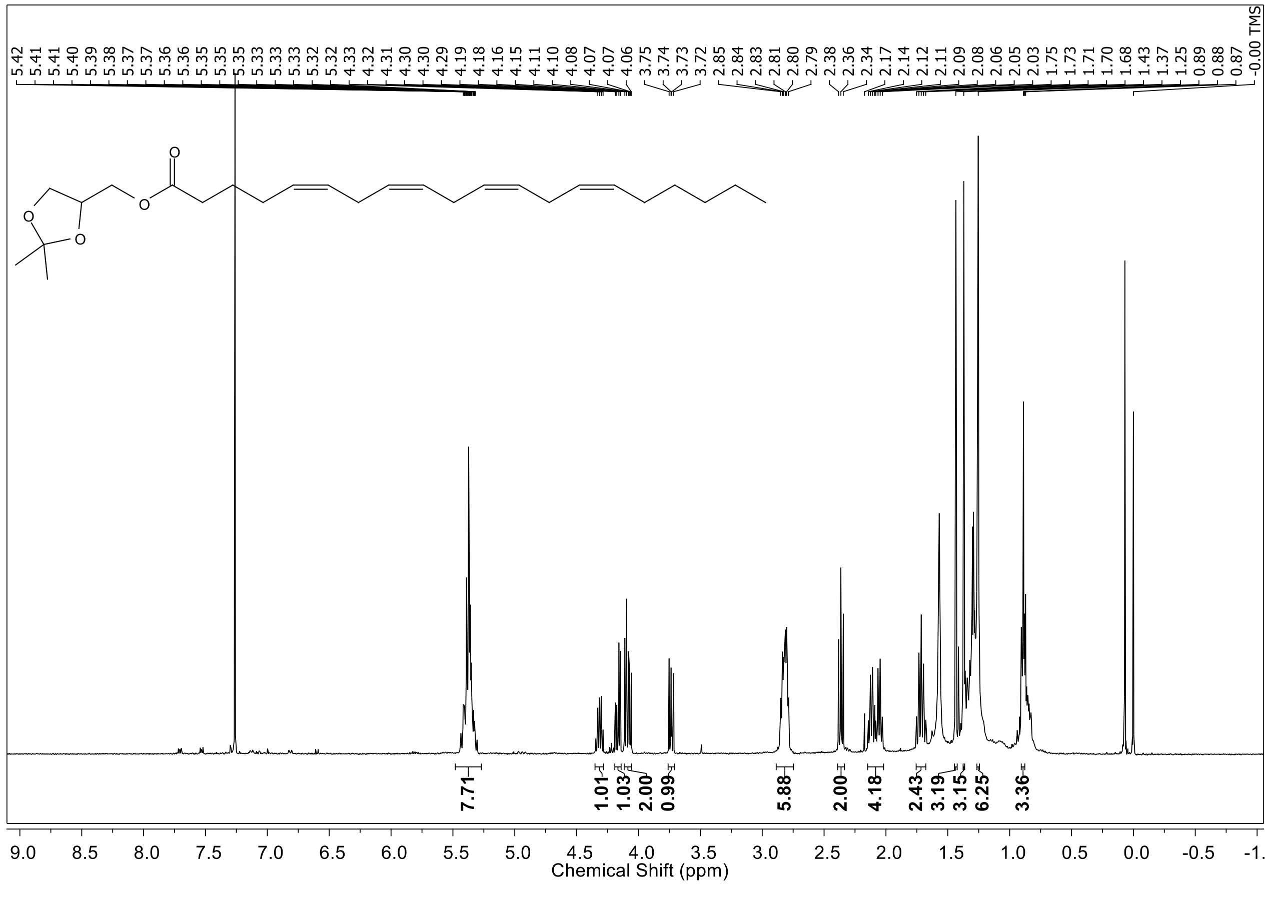

^{1}H NMR spectra for compound **1j'**:

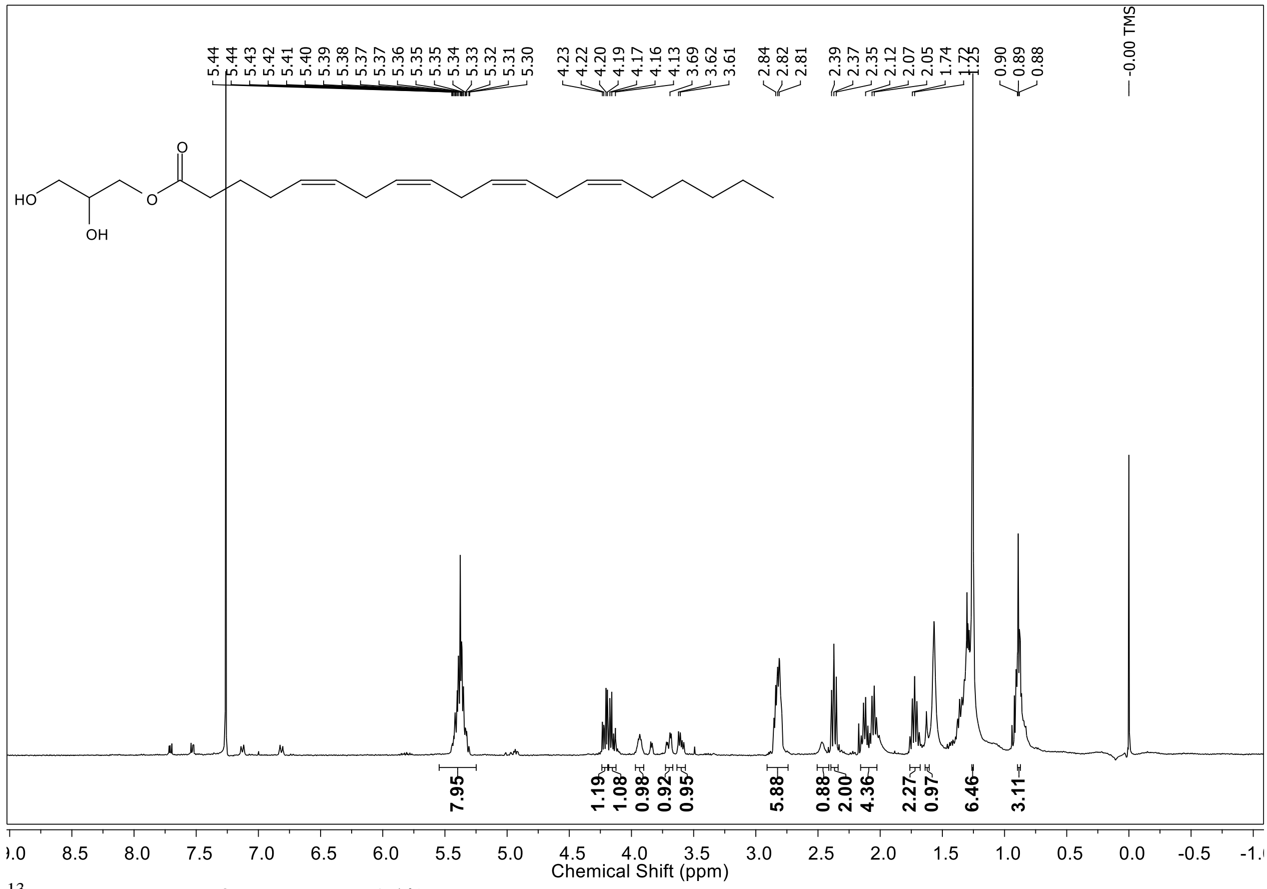

^{13}C NMR spectra for compound **1j'**:

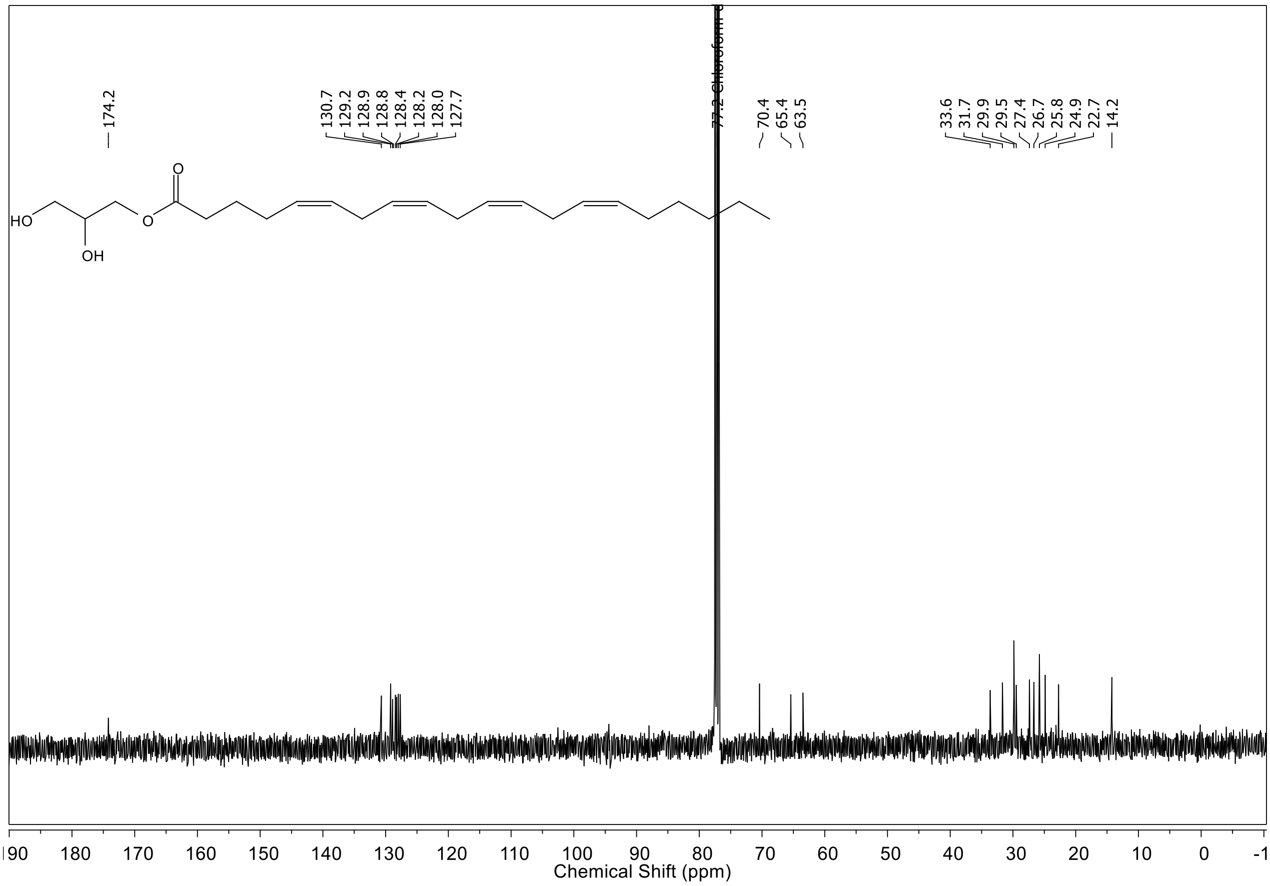

^{1}H NMR spectra for compound **1k:**

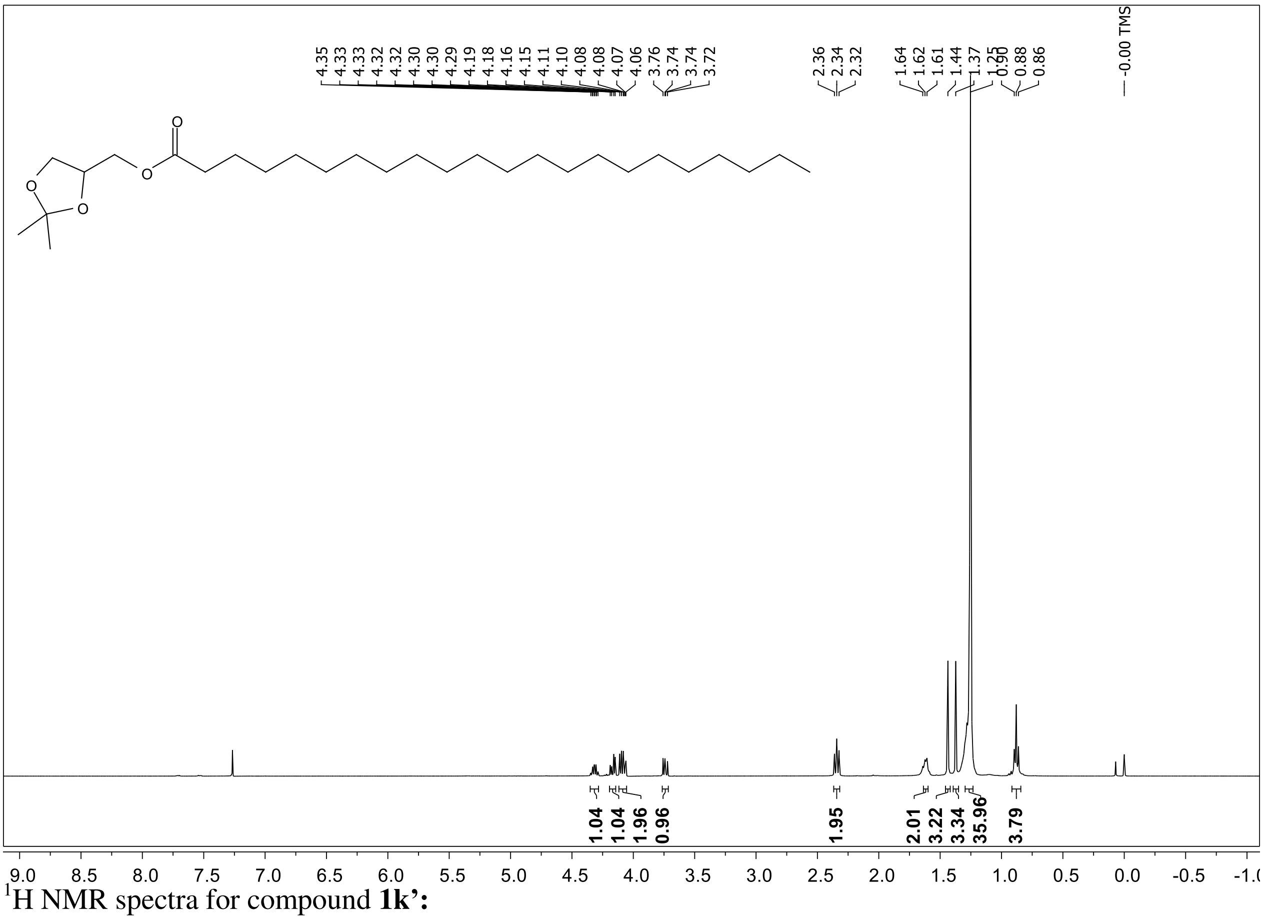

^{1}H NMR spectra for compound **1k':**

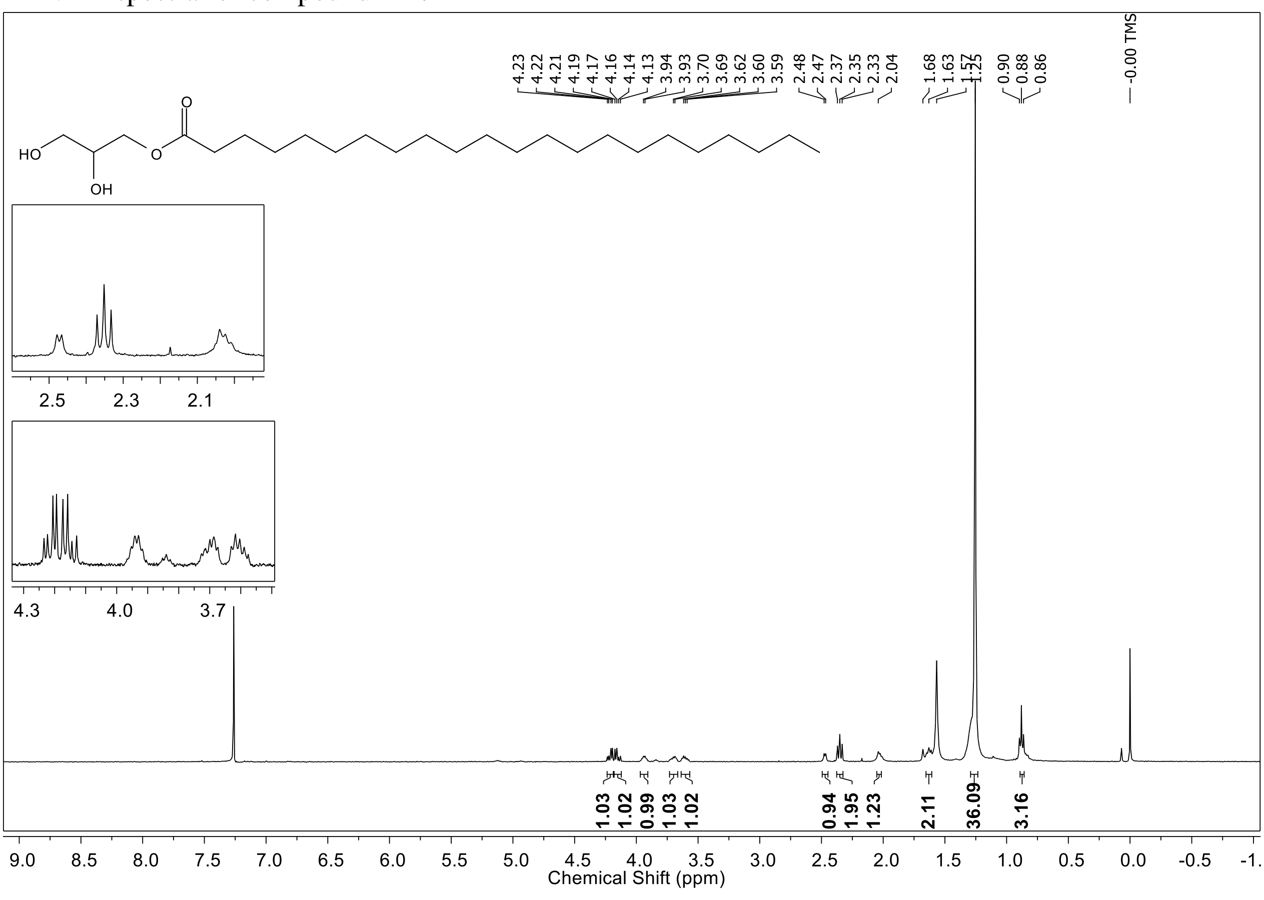

^{13}C NMR spectra for compound **1k'**:

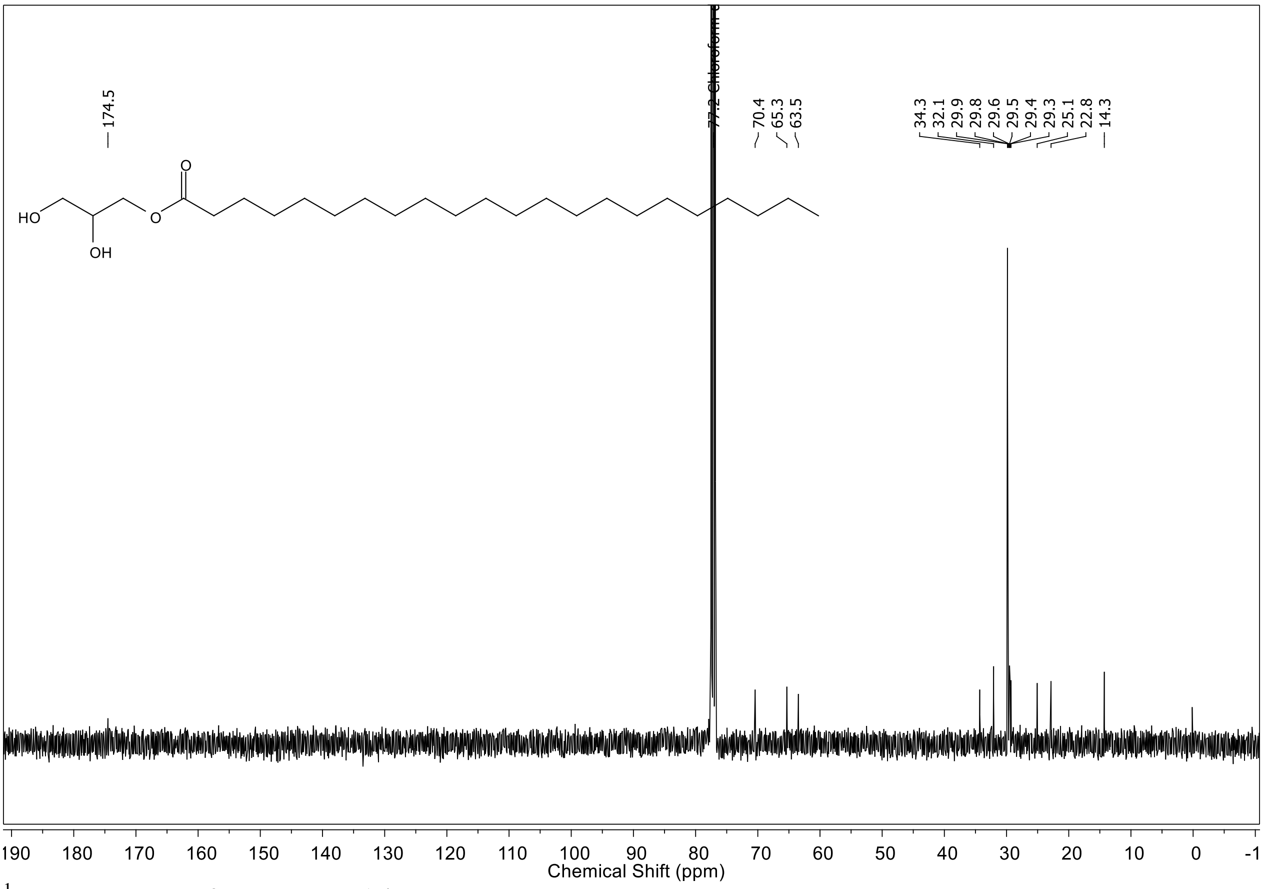

^{1}H NMR spectra for compound **1l**:

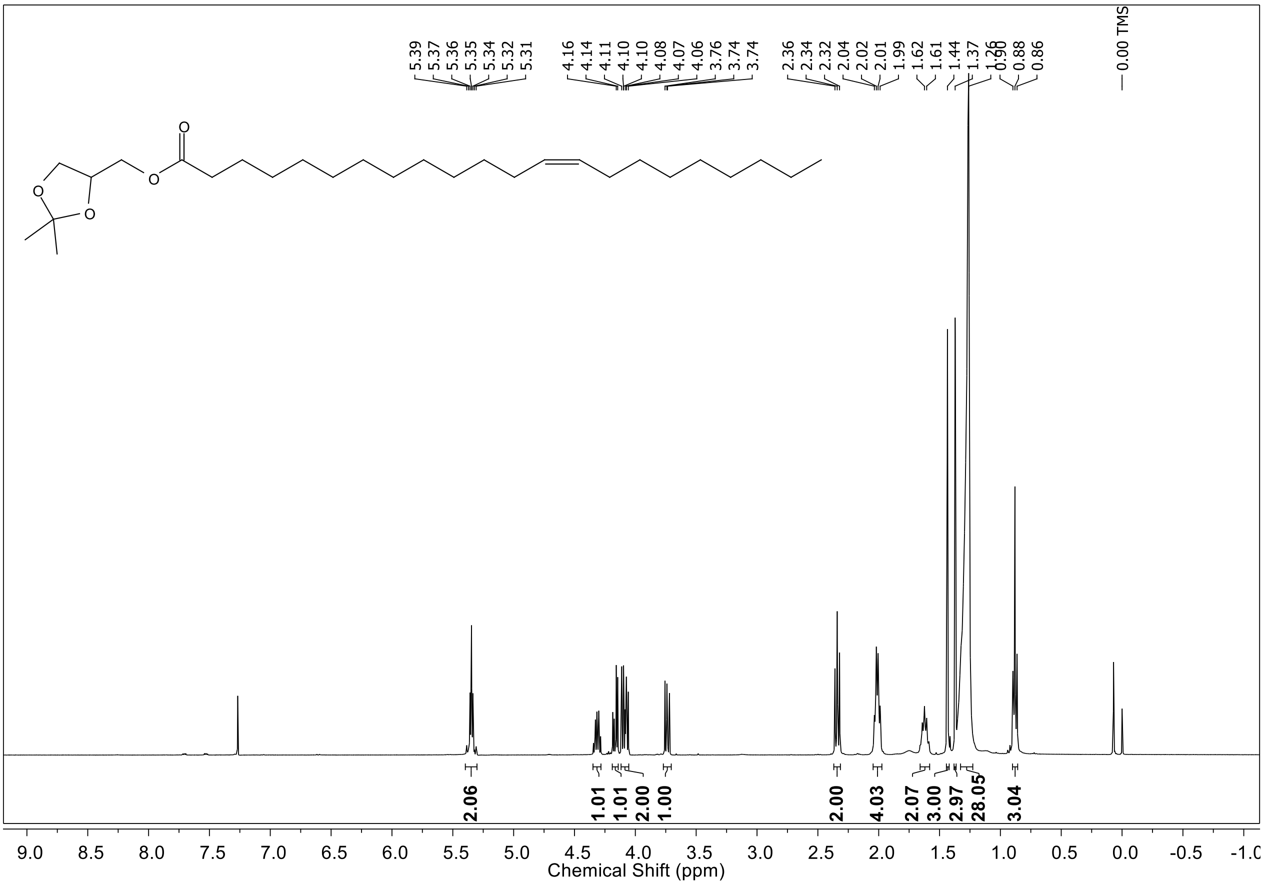

^{1}H NMR spectra for compound **1l'**:

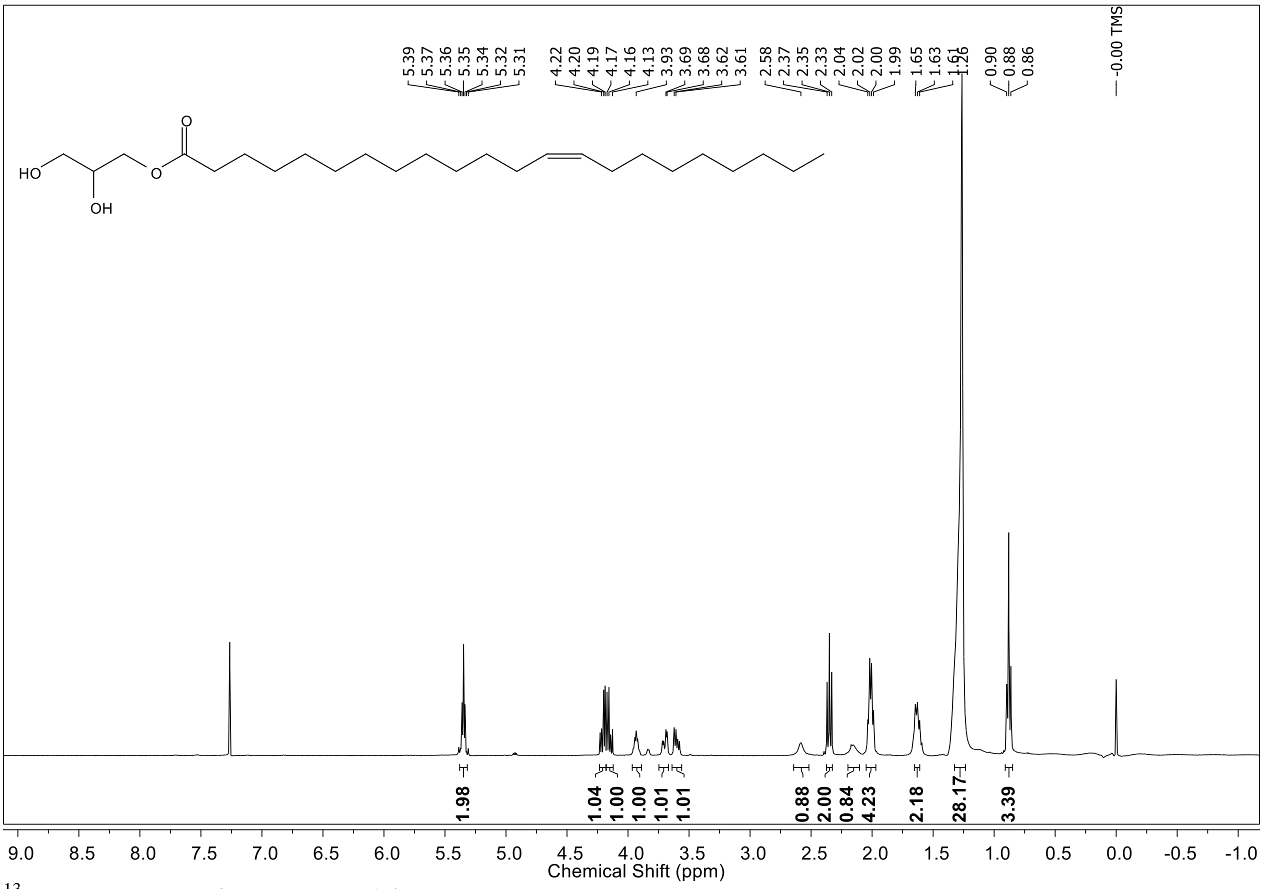

^{13}C NMR spectra for compound **1l'**:

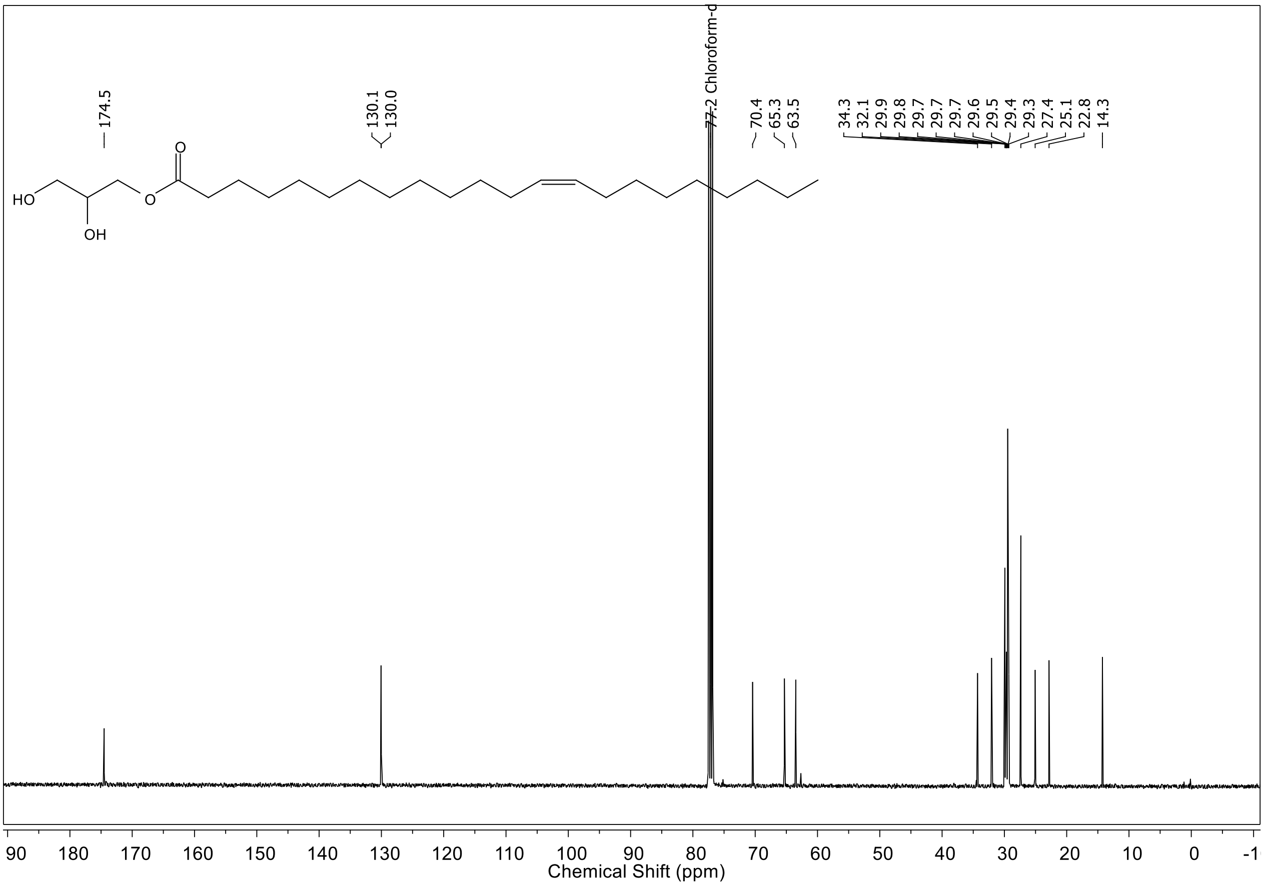

^{1}H NMR spectra for compound **1m’:**

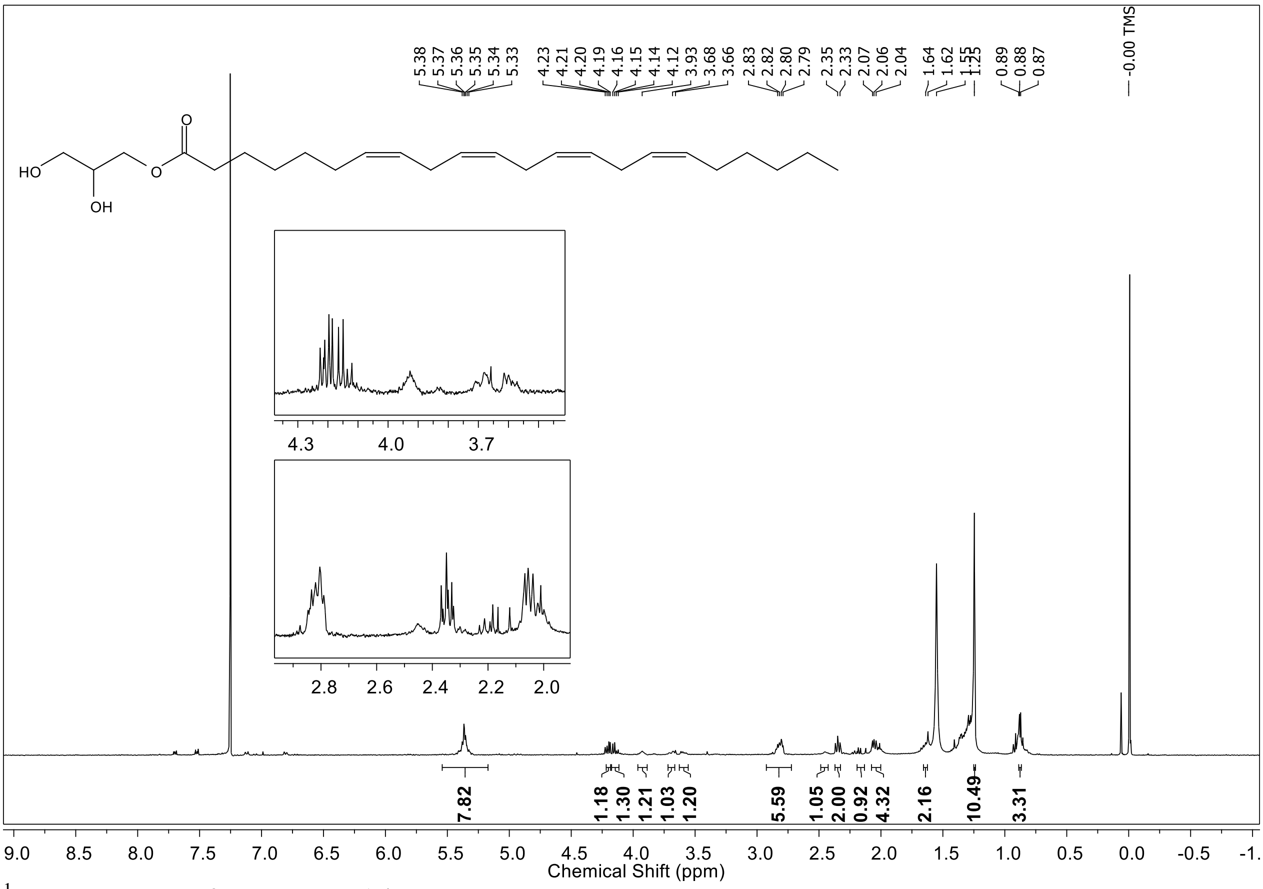

^{1}H NMR spectra for compound **1n:**

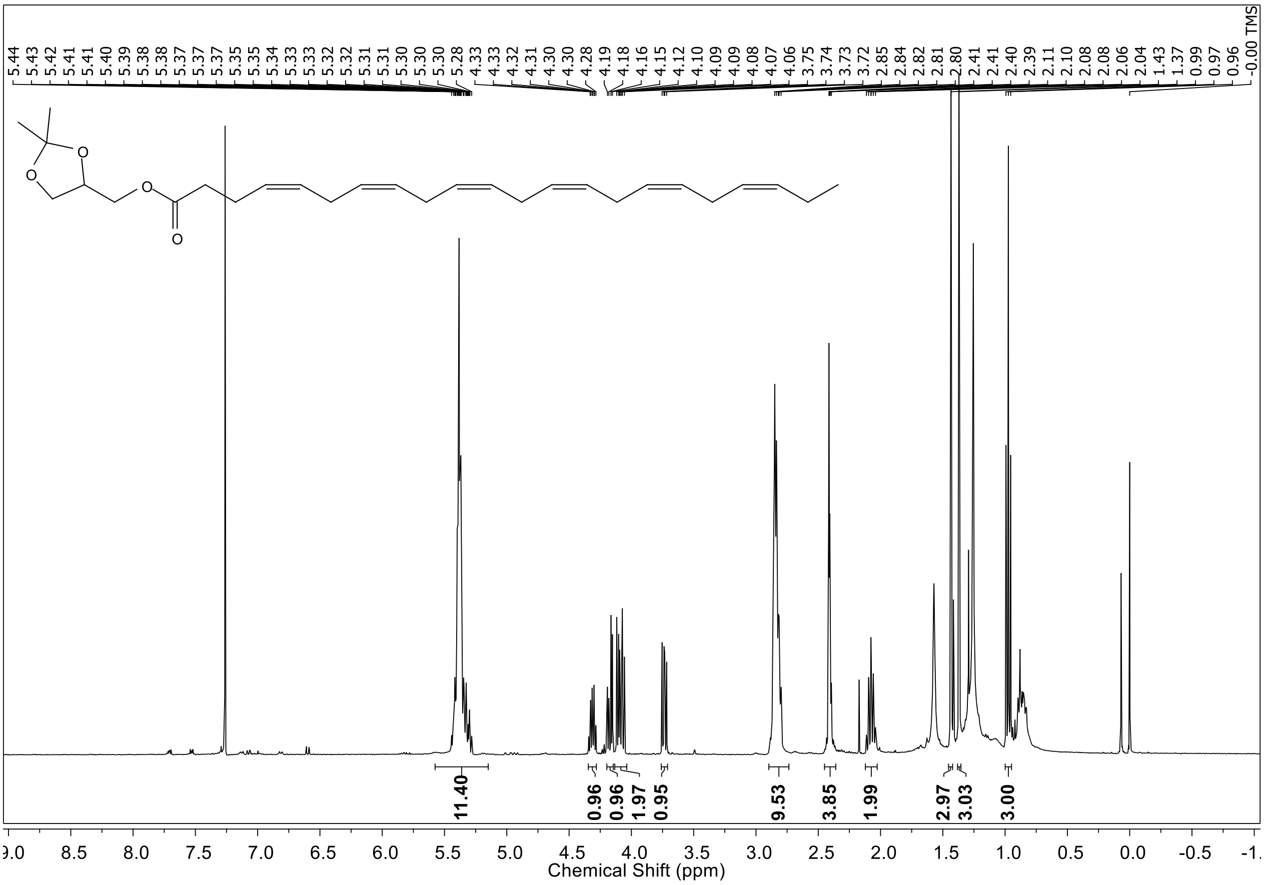

^{1}H NMR spectra for compound **1n'**:

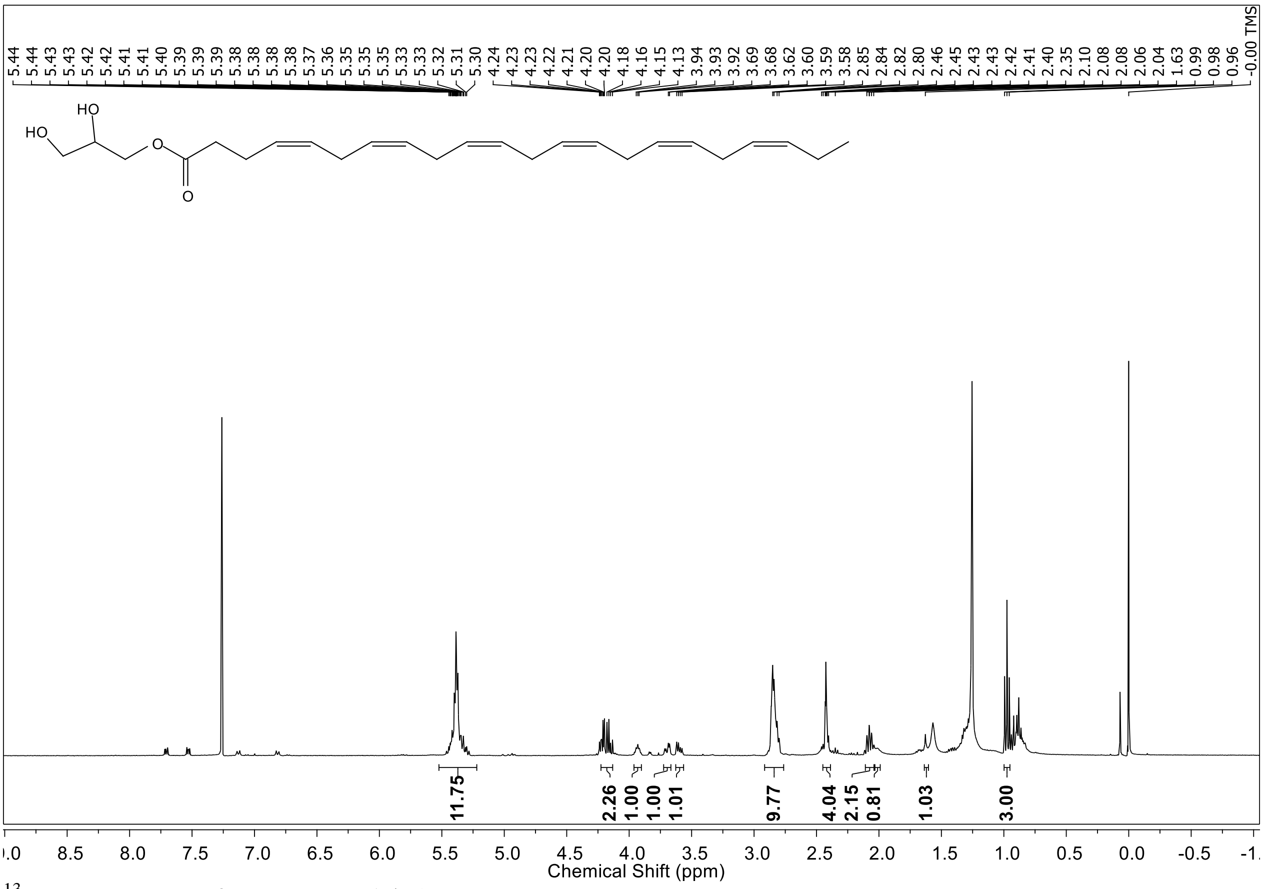

^{13}C NMR spectra for compound **1n'**:

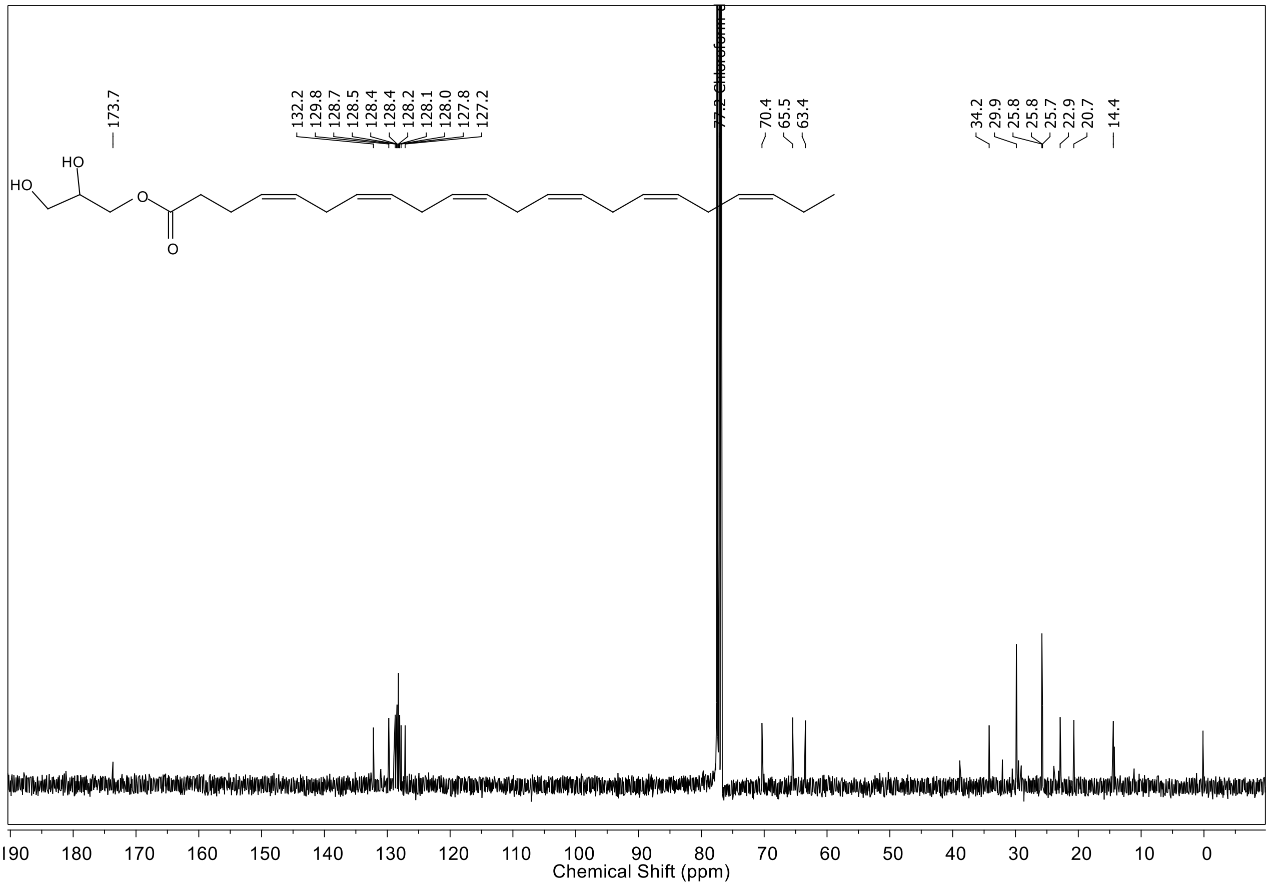

^{1}H NMR spectra for compound **1o:**

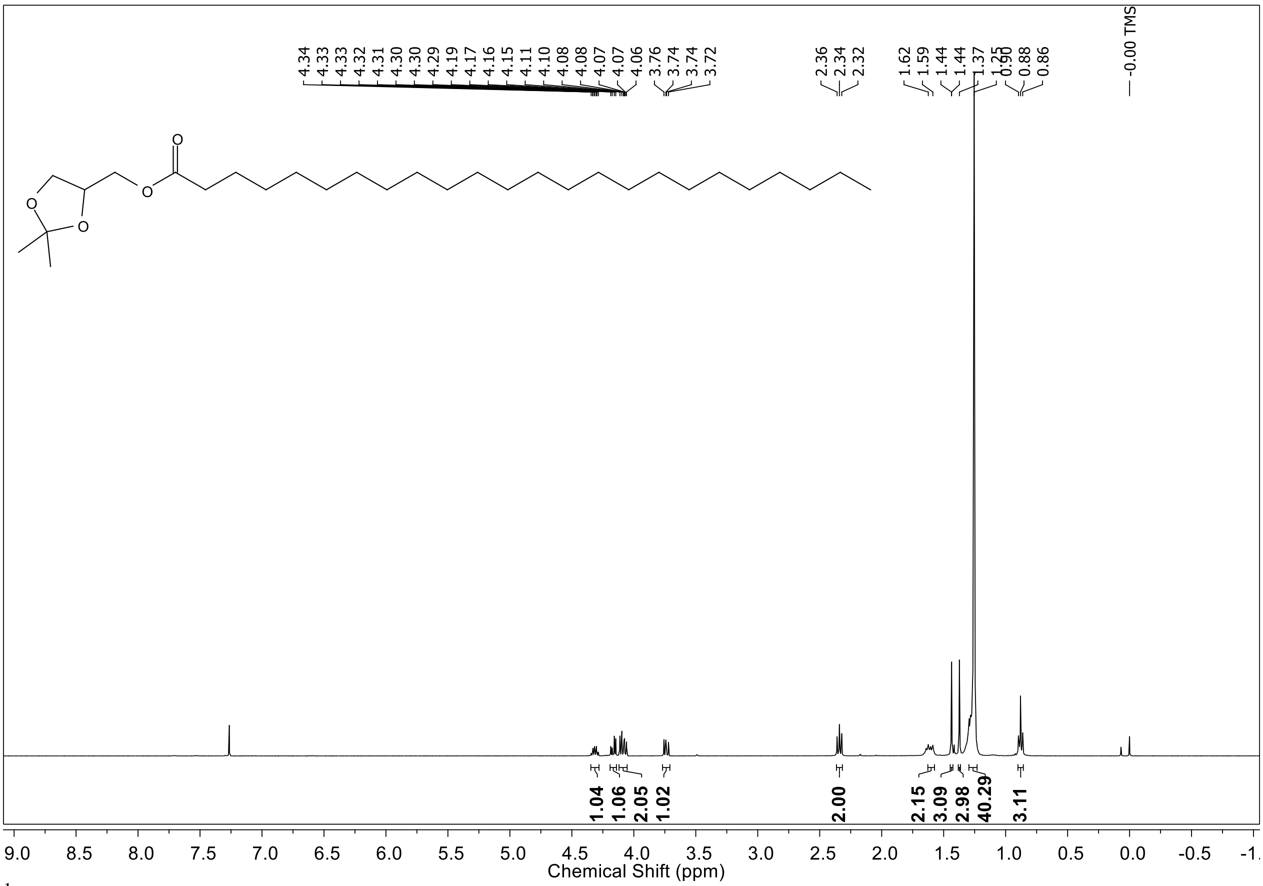

^{1}H NMR spectra for compound **1o':**

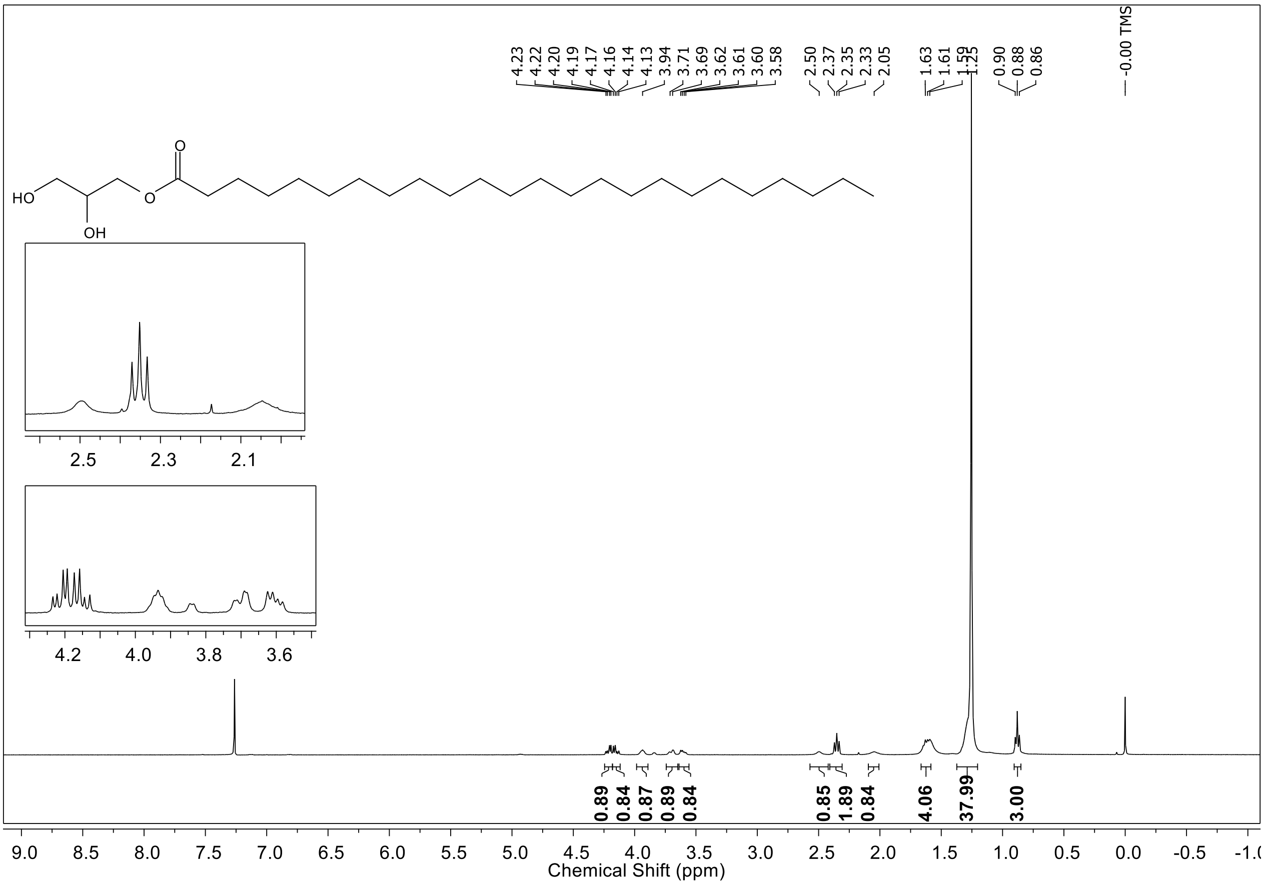

^{13}C NMR spectra for compound **1o'**:

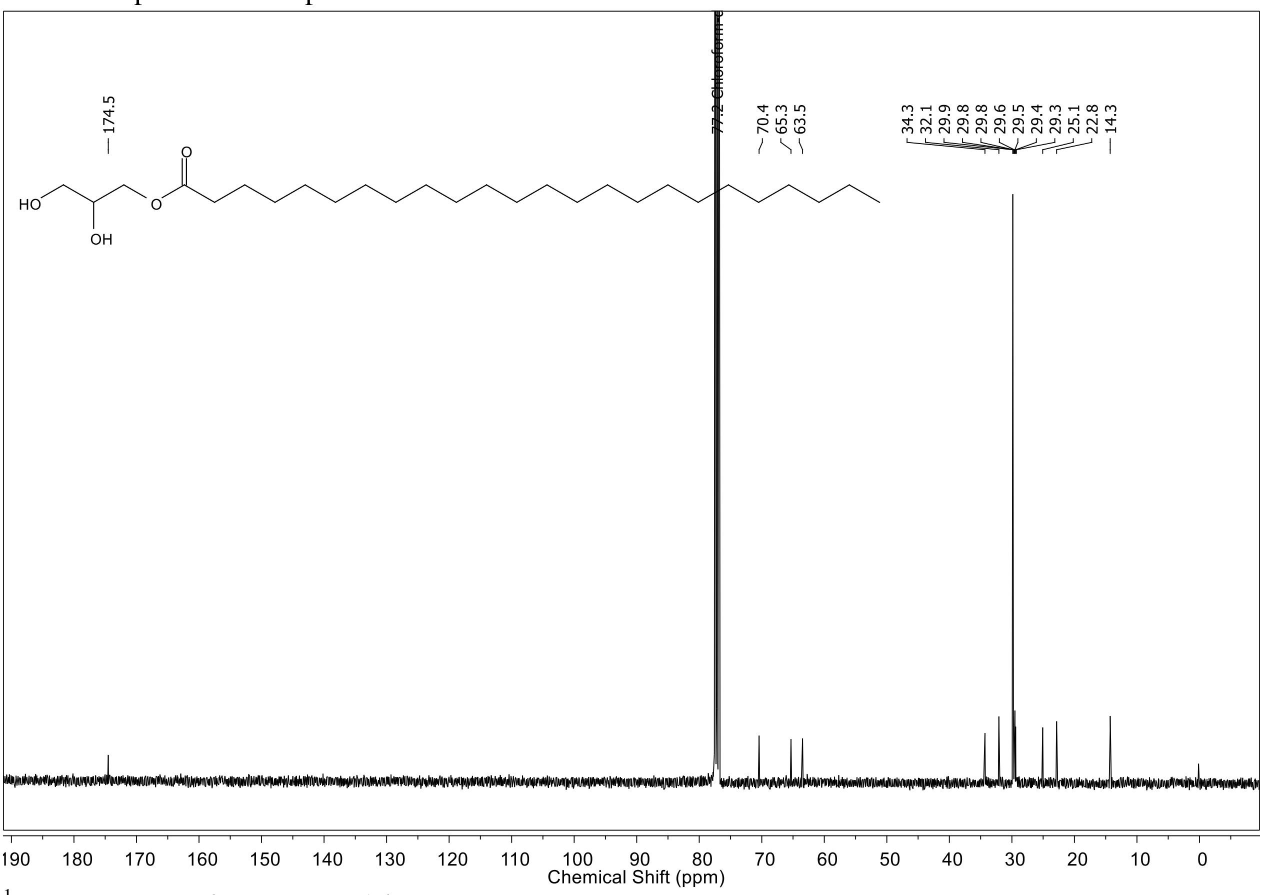

^{1}H NMR spectra for compound **1p**:

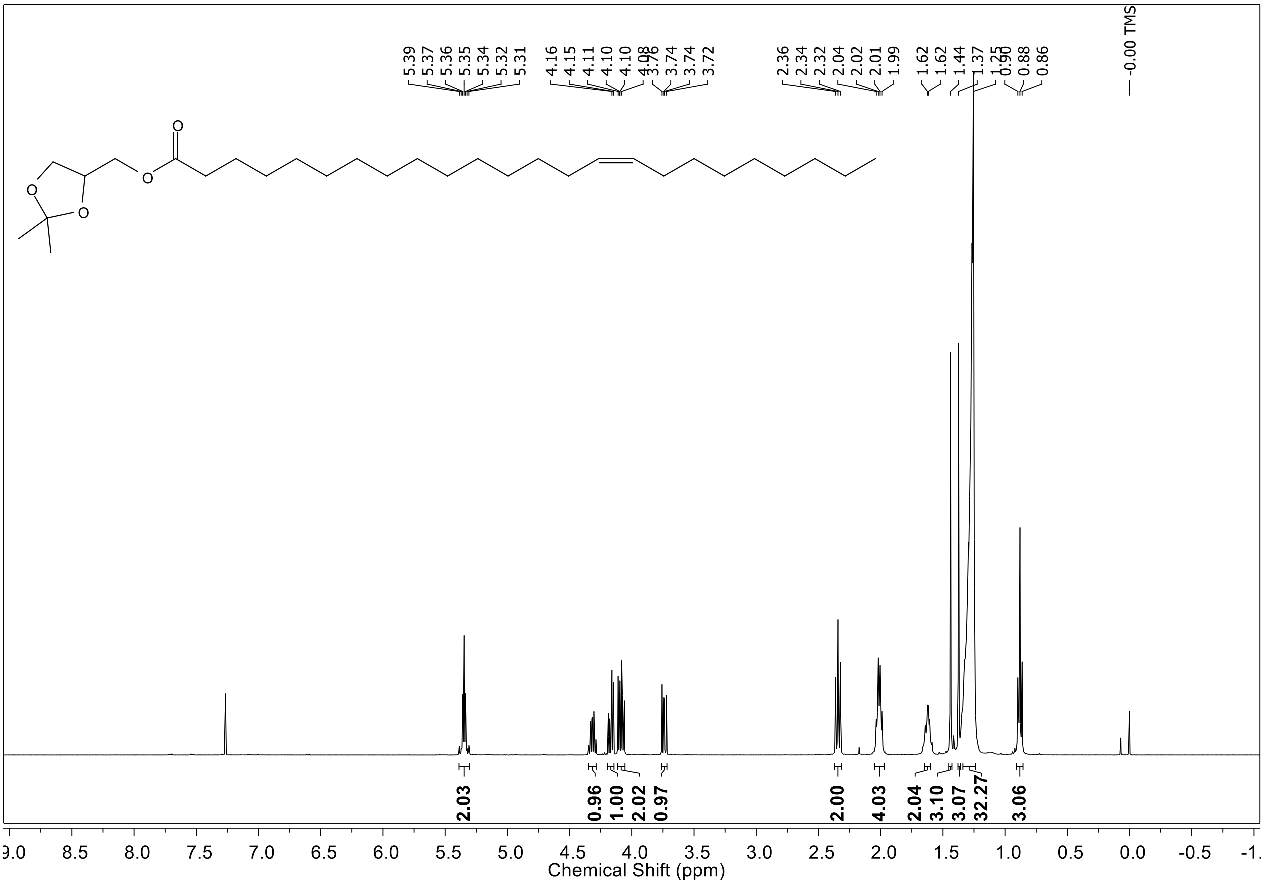

^{1}H NMR spectra for compound **1p’:**

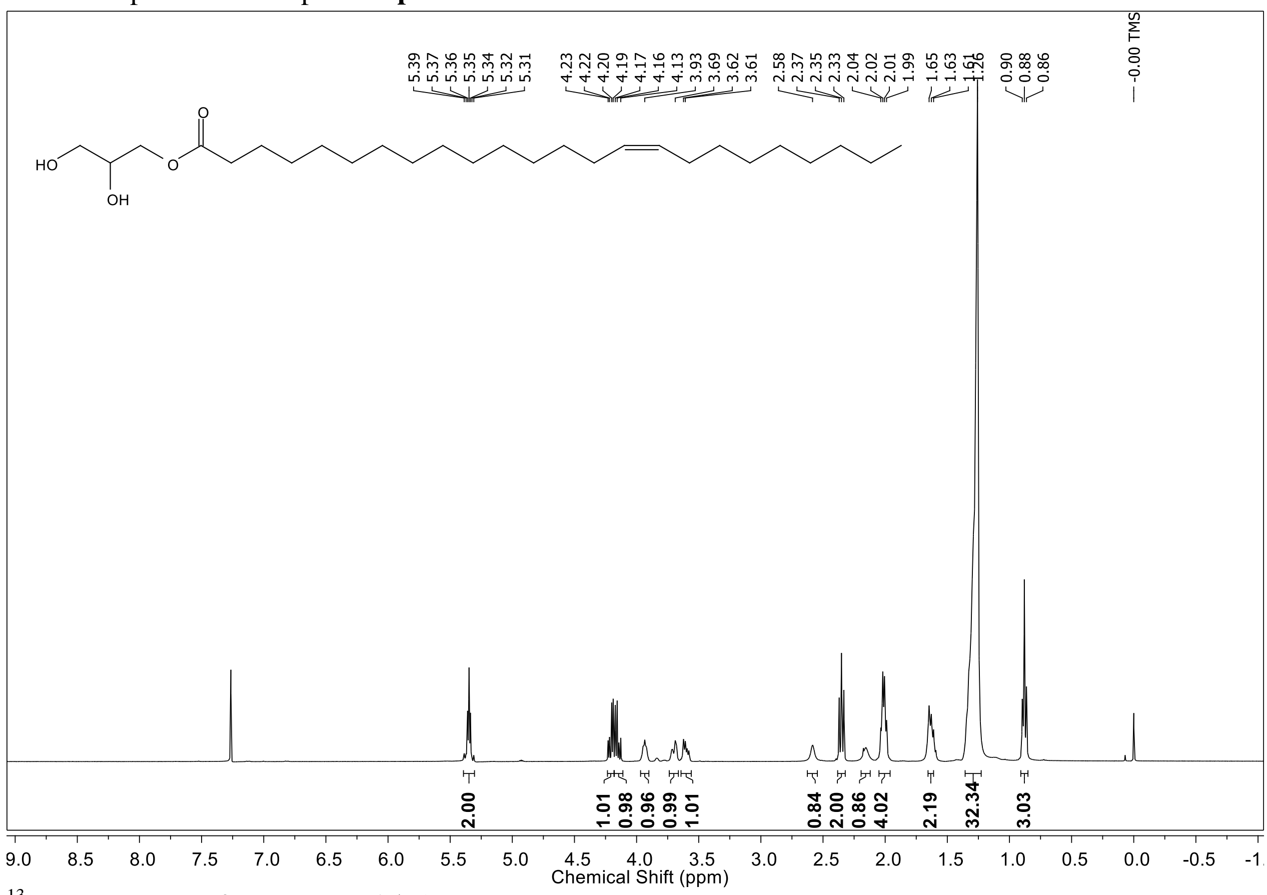

^{13}C NMR spectra for compound **1p’:**

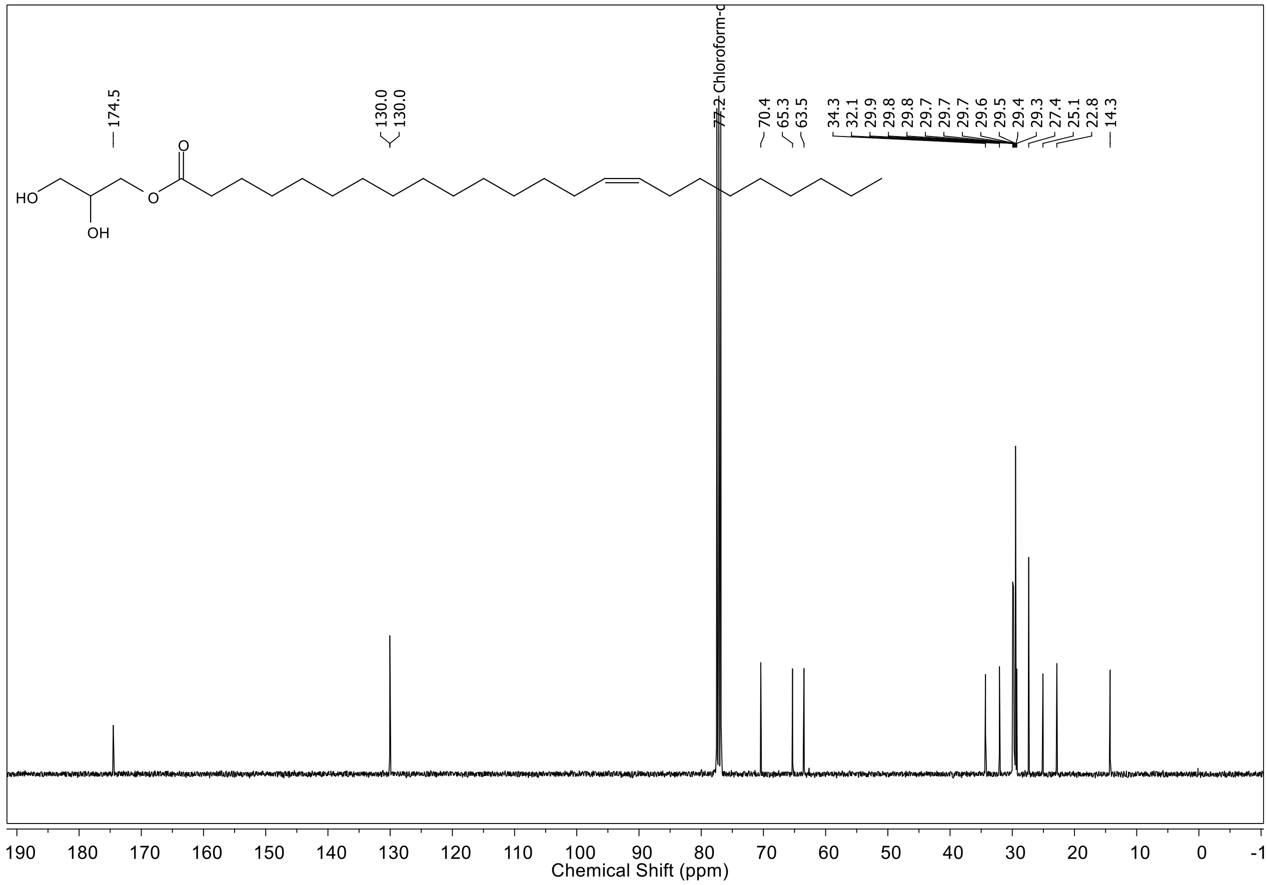

Supplement: Supporting Information [file supp_RA118.005640_140484_1_supp_203916_pf6z84.pdf]
